# Supplementary material for: A novel human fetal lung-derived alveolar organoid model reveals mechanisms of surfactant protein C maturation relevant to interstitial lung disease
Source: EMBO J. 2025 Jan 15;44(3):639–64. doi: 10.1038/s44318-024-00328-6 (PMC11790967; doi:10.1038/s44318-024-00328-6)
Supplement: Supplementary file 1 — Appendix [file 44318_2024_328_MOESM1_ESM.pdf]

## Table of Contents

Appendix Figure S1 ........ 2

Appendix Figure S2 ........ 3

Appendix Figure S3 ........ 4

Appendix Figure S4 ........ 5

Appendix Table S1: qRT-PCR primer sequences ........ 6

Appendix Table S2: gRNA sequences ........ 7

Appendix Table S3: Sample information for RNA sequencing ........ 8

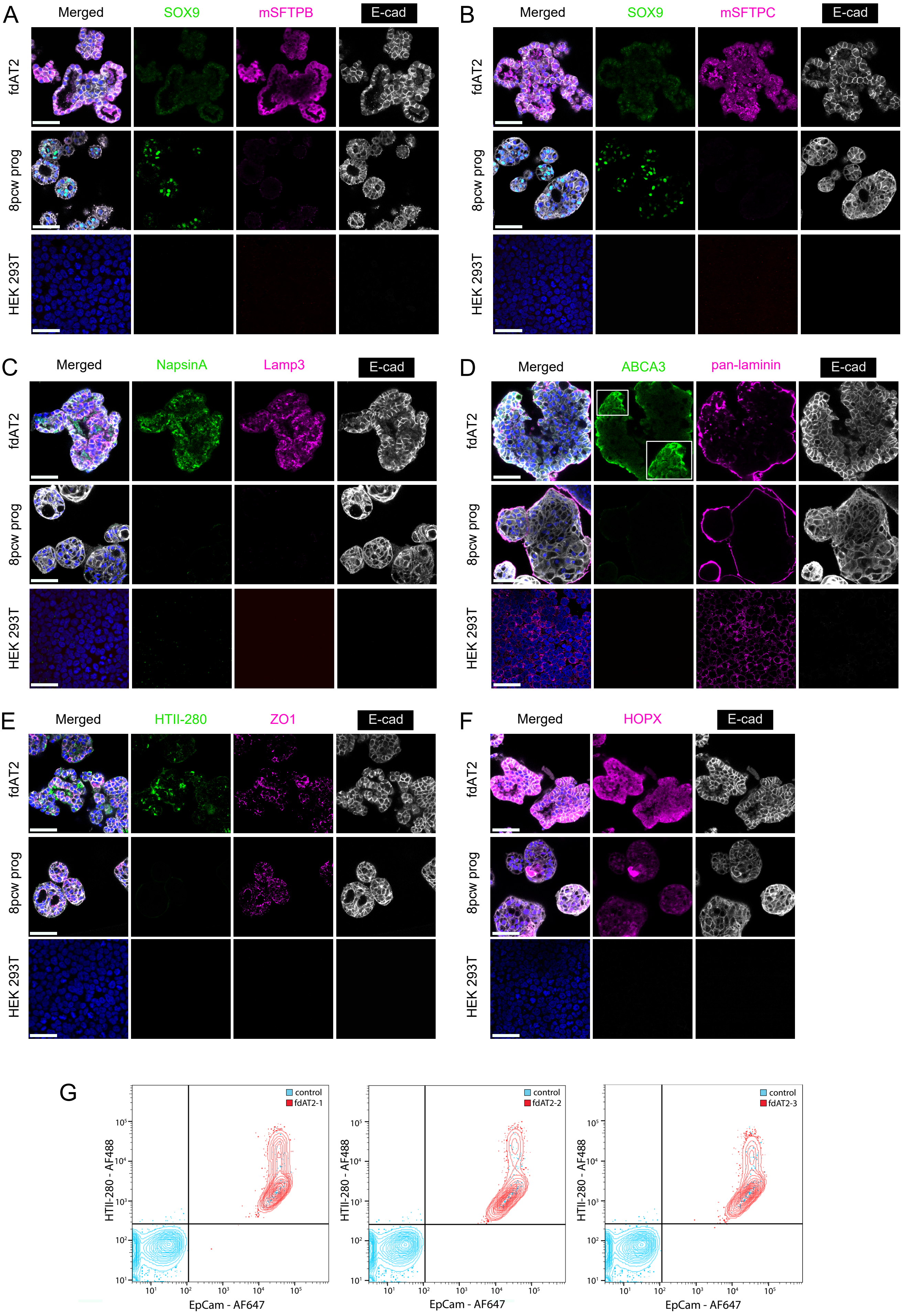


**Appendix Figure S1. Control antibody staining comparing intensity levels in fetal-derived AT2 organoids, 8 pcw tip progenitors and HEK 293T cells.** FdAT2 organoids (passage 19 or 20), 8 pcw tip progenitor organoids (passage 10 or 11) and Human embryonic kidney 293 (HEK 293T) cells were immunostained. Images are shown as individual z planes. (A) SOX9 (green), mature SFTPB (mSFTPB, magenta) and E-cadherin (grey). (B) SOX9 (green), mature SFTPC (mSFTPC, magenta) and E-cadherin (grey). (C) NapsinA (green), LAMP3 (magenta) and E-cadherin (grey). (D) ABCA3 (green), pan-Laminin (magenta) and E-cadherin (grey). (E) HTII-280 (green), ZO1 (magenta) and E-cadherin (grey). (F) HOPX (magenta) and E-cadherin (grey). Scale bar, 50μm. (G) Flow cytometry analysis of three independent fdAT2 lines (fdAT2-(1-3)) for cell surface expression of EpCam and HTII-280 (control = fdAT2 stained with secondary antibody only).

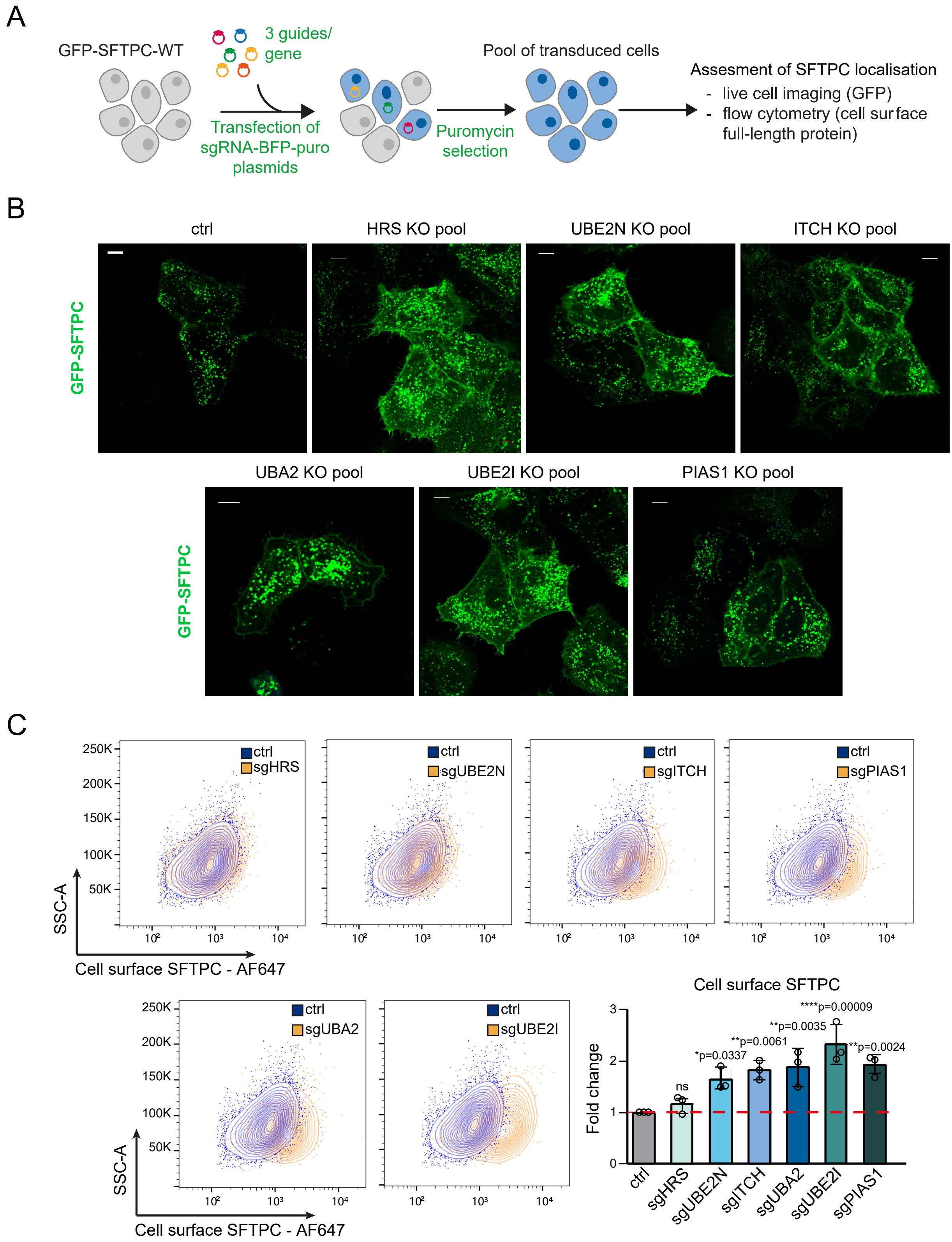


**Appendix Figure S2. Initial validation of screen hits.** (A) Schematic of screen hit validation strategy. (B) Relocalisation of SFTPC as assessed by live cell confocal microscopy in HeLa cells depleted of specific proteins of interest. Scale bar, 10µm. (C) Knockout pools were assessed for cell surface full length SFTPC enrichment by flow cytometry as measured by C-terminal antibody binding and mean fluorescence intensity quantified; mean ± SD, n=3 independent repeats (one-way ANOVA with Dunnetts multiple comparison test).

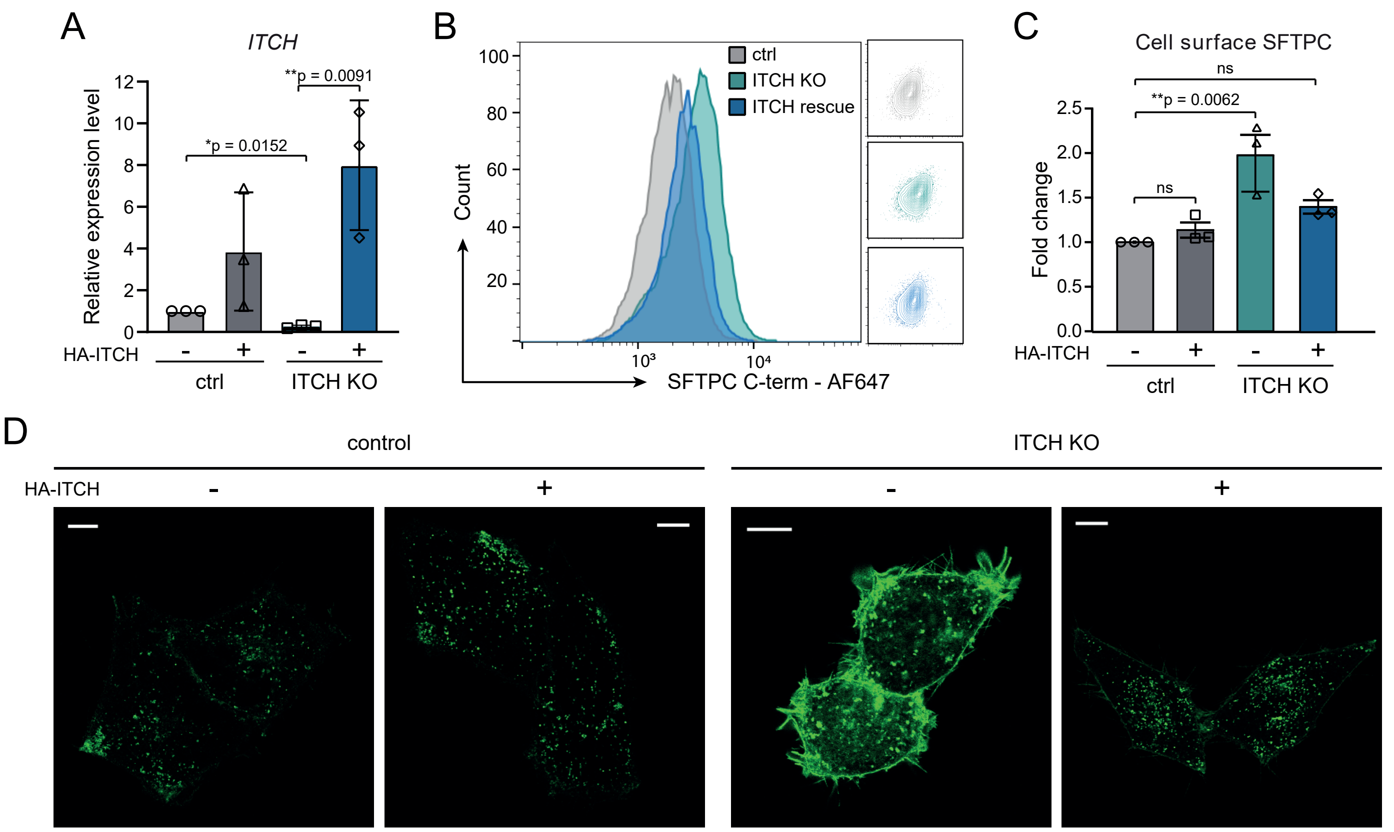


**Appendix Figure S3. Restoration of ITCH expression in knockout cells reverses SFTPC mislocalisation.** Control and ITCH knockout HeLa cells transfected with HA-ITCH were assessed for relative ITCH mRNA expression (A) and cell surface full-length SFTPC protein by flow cytometry (B&C); mean ± SD, n=3 independent repeats (one-way ANOVA with Tukey multiple comparison post-test);. (D) GFP-SFTPC localisation following ITCH depletion and rescue. Scale bar, 10µm.

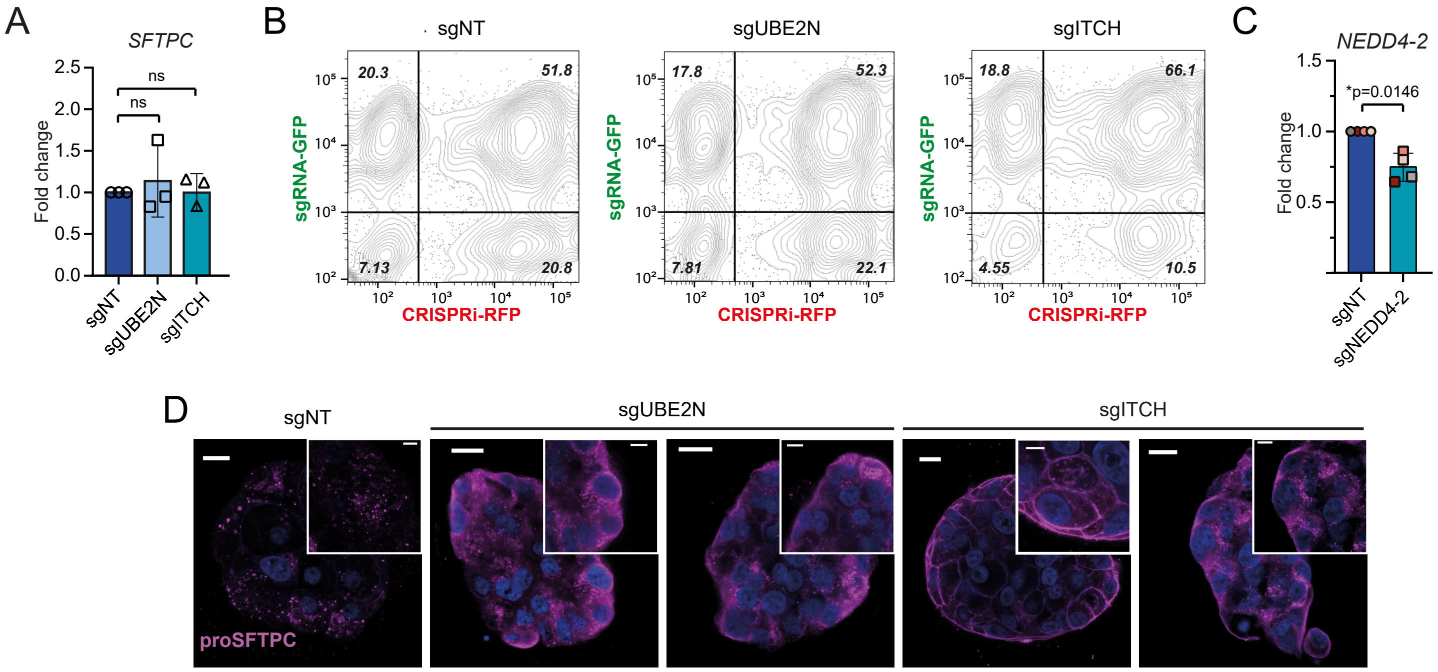


**Appendix Figure S4. FdAT2 CRISPRi additional data.** (A) Flow cytometry dot plots of sgRNA-GFP positive (y-axis) and CRISPRi-RFP (x-axis) positive populations in fdAT2 organoids transduced with sg non-targeting (NT), sgUBE2N or sgITCH at cell harvesting (5 days post induction of CRISPRi system). Dual positive populations varied by organoid line and ranged between 50 and 80%. (B) Relative SFTPC expression in fdAT2 depleted of UBE2N or ITCH; mean ± SD, n=3 biologically independent organoid lines repeats (paired two-tailed Student's t-test). (C) Relative NEDD4-2 expression in fdAT2 depleted of NEDD4-2; mean ± SD, n=3 biologically independent organoid lines repeats (one sample t-test). (D) Additional representative images of SFTPC localisation and intensity in fdAT2 organoids depleted of ITCH or UBE2N with exposure optimised to show SFTPC subcellular localisation. Scale bar, 10µm / 5µm in zoomed inserts.

## Appendix Table S1: qRT-PCR primer sequences

| Gene assession number | Gene name | Forward (5'-3') | Reverse (5'-3') |
|---|---|---|---|
| NM_001101 | *ACTB* | TGCCCTCAACGACCACTTTG | GGGTCTCTCTCTTCCTCTTGTGCT |
| NM_001172357 | *SFTPC* | CTCCACATGAGCCAGAAACACAC | GTAGTCATACACCACGAGGCCAGT |
| NM_003317 | *NKX2.1* | CGTACCAGGACACCATGAGGAA | CGTCGCTCCAGCTCGTACAC |
| NM_001089 | *ABCA3* | CACCTCAAGAGCAAGTTCGGCAG | GGACCATGCCTTGGTGCTCATC |
| NM_014398 | *LAMP3* | AACAACCGATGTCCAACTTCAAGCC | CCCATACCCATAAGGCAGAGACCAA |
| NM_001172357 | *SFTPC* | GTCCTCATCGTCGTGGTGATTG | AGAAGGTGGCAGTGGTAACCAG |
| NM_002046 | *GAPDH* | GAAGGTGAAGGTCGGAGTC | GAAGATGGTGATGGGATTTC |
| NM_001257137 | *ITCH* | GAGGTCACAGTAGATGGACAG | GCAGTTCCCAACAAAACATCAG |
| NM_003348 | *UBE2N* | AGGATCATCAAGGAAACCCAG | CAAGTTTAAAAGTCCCTCCCTC |

## Appendix Table S2: gRNA sequences

| | |
|---|---|
| **Forward genetic screen validation** | |
| ITCH (guide 1) | GAACGGCGGGTTGACAACAT |
| ITCH (guide 2) | ATTGTGTAATTCGTGTGTTG |
| ITCH (guide 3) | AATACAAACCTGGTCTACGT |
| UBE2N (guide 1) | CTGTTGCCTTCATAGATAAG |
| UBE2N (guide 2) | CCTCAAAGGGGGAATCCTGA |
| UBE2N (guide 3) | CCTTCCAGAAGAATACCCAA |
| HRS (guide 1) | GTTCGGGGTGATGACCCGTA |
| HRS (guide 2) | CTTGGGGTACGAAGTGTACG |
| HRS (guide 3) | GCACGCAGCCCCCACCAACG |
| UBA2 (guide 1) | TGACAATGGACAAACTATGG |
| UBA2 (guide 2) | ACAGCCAGGAAAGGTTCTCT |
| UBA2 (guide 3) | TTGTAGCCCTGACTATAATG |
| UBE2I (guide 1) | ACAGGCACACTGTCCCCGAA |
| UBE2I (guide 2) | AAGCCCTCCTTACAAATGGG |
| UBE2I (guide 3) | AAGGAGGCTTGTTTAAACTA |
| PIAS1 (guide 1) | ACGCAAGATGGCGGACAGTG |
| PIAS1 (guide 2) | CTTAAAGCTCTAGAATGATC |
| PIAS1 (guide 3) | CCTCTGAAGAAGAACTGTTG |
| **CRISPRi** | |
| ITCH | GGGAGTGGGACGCGCGGTTC |
| UBE2N | GACGCACGAGTGGAAGTCCC |
| Non-targeting | GCTGATCTATCGCGGTCGTC |

## Appendix Table S3: Sample information for RNA sequencing

| Human fetal lung_ID | Developmental age | Sample name | Early passage bulk RNAseq | Late passage bulk RNAseq | Single cell RNA seq |
|---|---|---|---|---|---|
| HDBR-L 15934 | 17 pcw | AT2_1 | P7 | P16 | P16 |
| HDBR-L 16011 | 21 pcw | AT2_2 | P6 | P17 | P11 |
| HDBR-L 16392 | 17 pcw | AT2_3 | P1 | P12 | P15 |
| HDBR-L 16402 | 20 pcw | AT2_4 | P4 | P13 | P16 |
