# Supplementary material for: A novel human fetal lung-derived alveolar organoid model reveals mechanisms of surfactant protein C maturation relevant to interstitial lung disease
Source: EMBO J. 2025 Jan 15;44(3):639–64. doi: 10.1038/s44318-024-00328-6 (PMC11790967; doi:10.1038/s44318-024-00328-6)
Supplement: Supplementary file 7 — Movie EV1 [file 44318_2024_328_MOESM7_ESM.zip › Movie EV1_Legend.docx]

**Movie EV1.** FDAT2 organoid confocal z stack, stained for mature SFTPC (red), E-cadherin (green).
