# Supplementary material for: A novel human fetal lung-derived alveolar organoid model reveals mechanisms of surfactant protein C maturation relevant to interstitial lung disease
Source: EMBO J. 2025 Jan 15;44(3):639–64. doi: 10.1038/s44318-024-00328-6 (PMC11790967; doi:10.1038/s44318-024-00328-6)
Supplement: Supplementary file 8 — Movie EV2 [file 44318_2024_328_MOESM8_ESM.zip › Movie EV2_Legend.docx]

**Movie EV2.** Confocal z stack through mouse PCLS containing transplanted human fdAT2 cells transduced with SFTPC-GFP; EF1a-RFP. Stained for SFTPC (yellow) and CAV1 (white).
