# Supplementary figures and images for: A novel human fetal lung-derived alveolar organoid model reveals mechanisms of surfactant protein C maturation relevant to interstitial lung disease (part 1 of 2)
Source: EMBO J. 2025 Jan 15;44(3):639–64. doi: 10.1038/s44318-024-00328-6 (PMC11790967; doi:10.1038/s44318-024-00328-6)

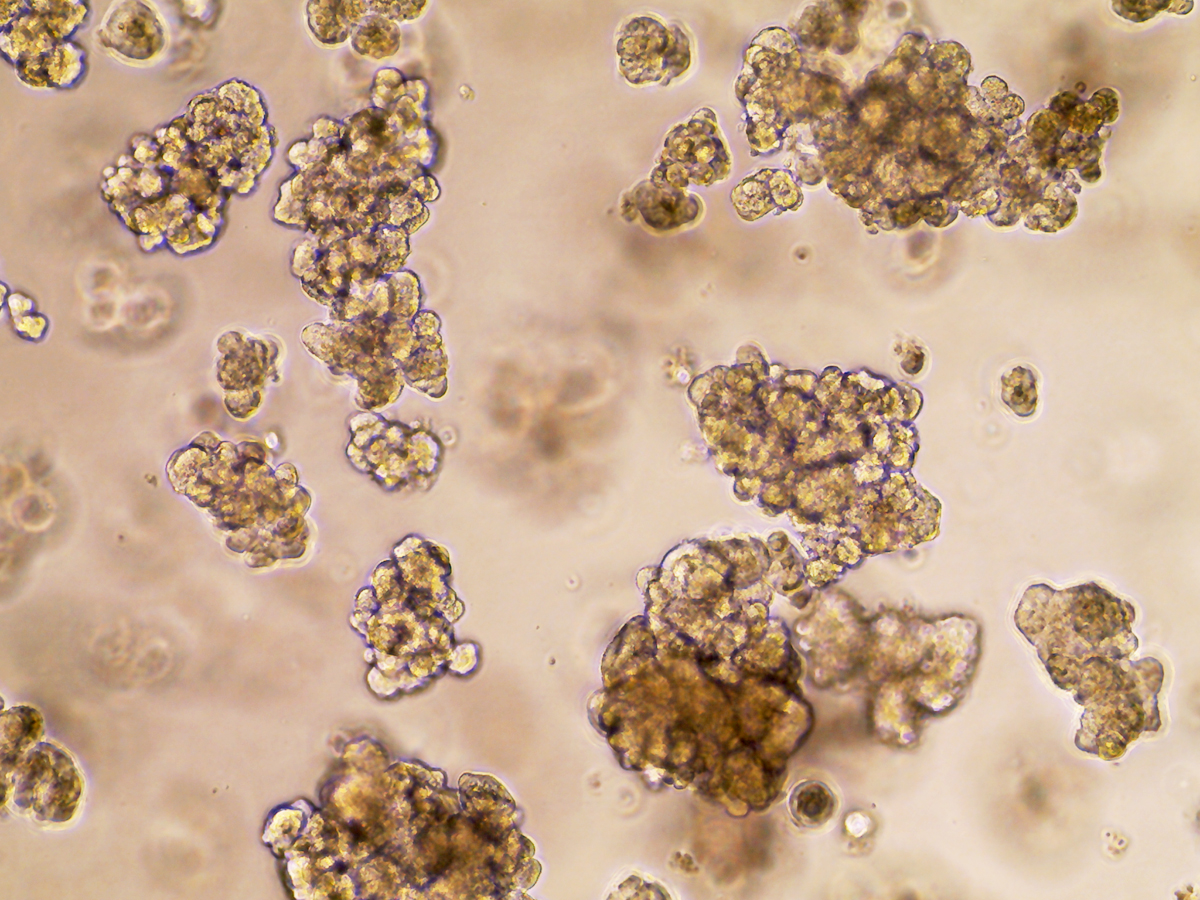

Supplement: Supplementary file 11 — Source data Fig. 1 [file 44318_2024_328_MOESM11_ESM.zip › Figure 1B. Bright field image of fdAT2 organoid.jpg]

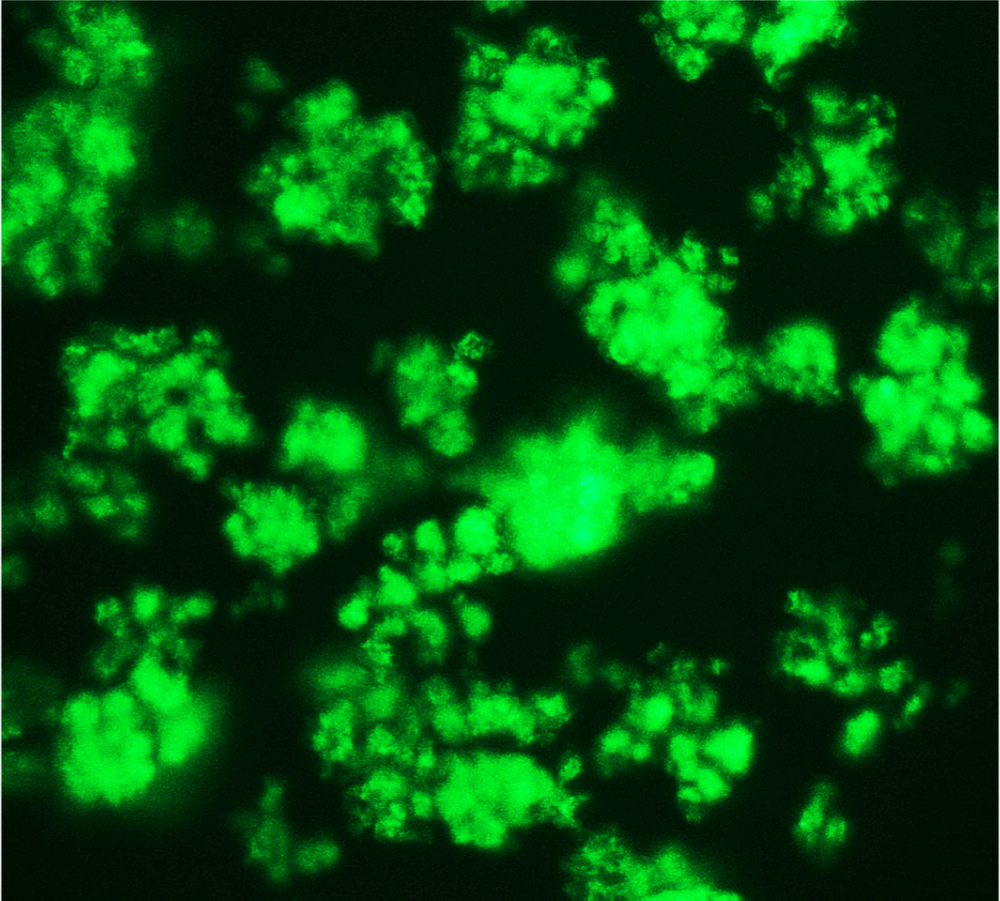

Supplement: Supplementary file 11 — Source data Fig. 1 [file 44318_2024_328_MOESM11_ESM.zip › Figure 1C.Lysotracker-fdAT2 organoids.jpg]

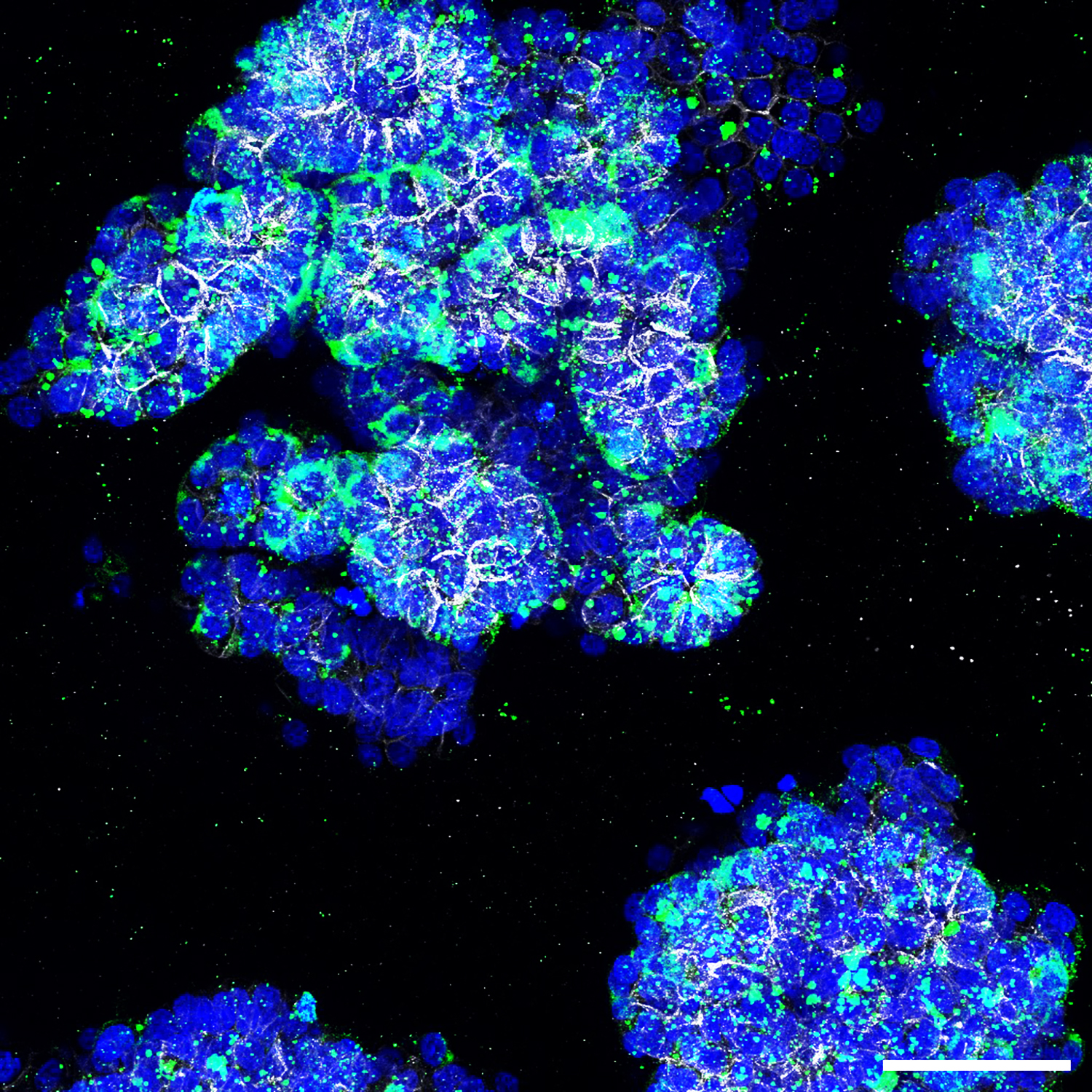

Supplement: Supplementary file 11 — Source data Fig. 1 [file 44318_2024_328_MOESM11_ESM.zip › Figure 1G. ACBA3-Laminin-Ecad_1.jpg]

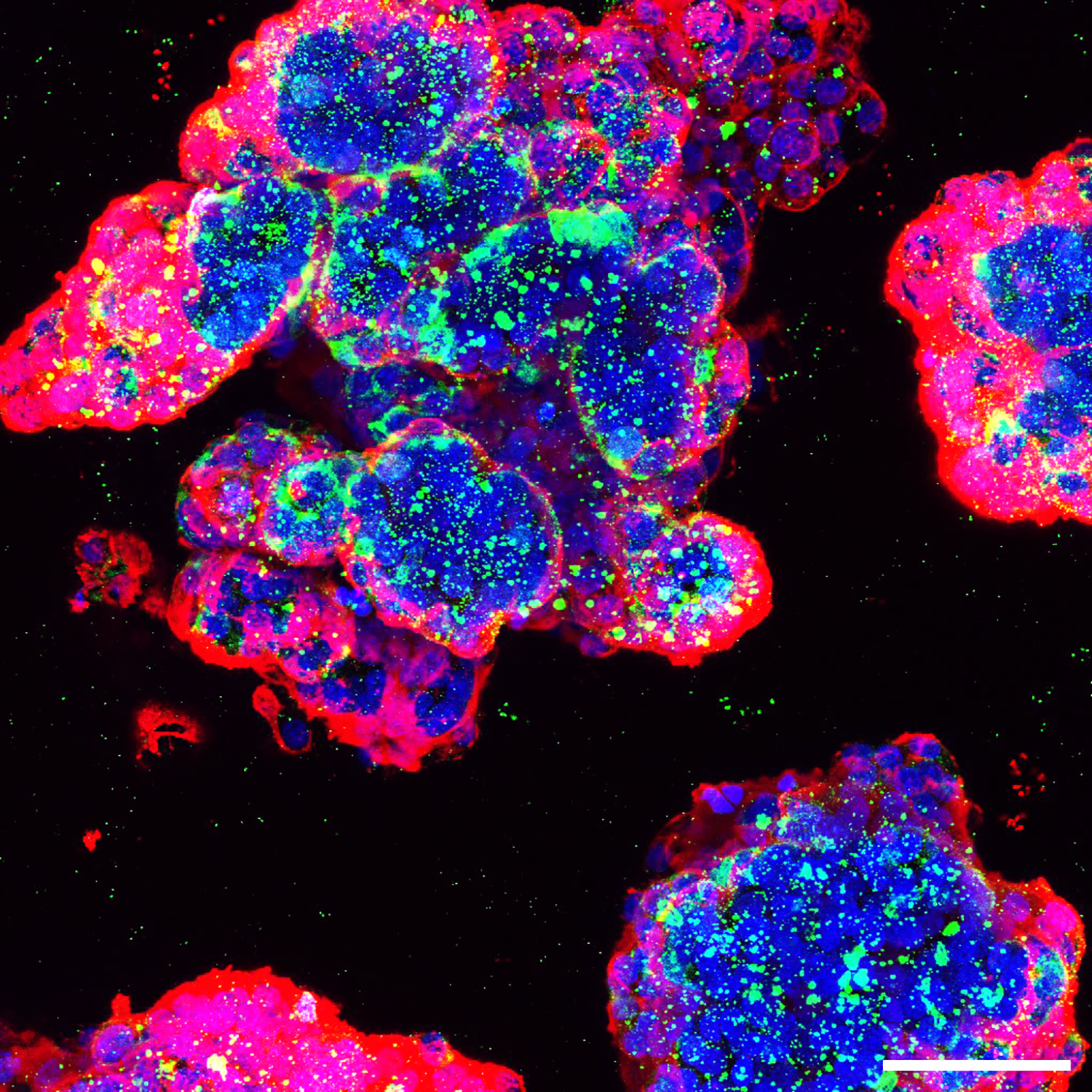

Supplement: Supplementary file 11 — Source data Fig. 1 [file 44318_2024_328_MOESM11_ESM.zip › Figure 1G. ACBA3-Laminin-Ecad_2.jpg]

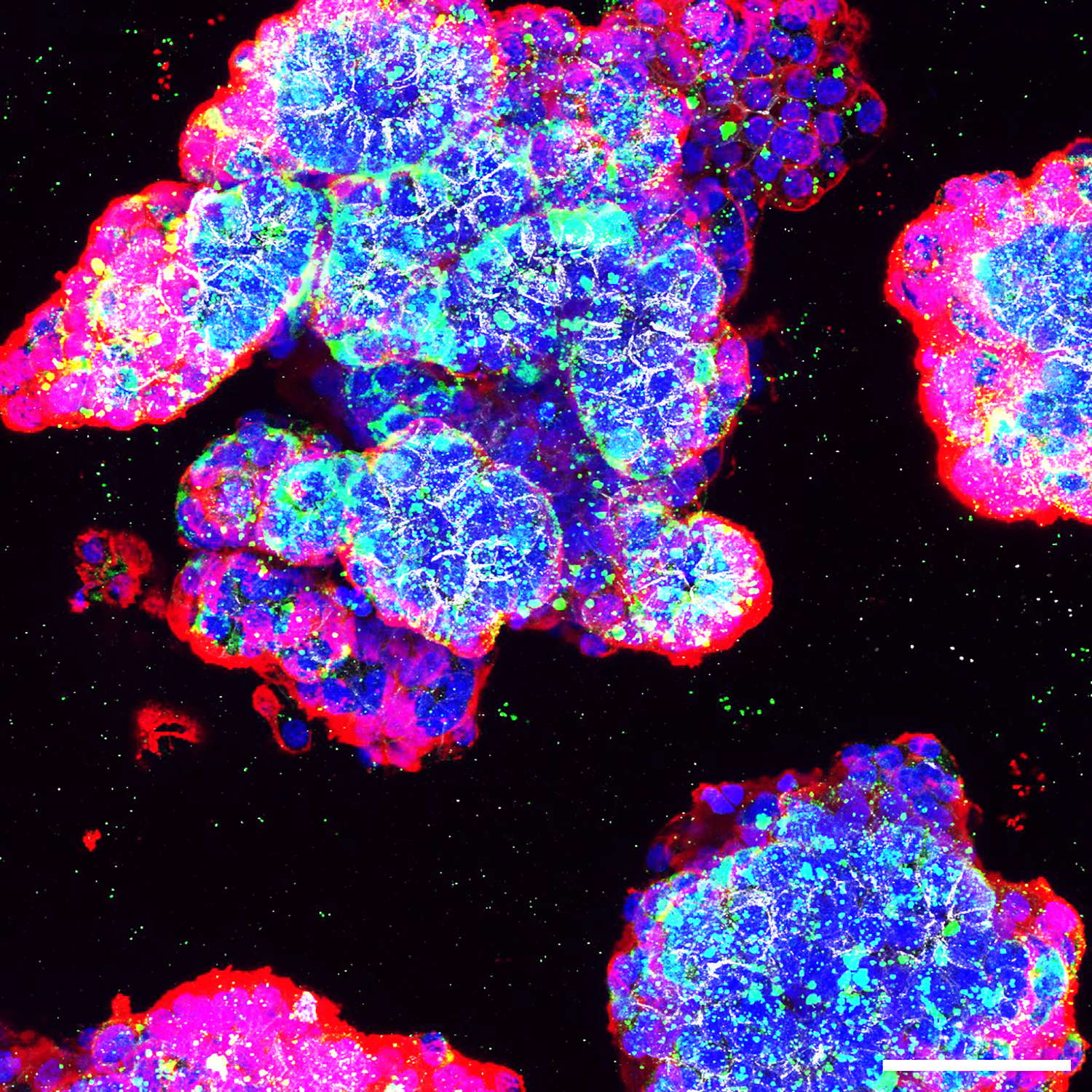

Supplement: Supplementary file 11 — Source data Fig. 1 [file 44318_2024_328_MOESM11_ESM.zip › Figure 1G. ACBA3-Laminin-Ecad_3.jpg]

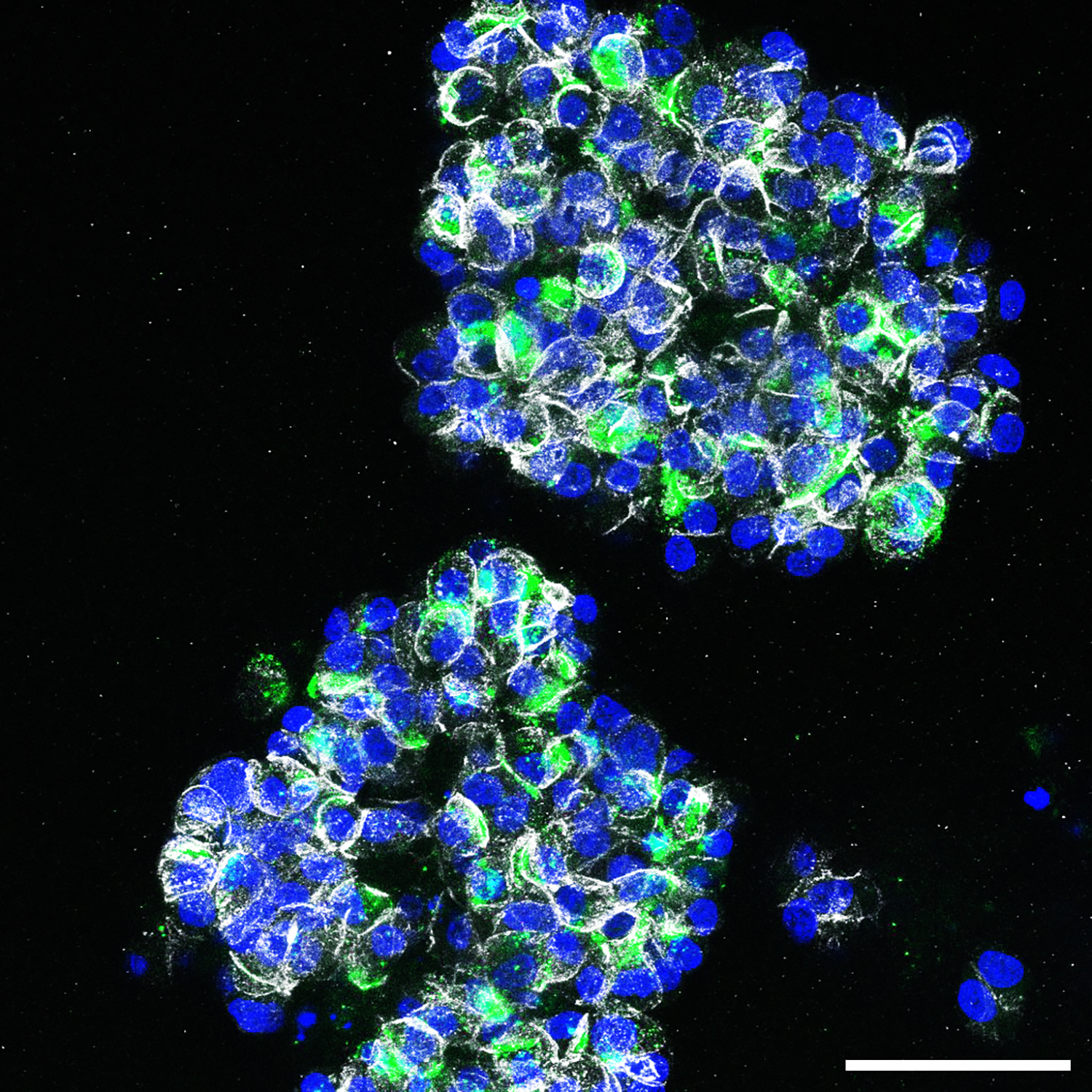

Supplement: Supplementary file 11 — Source data Fig. 1 [file 44318_2024_328_MOESM11_ESM.zip › Figure 1G. HT2-280-HOPX-Ecad_1.jpg]

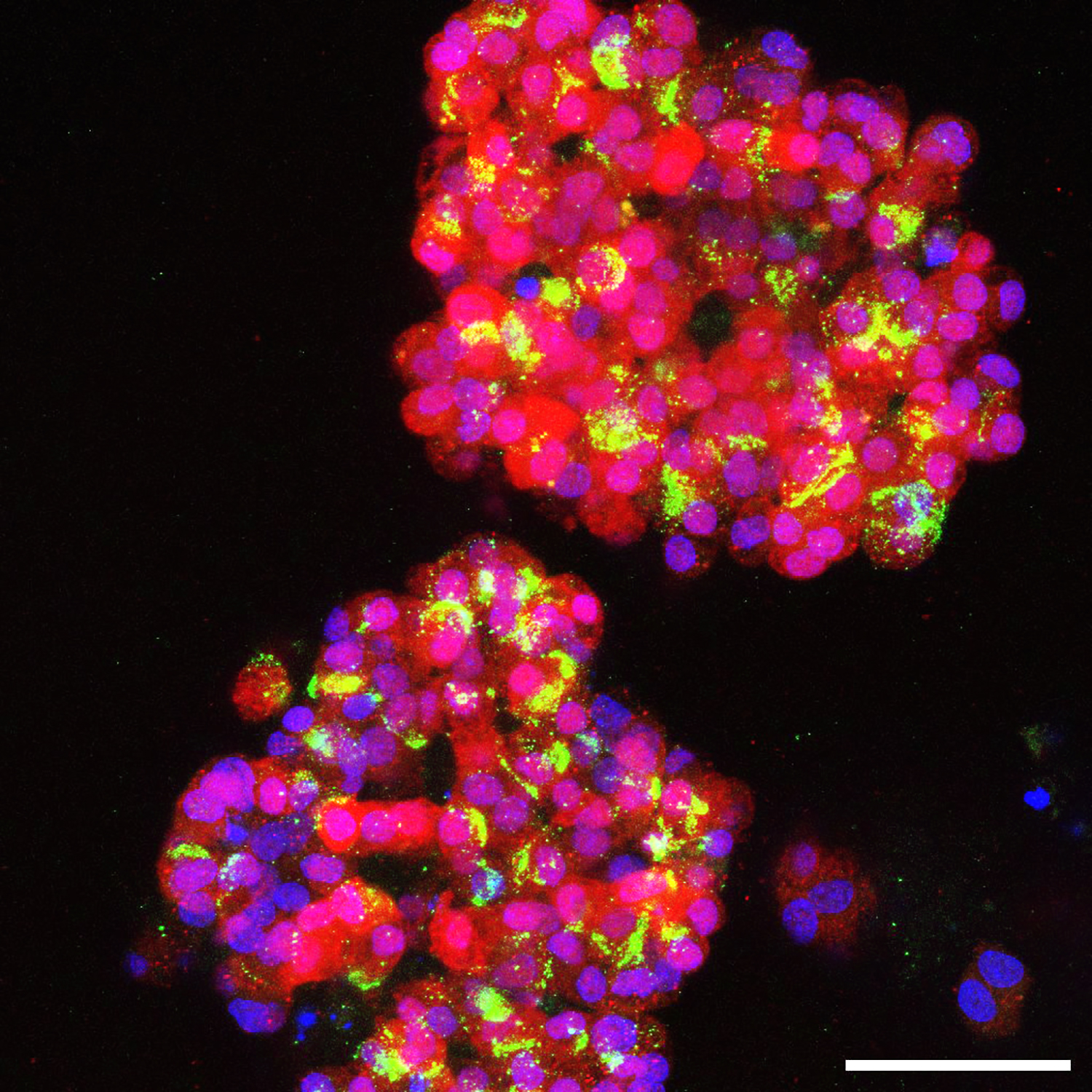

Supplement: Supplementary file 11 — Source data Fig. 1 [file 44318_2024_328_MOESM11_ESM.zip › Figure 1G. HT2-280-HOPX-Ecad_2.jpg]

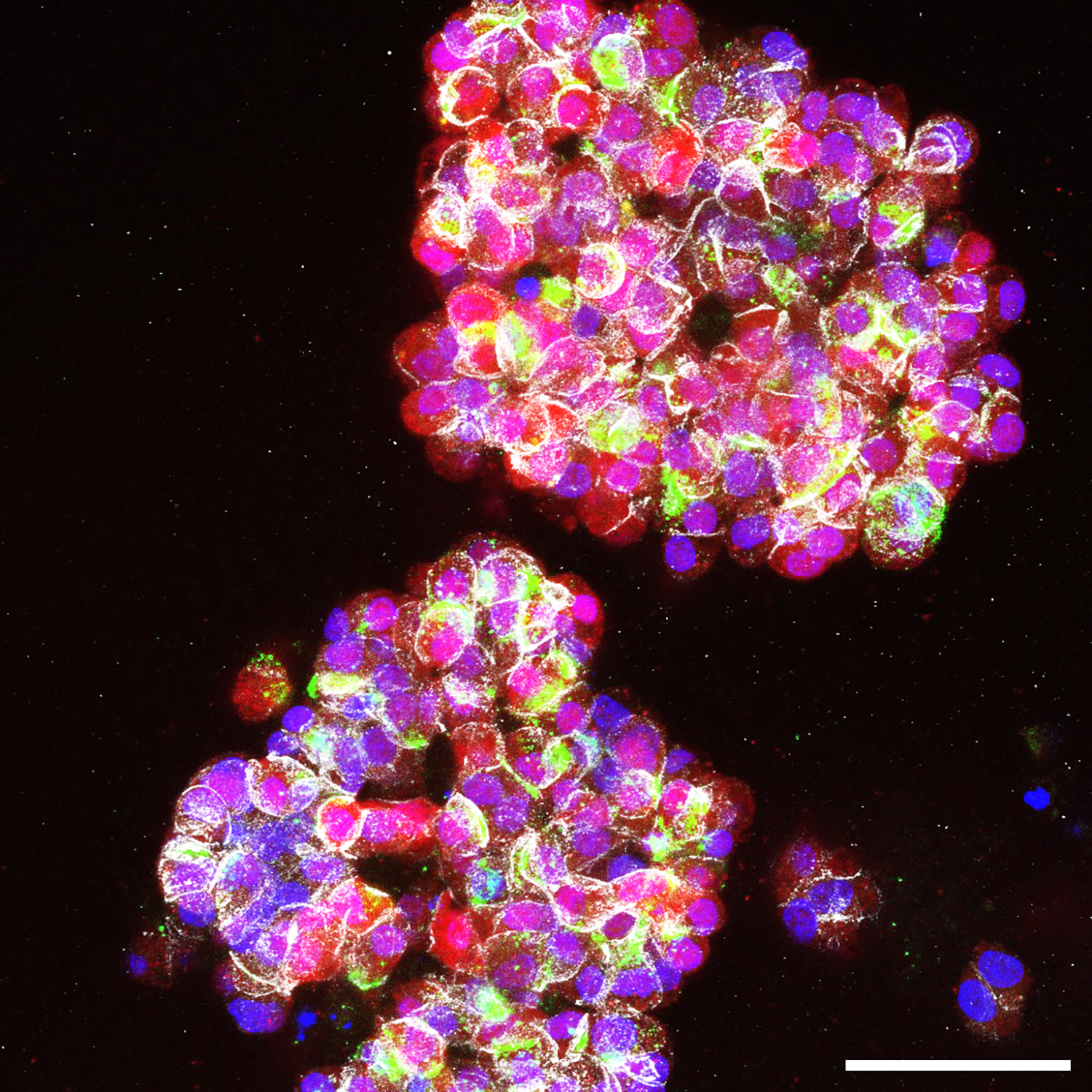

Supplement: Supplementary file 11 — Source data Fig. 1 [file 44318_2024_328_MOESM11_ESM.zip › Figure 1G. HT2-280-HOPX-Ecad_3.jpg]

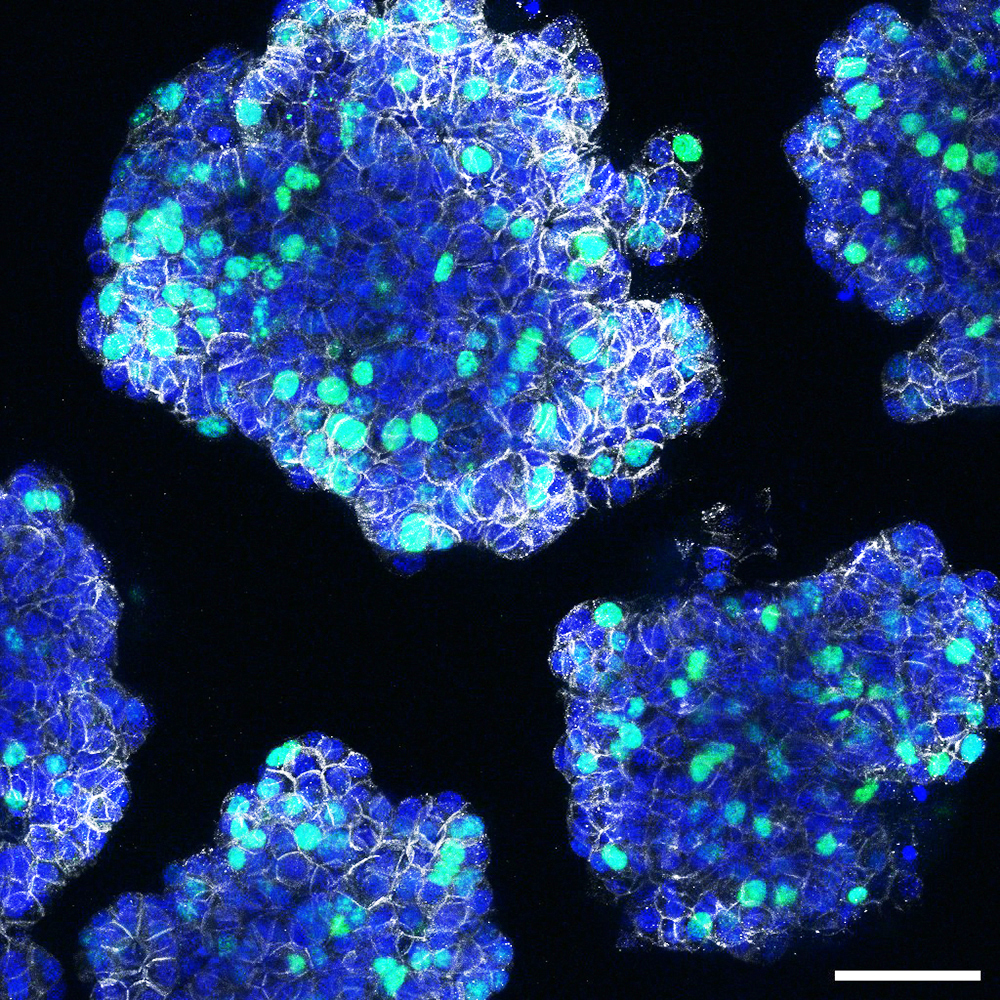

Supplement: Supplementary file 11 — Source data Fig. 1 [file 44318_2024_328_MOESM11_ESM.zip › Figure 1G. LAMP3-Ecad-Ki67_1.jpg]

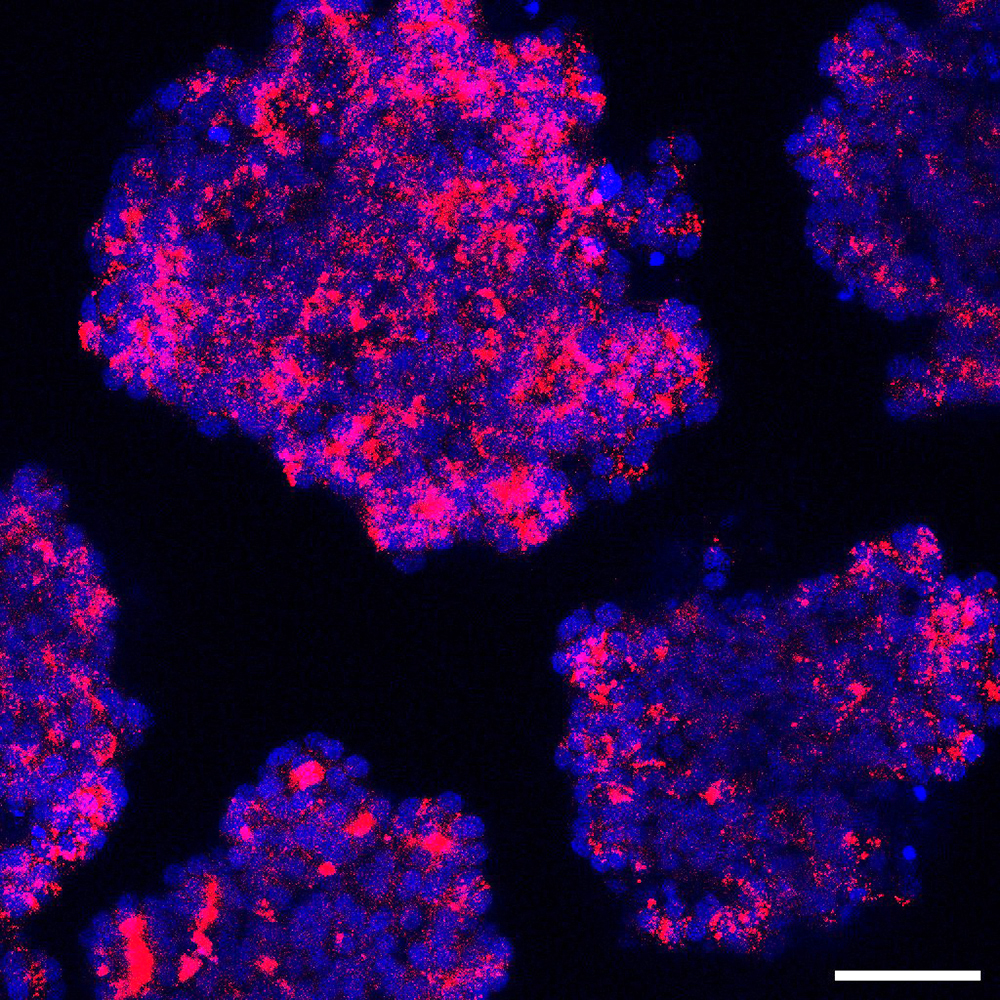

Supplement: Supplementary file 11 — Source data Fig. 1 [file 44318_2024_328_MOESM11_ESM.zip › Figure 1G. LAMP3-Ecad-Ki67_2.jpg]

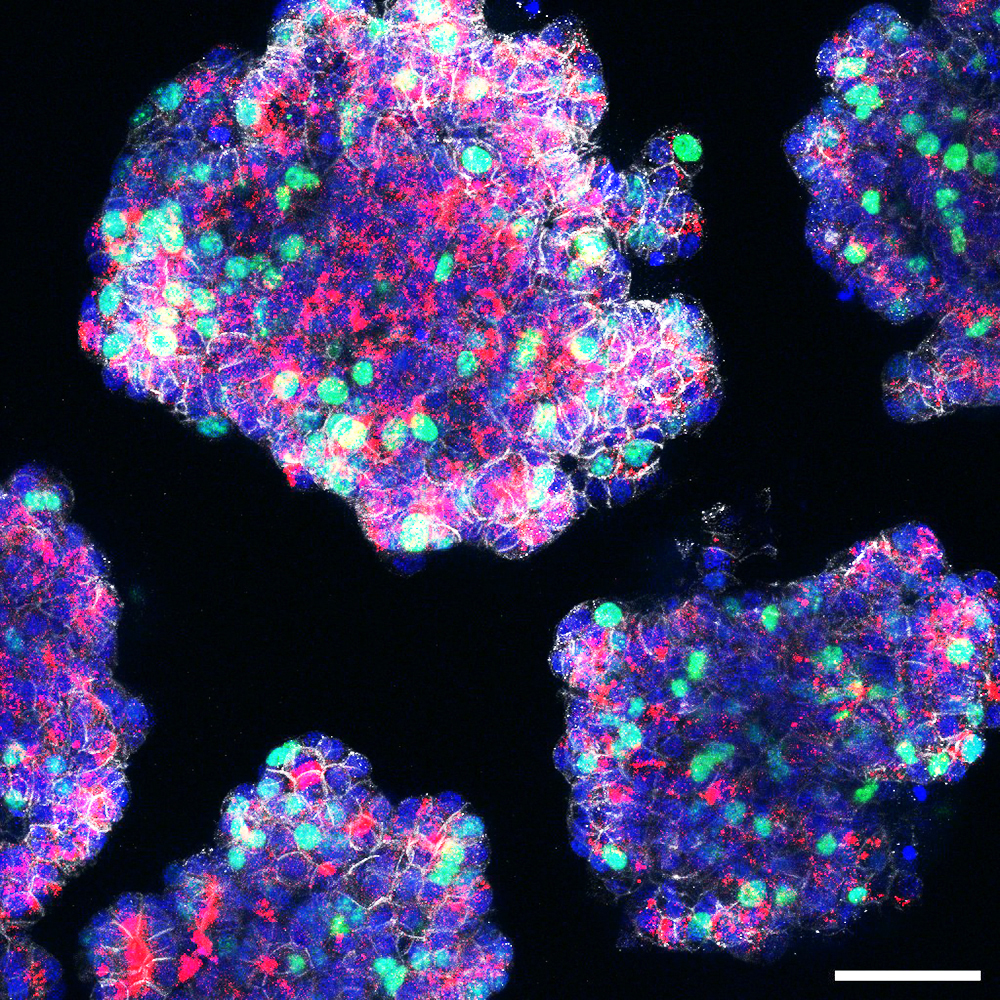

Supplement: Supplementary file 11 — Source data Fig. 1 [file 44318_2024_328_MOESM11_ESM.zip › Figure 1G. LAMP3-Ecad-Ki67_3.jpg]

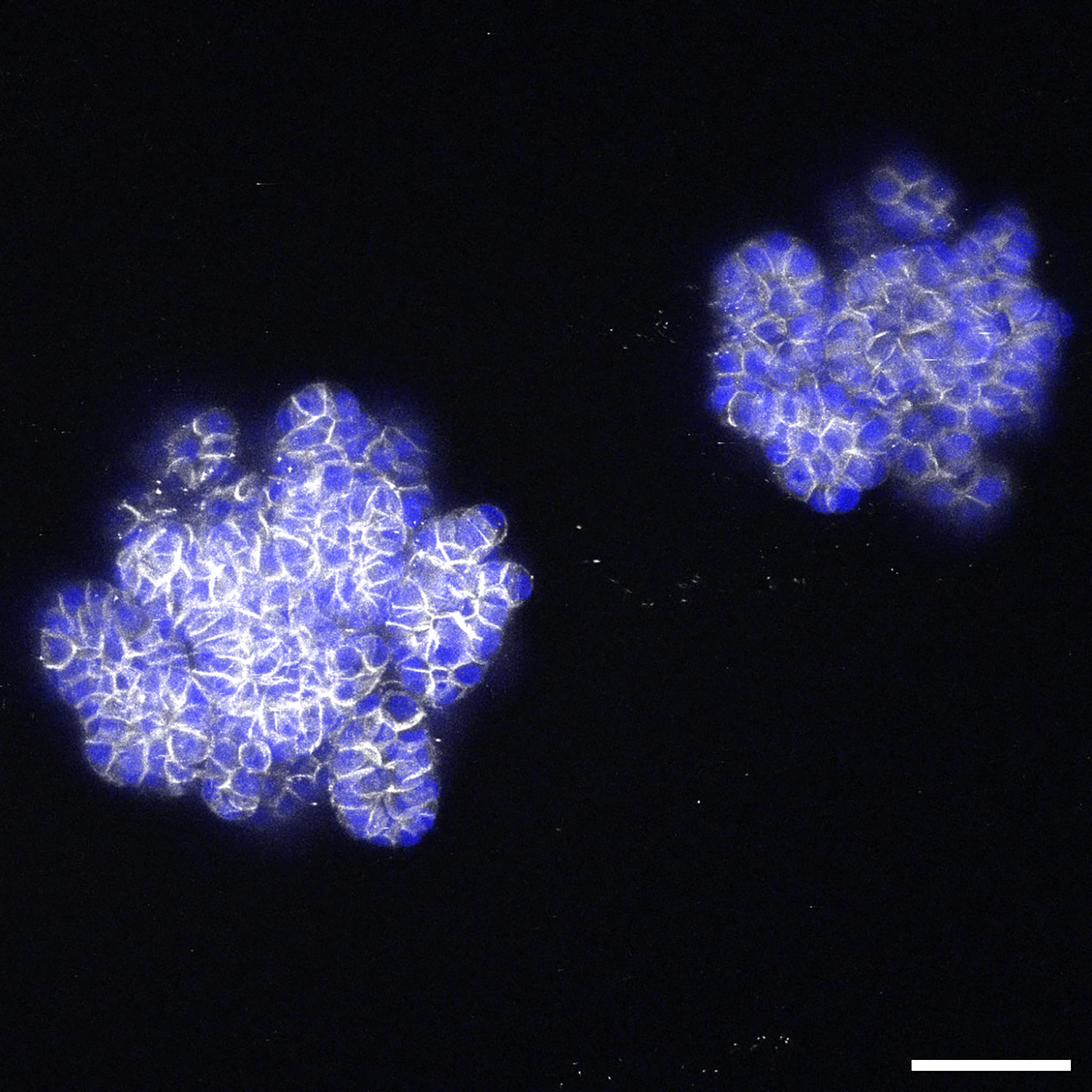

Supplement: Supplementary file 11 — Source data Fig. 1 [file 44318_2024_328_MOESM11_ESM.zip › Figure 1G. Mature SFTPB-Ecad_1.jpg]

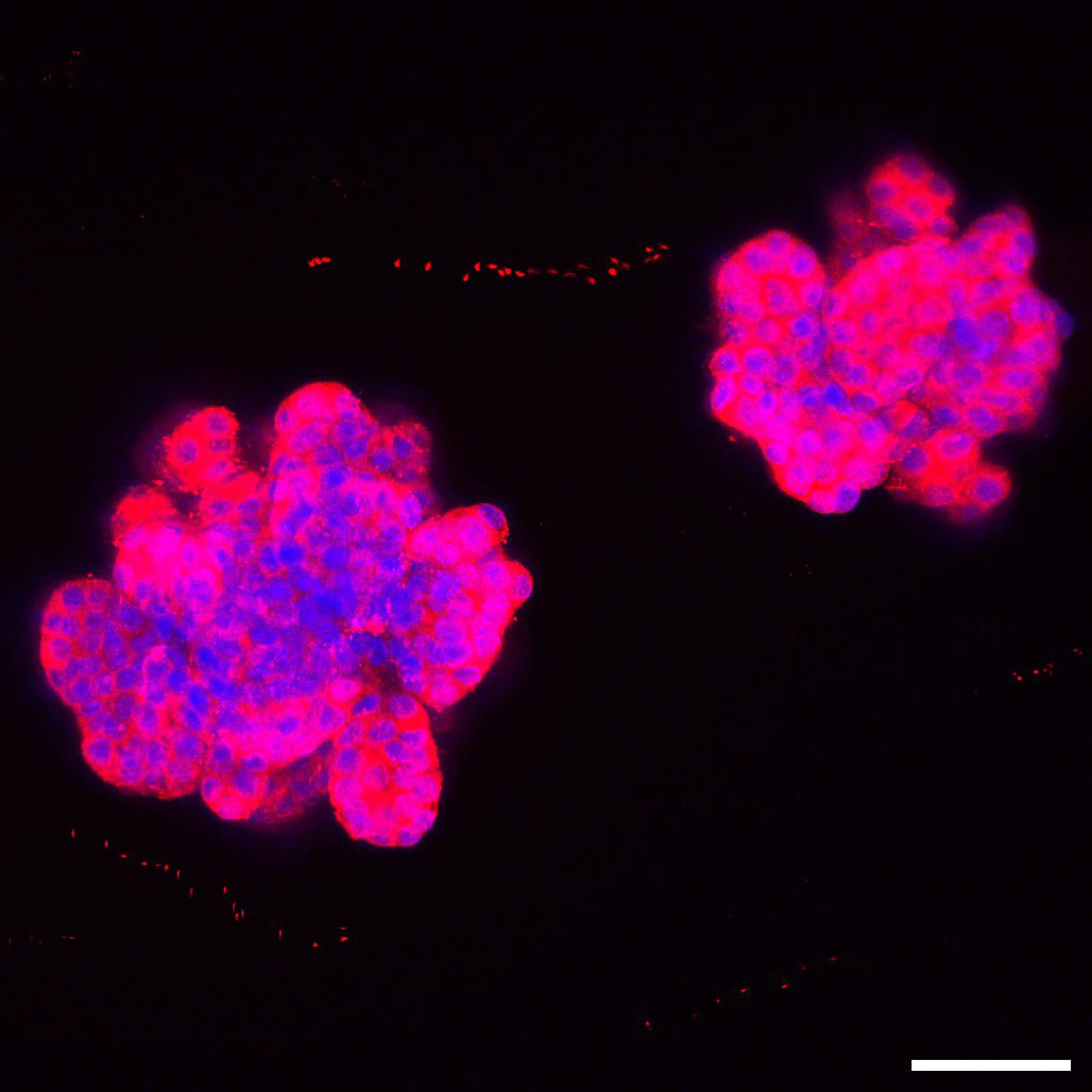

Supplement: Supplementary file 11 — Source data Fig. 1 [file 44318_2024_328_MOESM11_ESM.zip › Figure 1G. Mature SFTPB-Ecad_2.jpg]

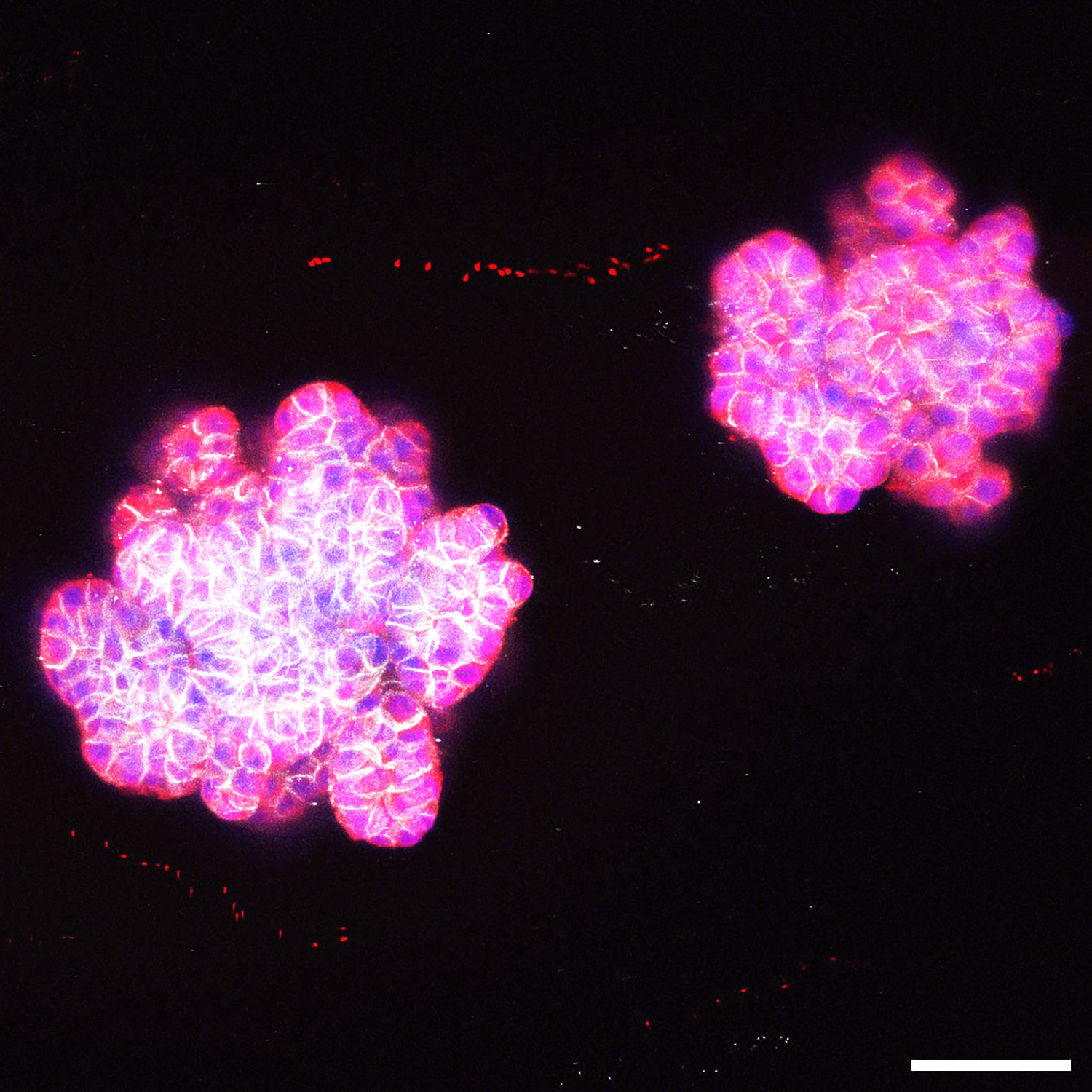

Supplement: Supplementary file 11 — Source data Fig. 1 [file 44318_2024_328_MOESM11_ESM.zip › Figure 1G. Mature SFTPB-Ecad_3.jpg]

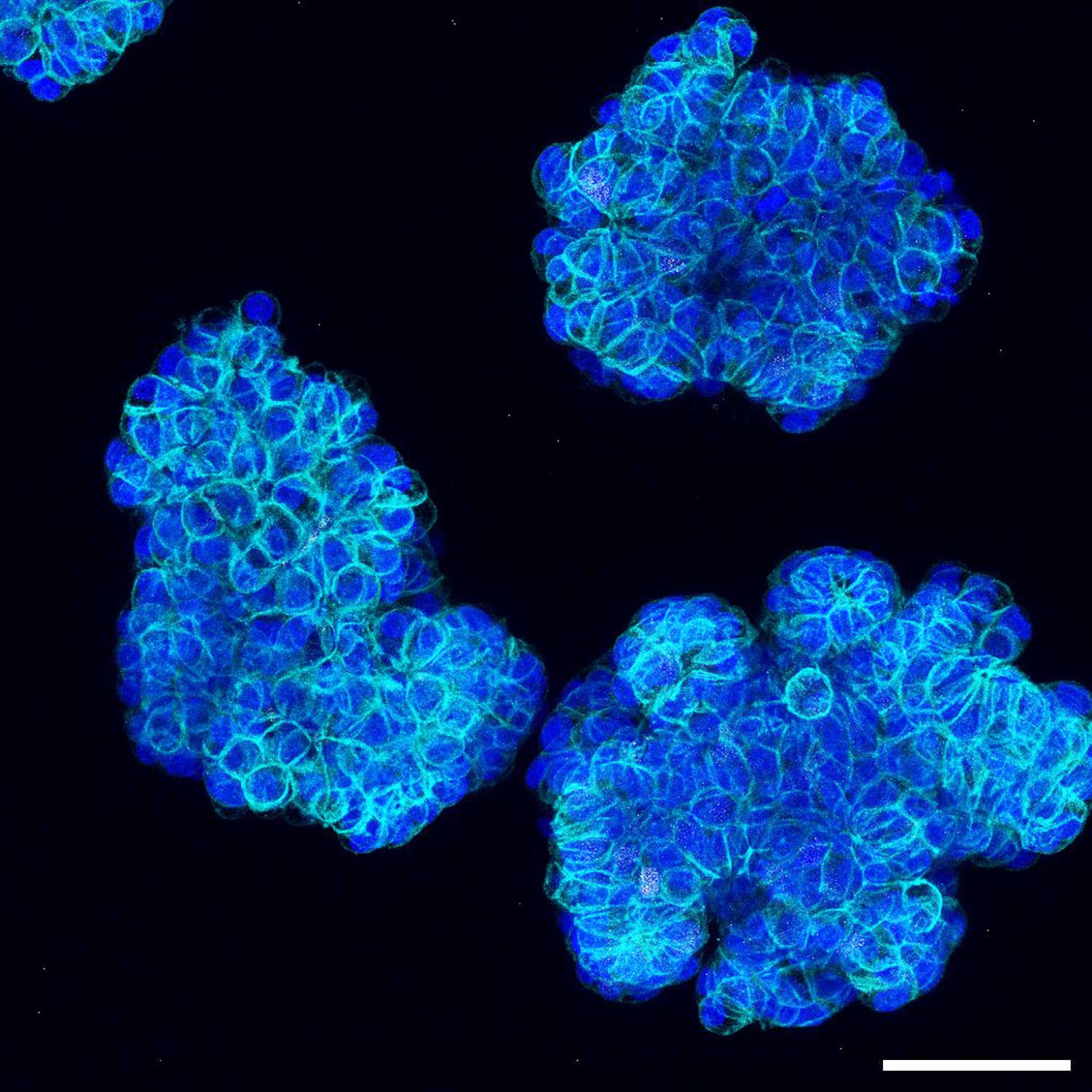

Supplement: Supplementary file 11 — Source data Fig. 1 [file 44318_2024_328_MOESM11_ESM.zip › Figure 1G. Mature SFTPC-b-catenin-SOX9_1.jpg]

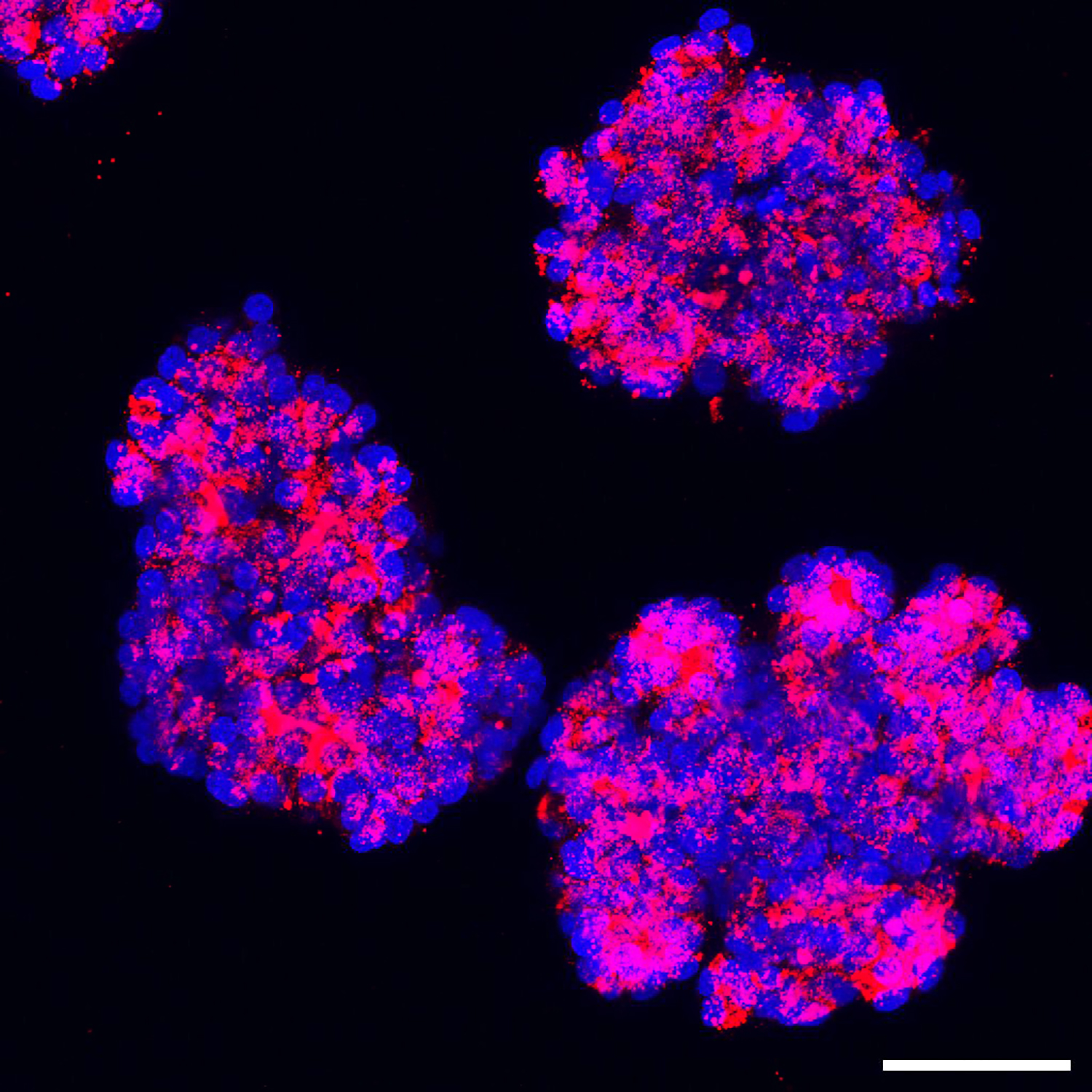

Supplement: Supplementary file 11 — Source data Fig. 1 [file 44318_2024_328_MOESM11_ESM.zip › Figure 1G. Mature SFTPC-b-catenin-SOX9_2.jpg]

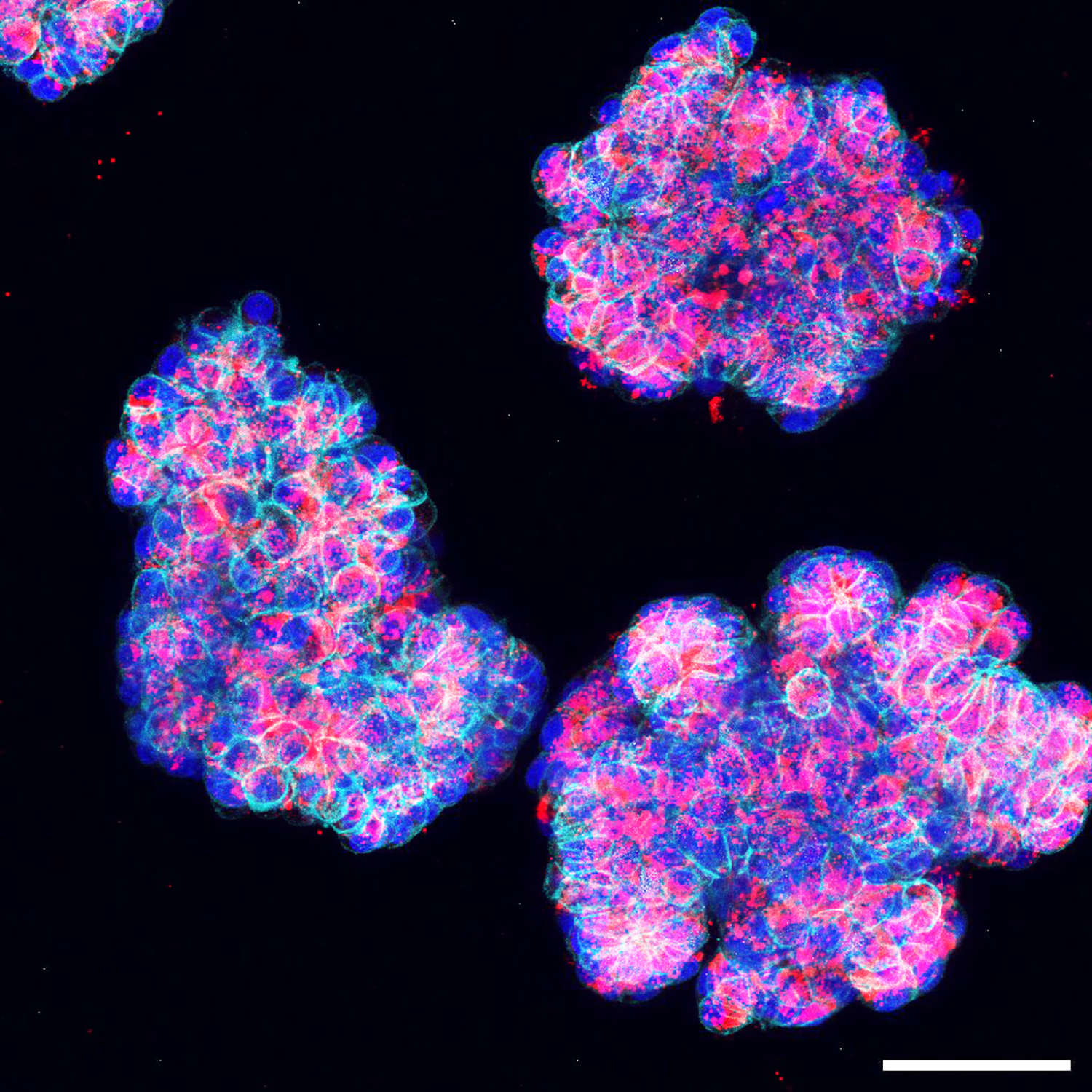

Supplement: Supplementary file 11 — Source data Fig. 1 [file 44318_2024_328_MOESM11_ESM.zip › Figure 1G. Mature SFTPC-b-catenin-SOX9_3.jpg]

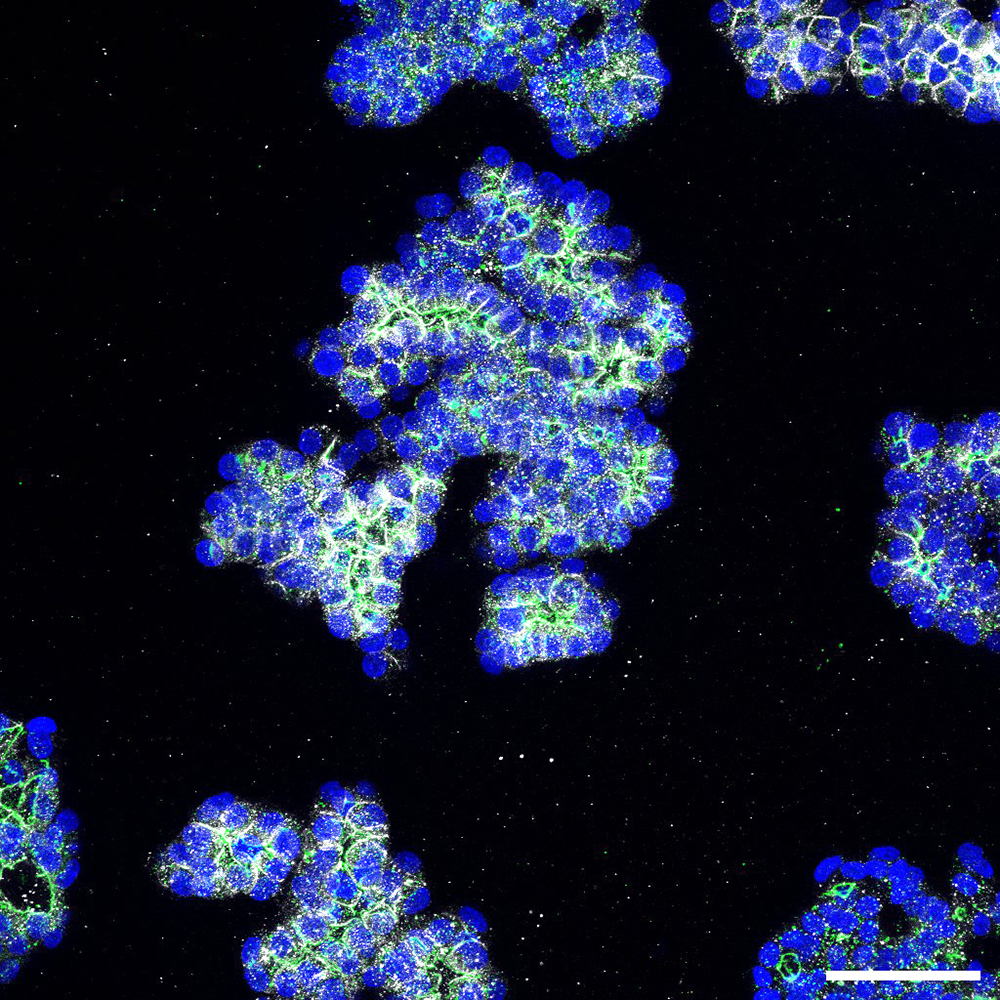

Supplement: Supplementary file 11 — Source data Fig. 1 [file 44318_2024_328_MOESM11_ESM.zip › Figure 1G. NAPSA-ZO1-Ecad_1.jpg]

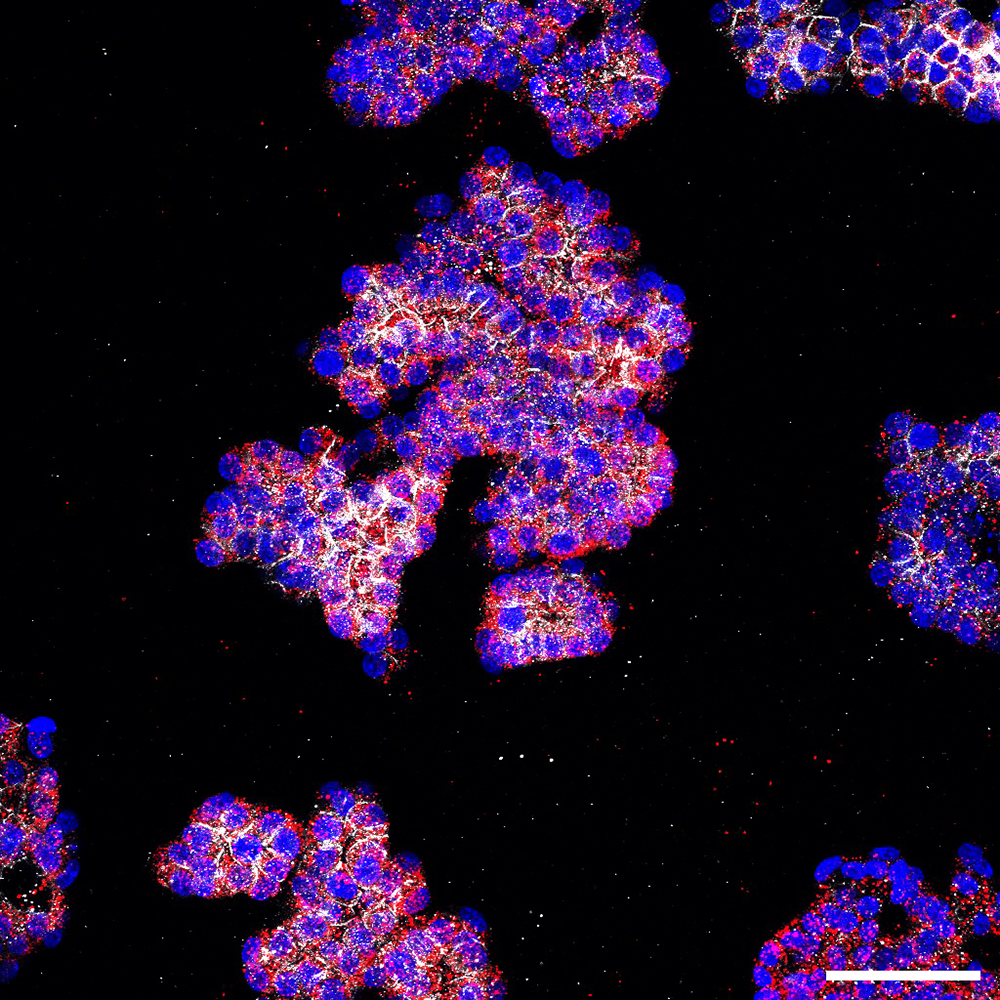

Supplement: Supplementary file 11 — Source data Fig. 1 [file 44318_2024_328_MOESM11_ESM.zip › Figure 1G. NAPSA-ZO1-Ecad_2.jpg]

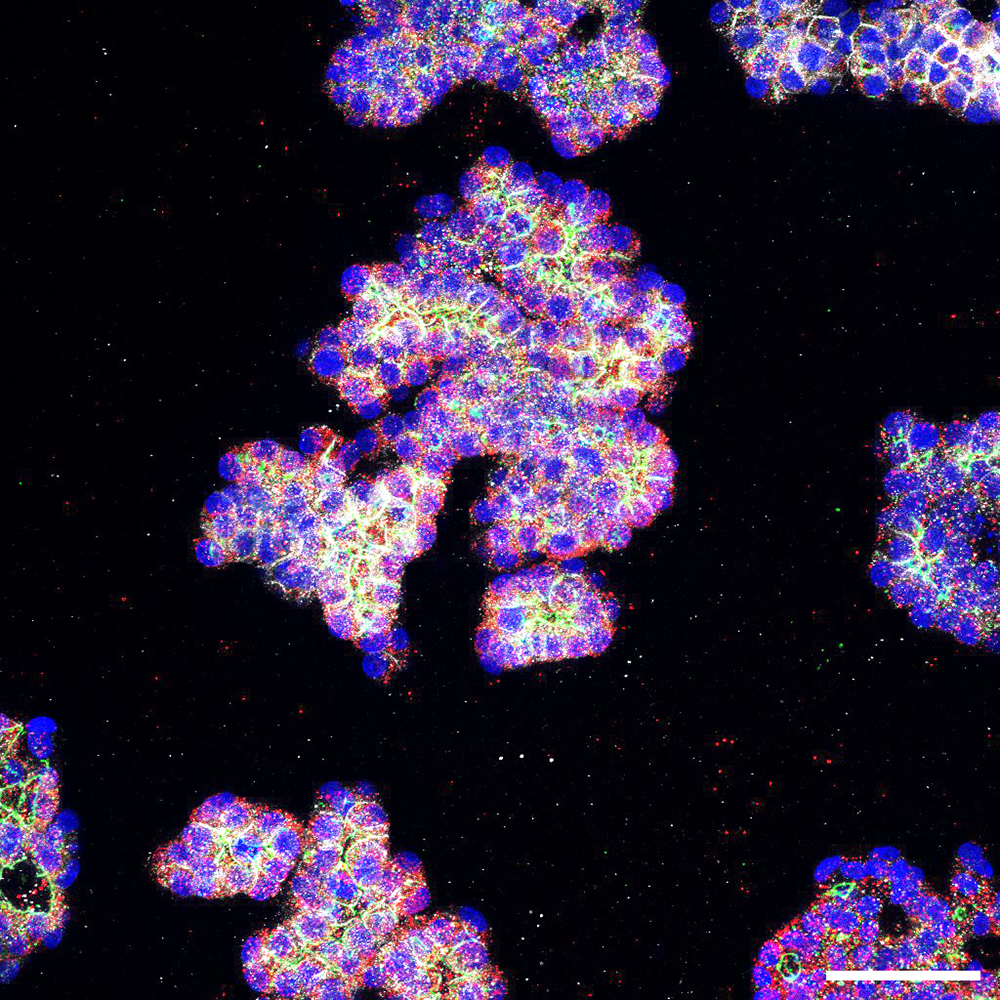

Supplement: Supplementary file 11 — Source data Fig. 1 [file 44318_2024_328_MOESM11_ESM.zip › Figure 1G. NAPSA-ZO1-Ecad_3.jpg]

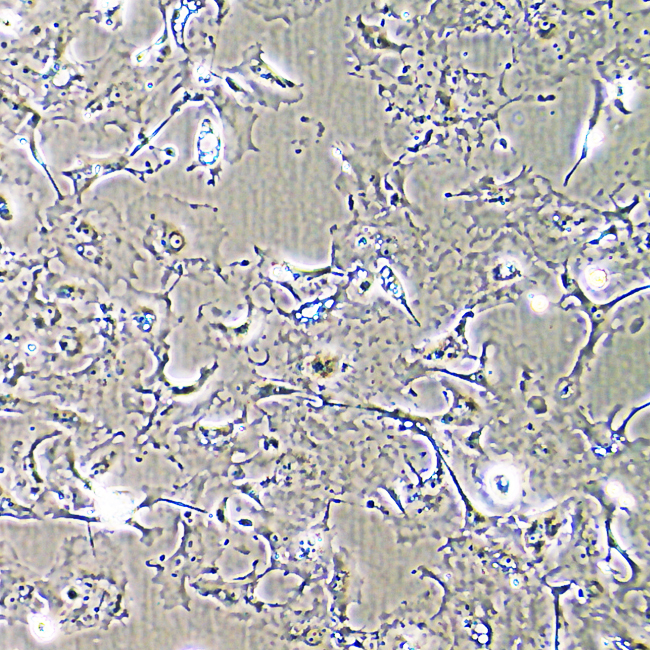

Supplement: Supplementary file 12 — Source data Fig. 2 [file 44318_2024_328_MOESM12_ESM.zip › Figure 2B. AT1-like on 2D_magnification.jpg]

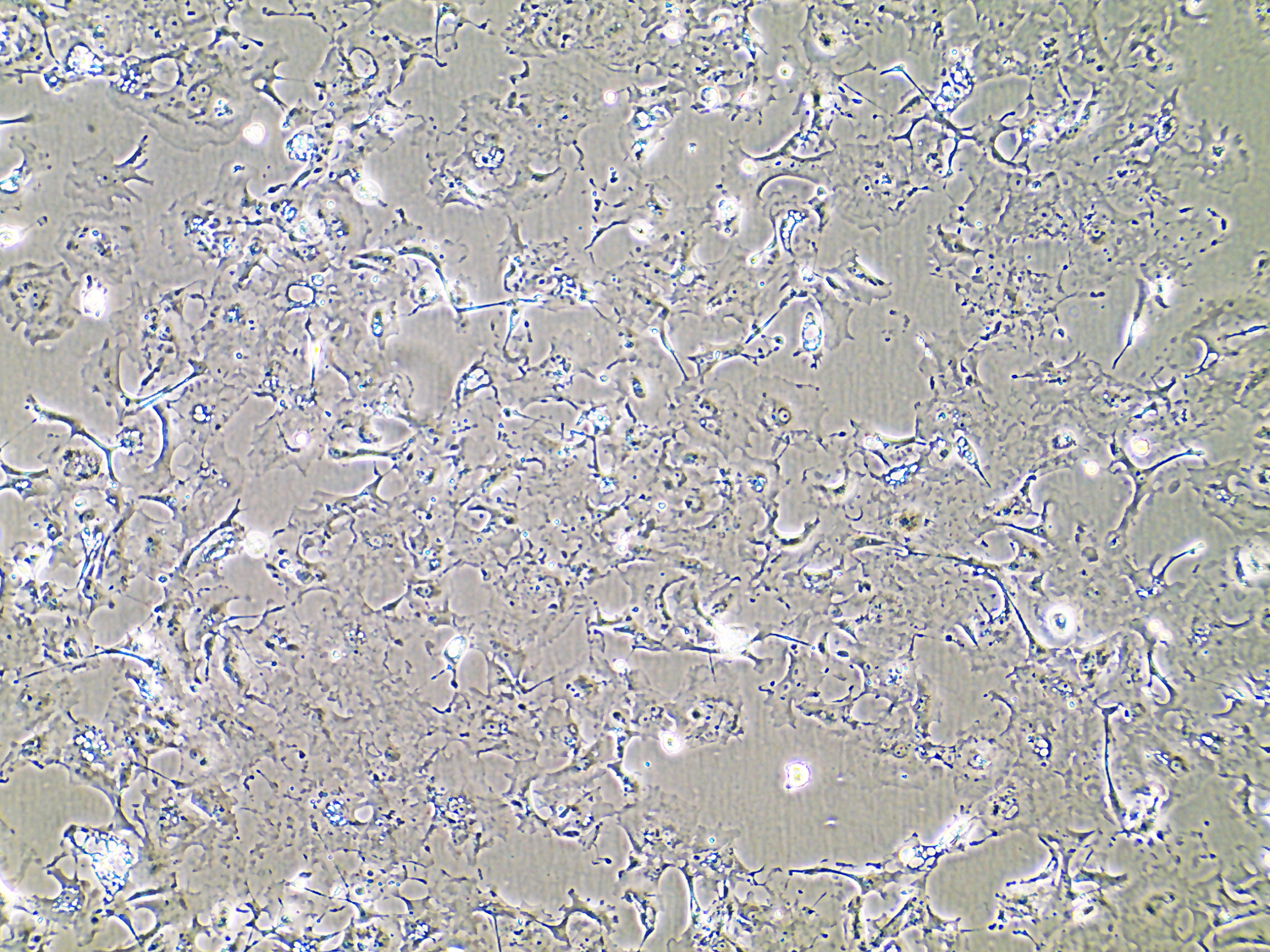

Supplement: Supplementary file 12 — Source data Fig. 2 [file 44318_2024_328_MOESM12_ESM.zip › Figure 2B. AT1-like on 2D.jpg]

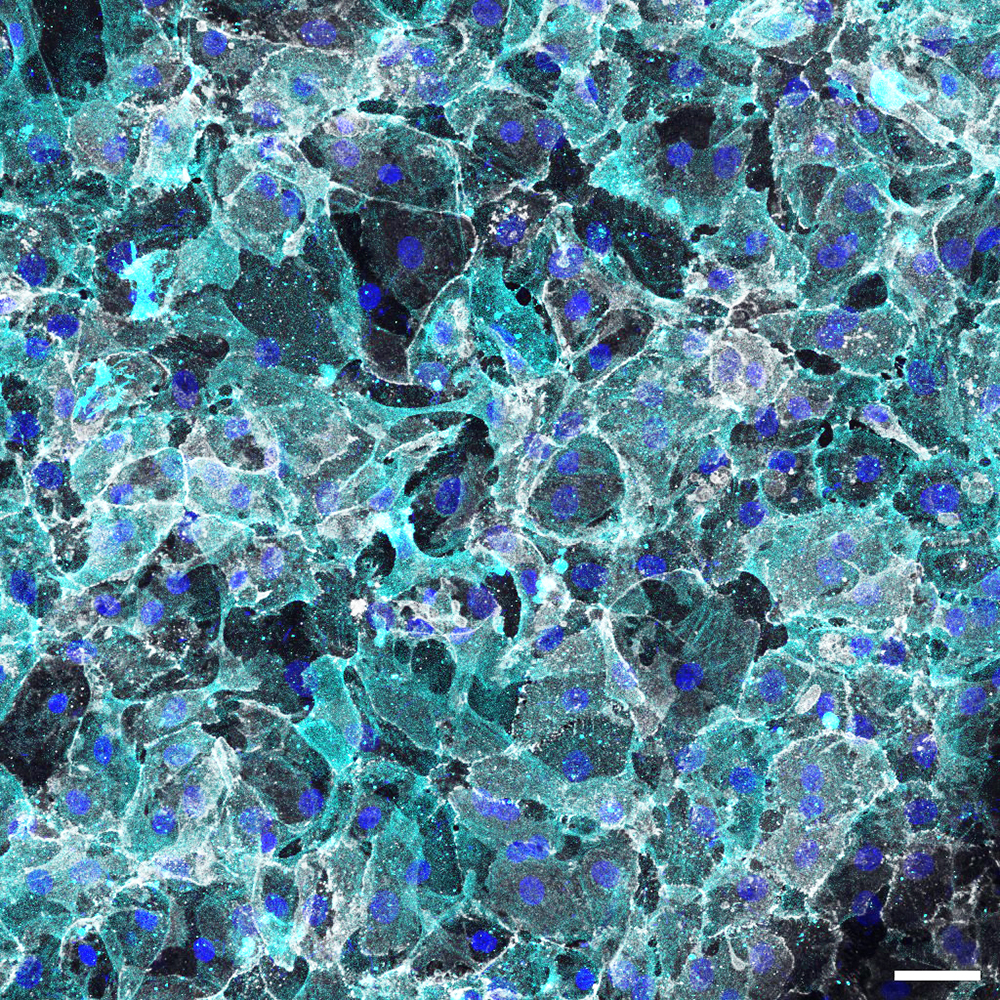

Supplement: Supplementary file 12 — Source data Fig. 2 [file 44318_2024_328_MOESM12_ESM.zip › Figure 2D. CAV1-b-ACTIN-E-cad_Ecad b-actin.jpg]

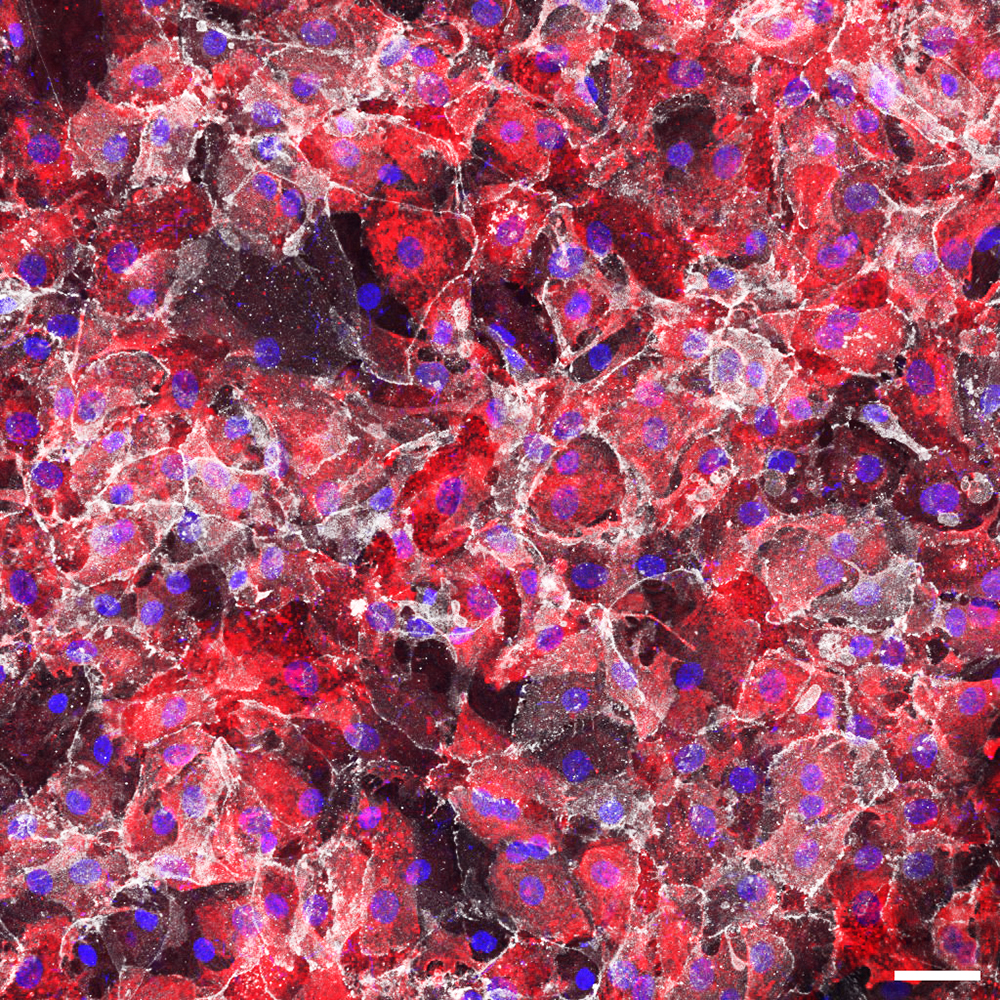

Supplement: Supplementary file 12 — Source data Fig. 2 [file 44318_2024_328_MOESM12_ESM.zip › Figure 2D. CAV1-b-ACTIN-E-cad_Ecad CAV1.jpg]

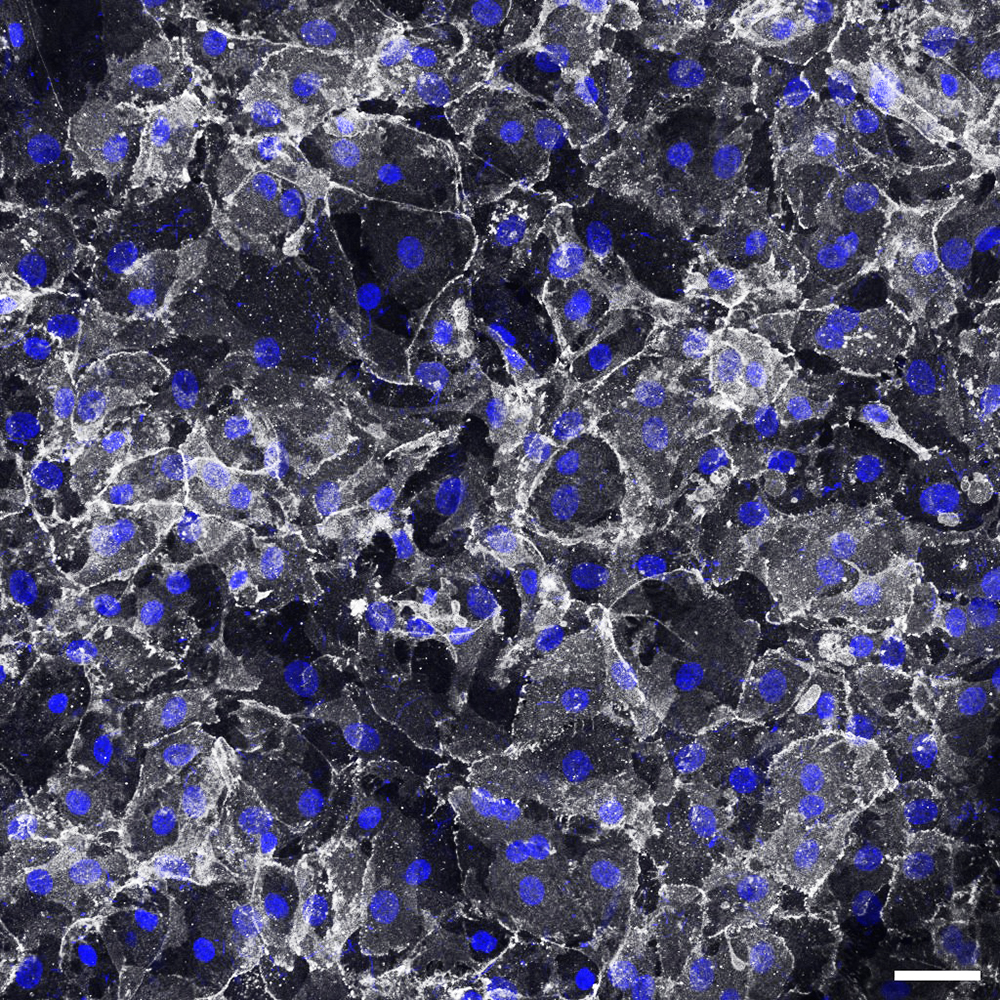

Supplement: Supplementary file 12 — Source data Fig. 2 [file 44318_2024_328_MOESM12_ESM.zip › Figure 2D. CAV1-b-ACTIN-E-cad_Ecad DAPI.jpg]

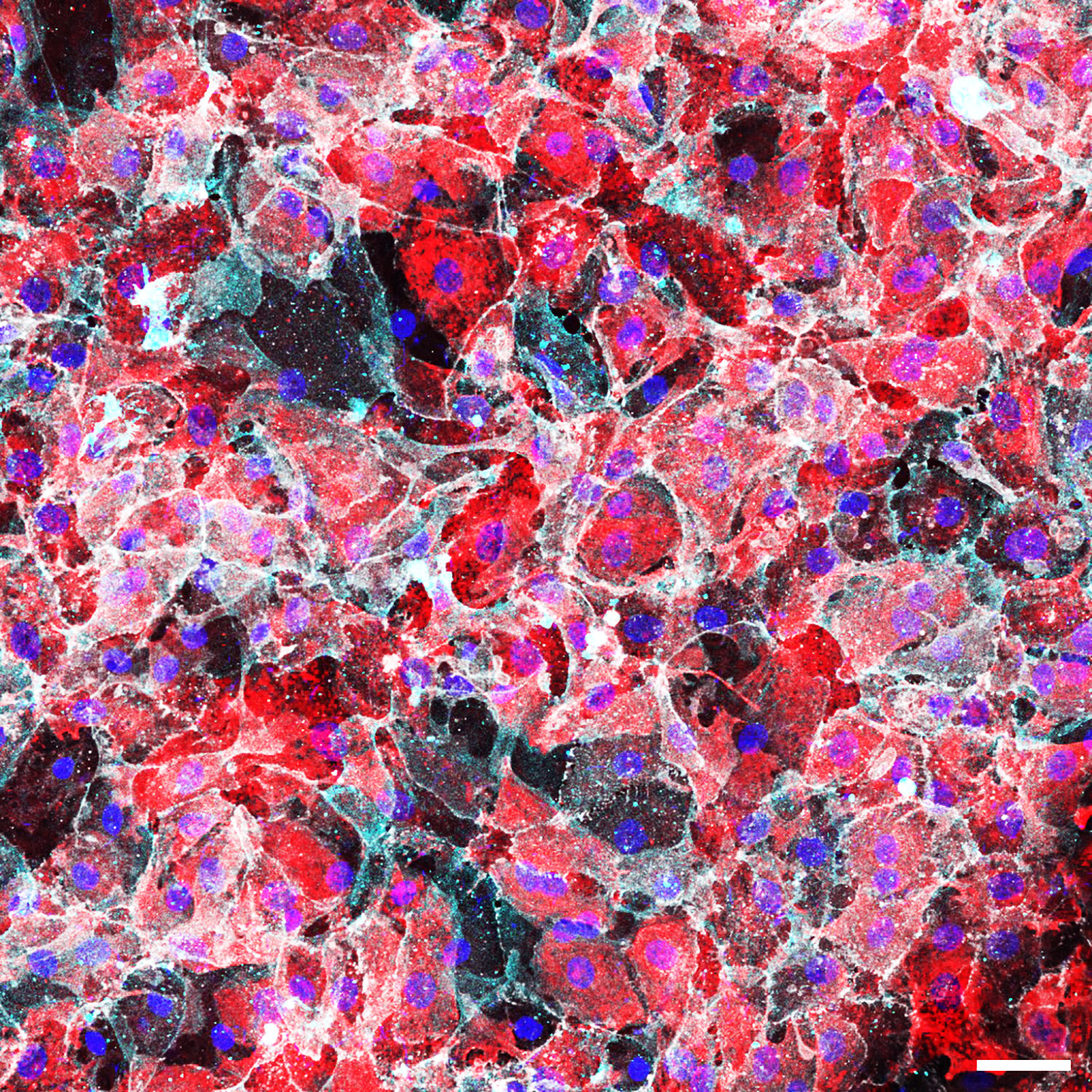

Supplement: Supplementary file 12 — Source data Fig. 2 [file 44318_2024_328_MOESM12_ESM.zip › Figure 2D. CAV1-b-ACTIN-E-cad_merged.jpg]

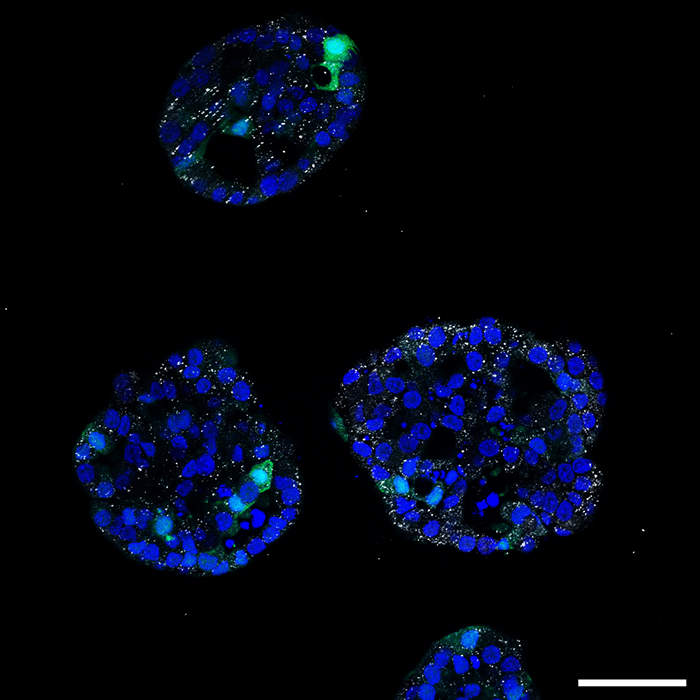

Supplement: Supplementary file 12 — Source data Fig. 2 [file 44318_2024_328_MOESM12_ESM.zip › Figure 2G. Control_AGER GFP.jpg]

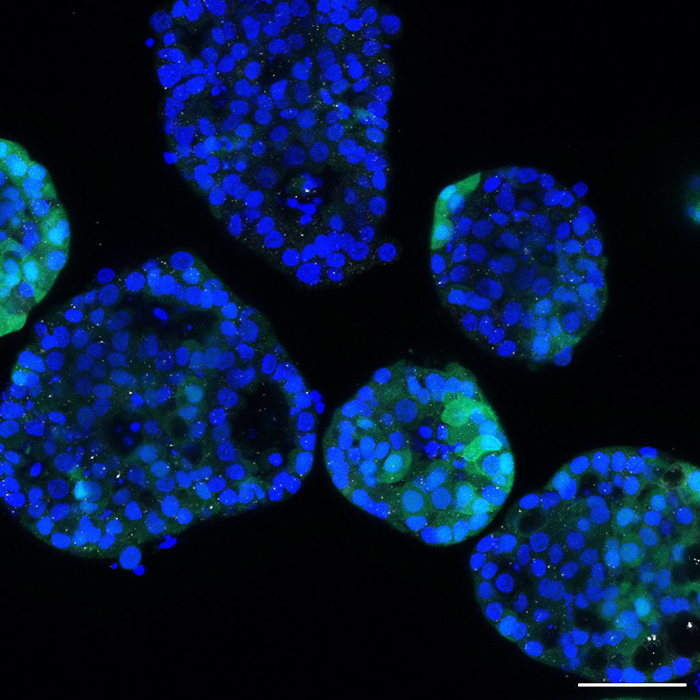

Supplement: Supplementary file 12 — Source data Fig. 2 [file 44318_2024_328_MOESM12_ESM.zip › Figure 2G. Control_CAV1 GFP.jpg]

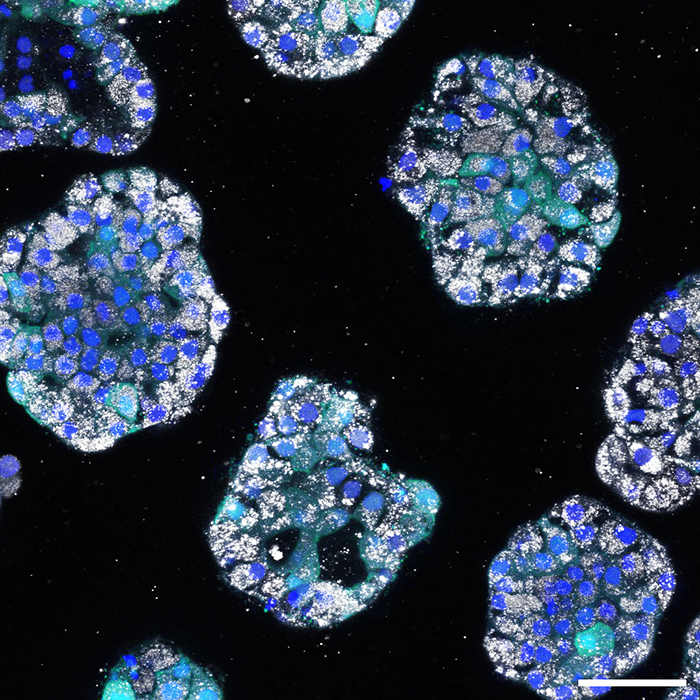

Supplement: Supplementary file 12 — Source data Fig. 2 [file 44318_2024_328_MOESM12_ESM.zip › Figure 2G. LATS-IN_AGER GFP.jpg]

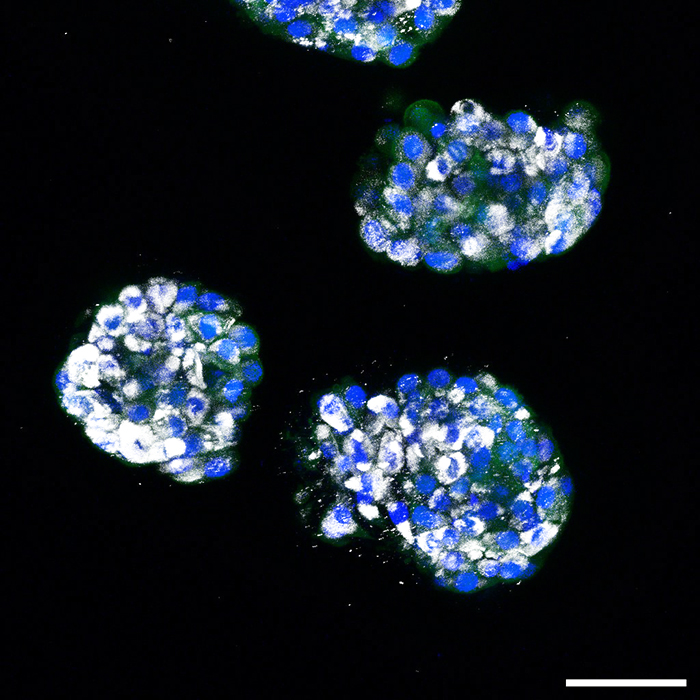

Supplement: Supplementary file 12 — Source data Fig. 2 [file 44318_2024_328_MOESM12_ESM.zip › Figure 2G. LATS-IN_CAV1 GFP.jpg]

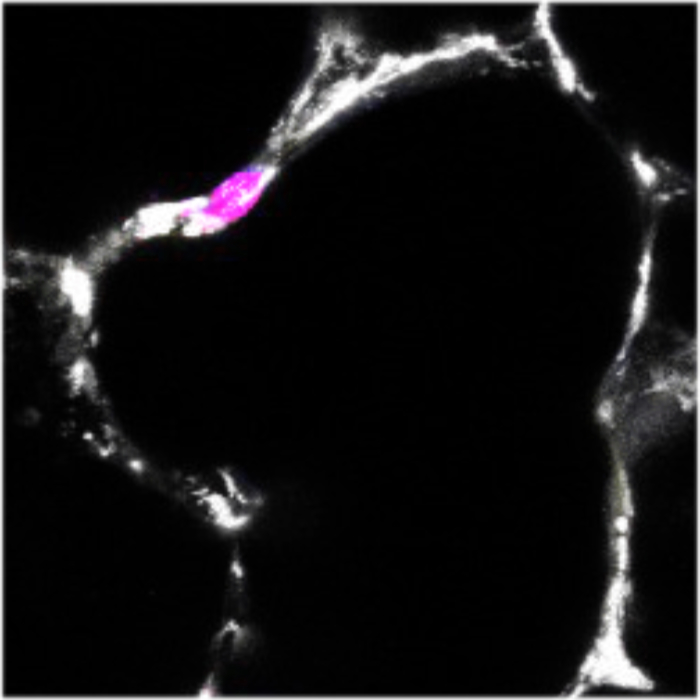

Supplement: Supplementary file 12 — Source data Fig. 2 [file 44318_2024_328_MOESM12_ESM.zip › Figure 2I. Explant_SFTPC-CAV1_1_1.jpg]

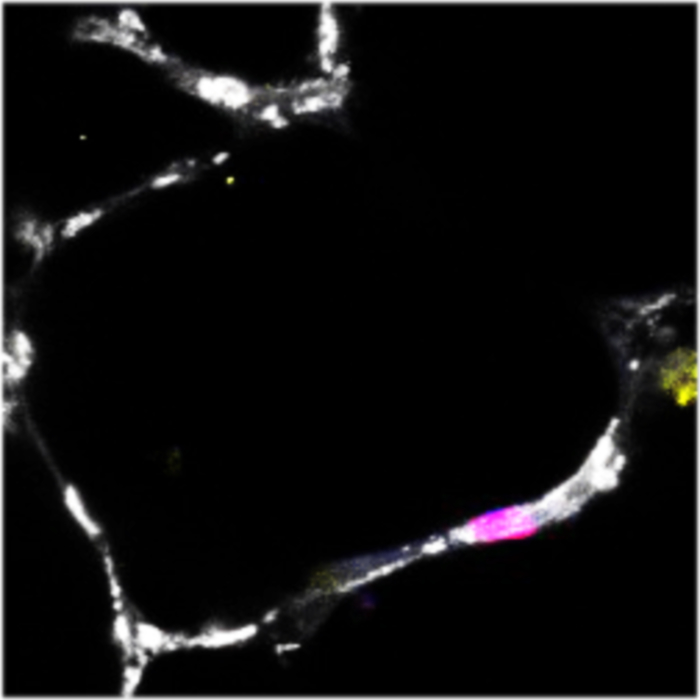

Supplement: Supplementary file 12 — Source data Fig. 2 [file 44318_2024_328_MOESM12_ESM.zip › Figure 2I. Explant_SFTPC-CAV1_1_2.jpg]

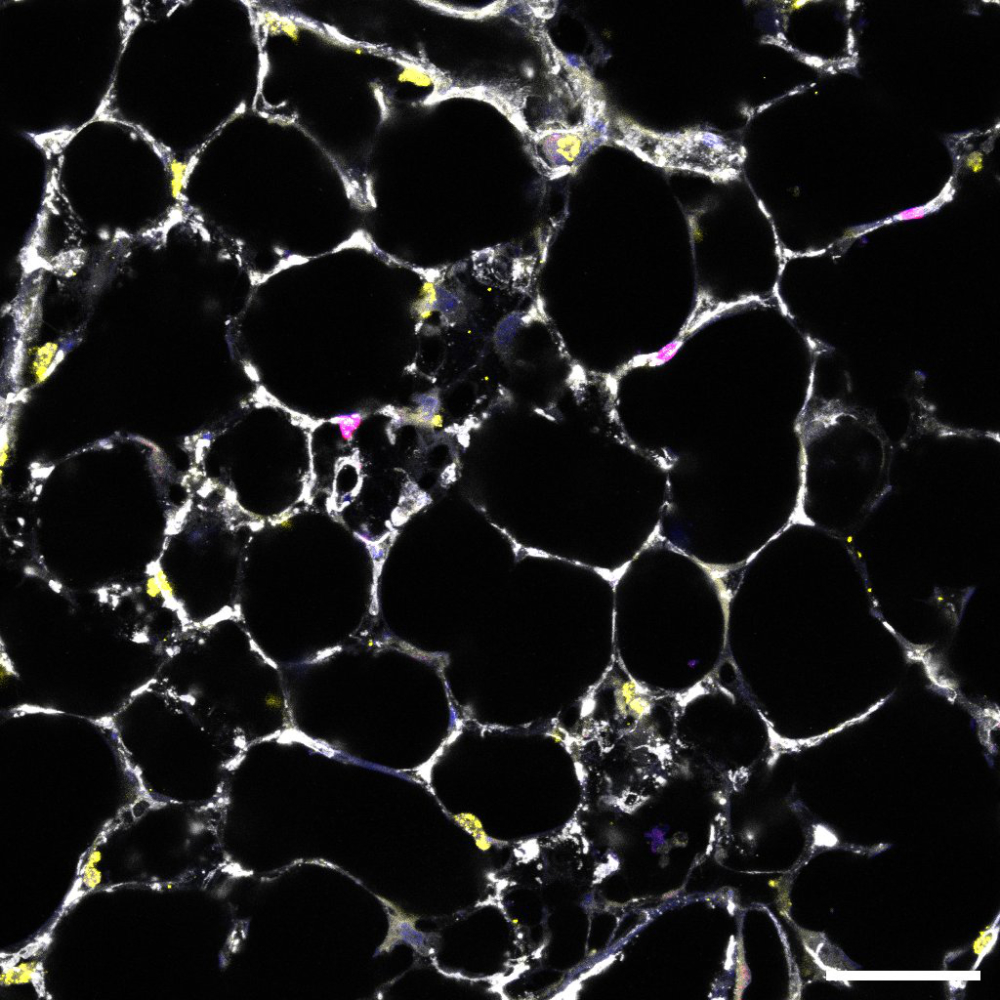

Supplement: Supplementary file 12 — Source data Fig. 2 [file 44318_2024_328_MOESM12_ESM.zip › Figure 2I. Explant_SFTPC-CAV1_1.jpg]

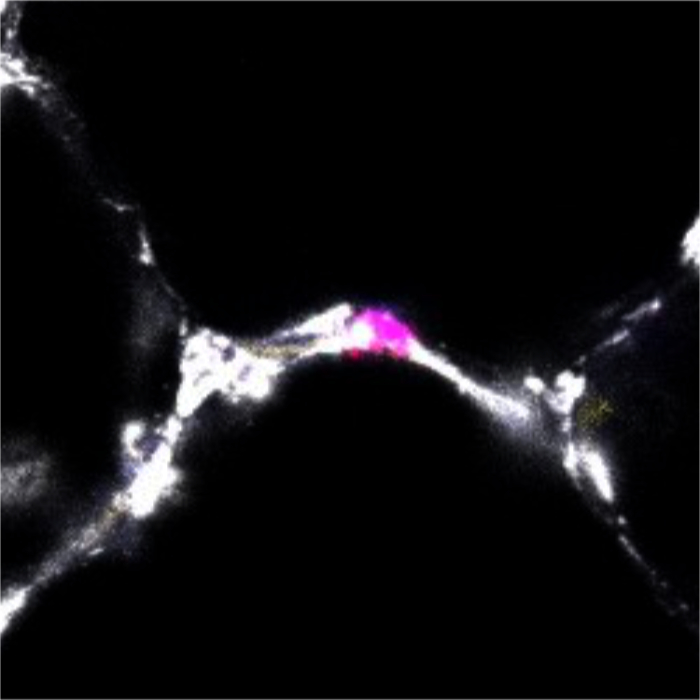

Supplement: Supplementary file 12 — Source data Fig. 2 [file 44318_2024_328_MOESM12_ESM.zip › Figure 2I. Explant_SFTPC-CAV1_2_1.jpg]

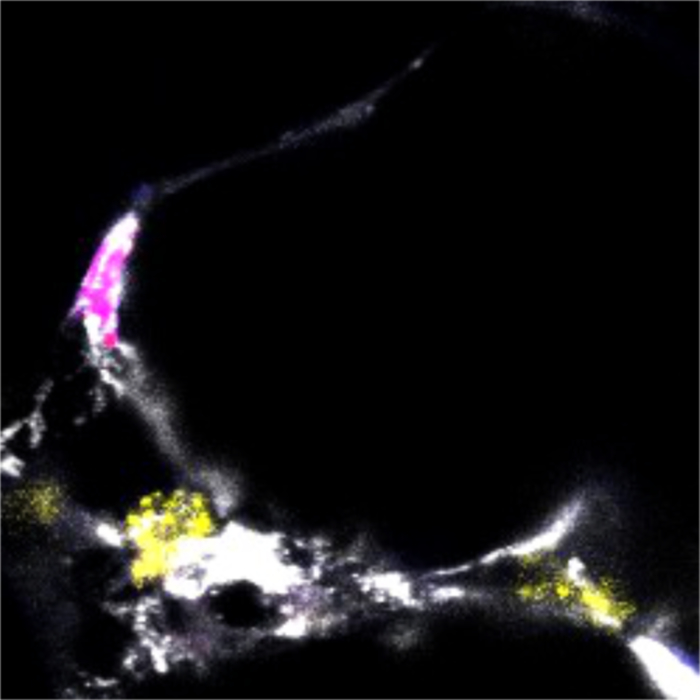

Supplement: Supplementary file 12 — Source data Fig. 2 [file 44318_2024_328_MOESM12_ESM.zip › Figure 2I. Explant_SFTPC-CAV1_2_2.jpg]

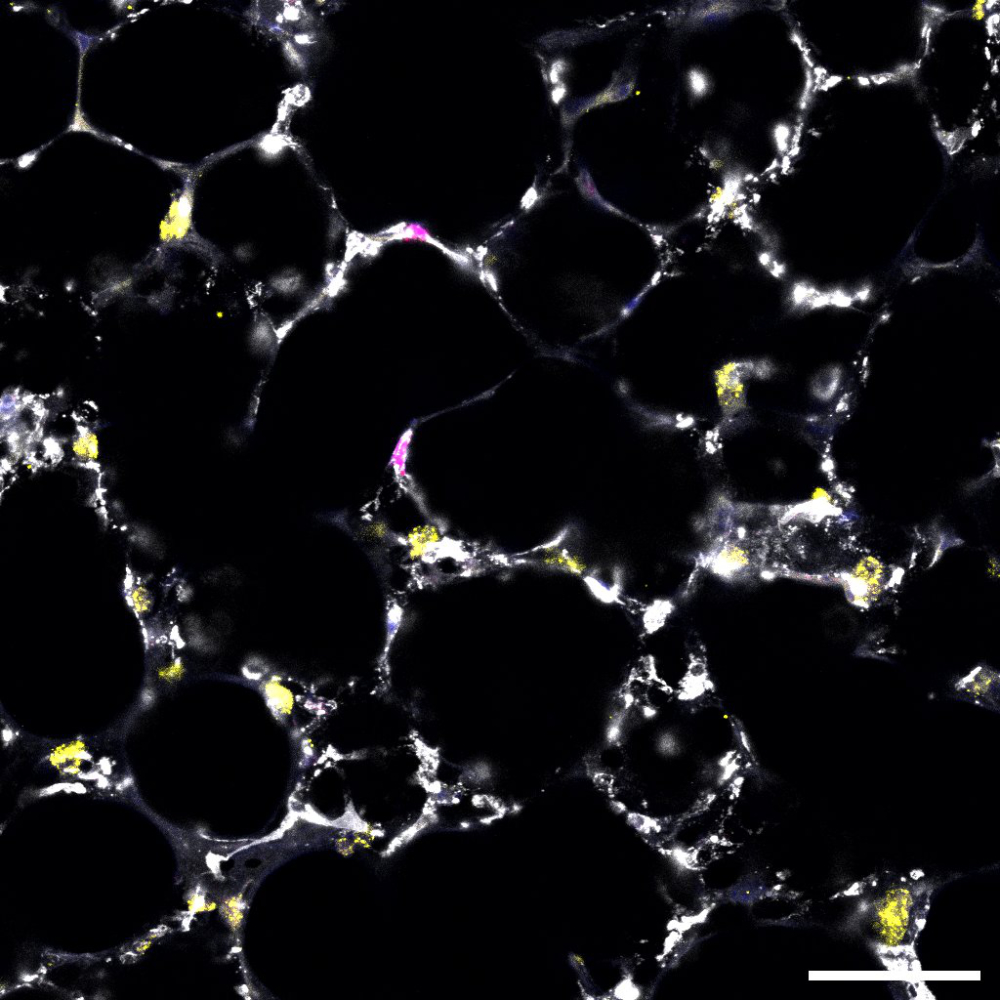

Supplement: Supplementary file 12 — Source data Fig. 2 [file 44318_2024_328_MOESM12_ESM.zip › Figure 2I. Explant_SFTPC-CAV1_2.jpg]

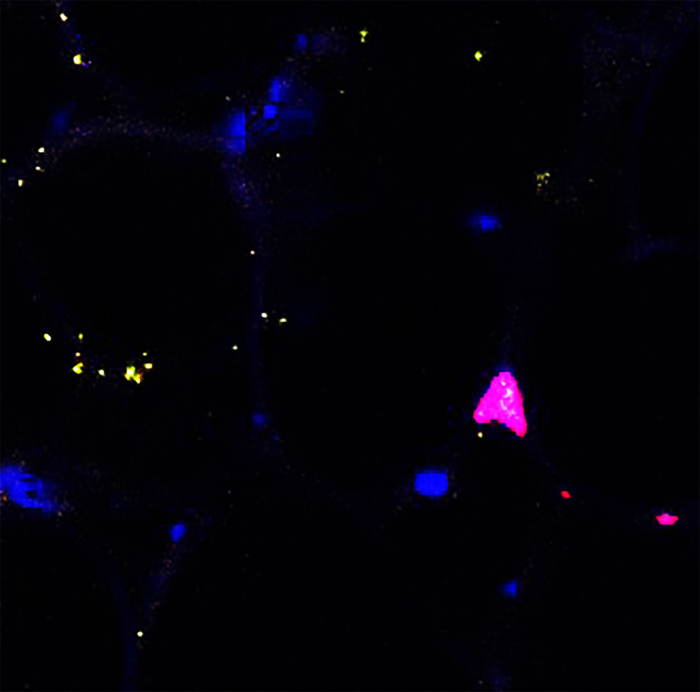

Supplement: Supplementary file 12 — Source data Fig. 2 [file 44318_2024_328_MOESM12_ESM.zip › Figure 2J. Explant_SFTPC-AGER-1-1.jpg]

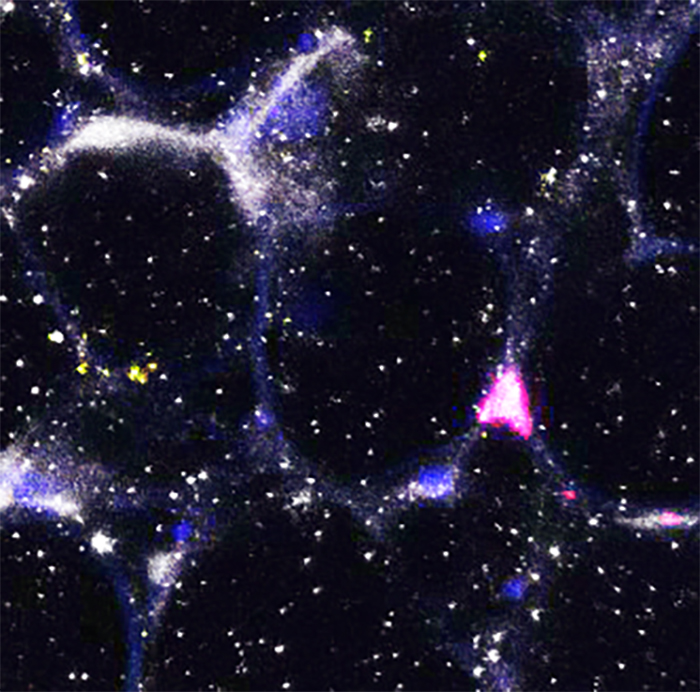

Supplement: Supplementary file 12 — Source data Fig. 2 [file 44318_2024_328_MOESM12_ESM.zip › Figure 2J. Explant_SFTPC-AGER-1-2.jpg]

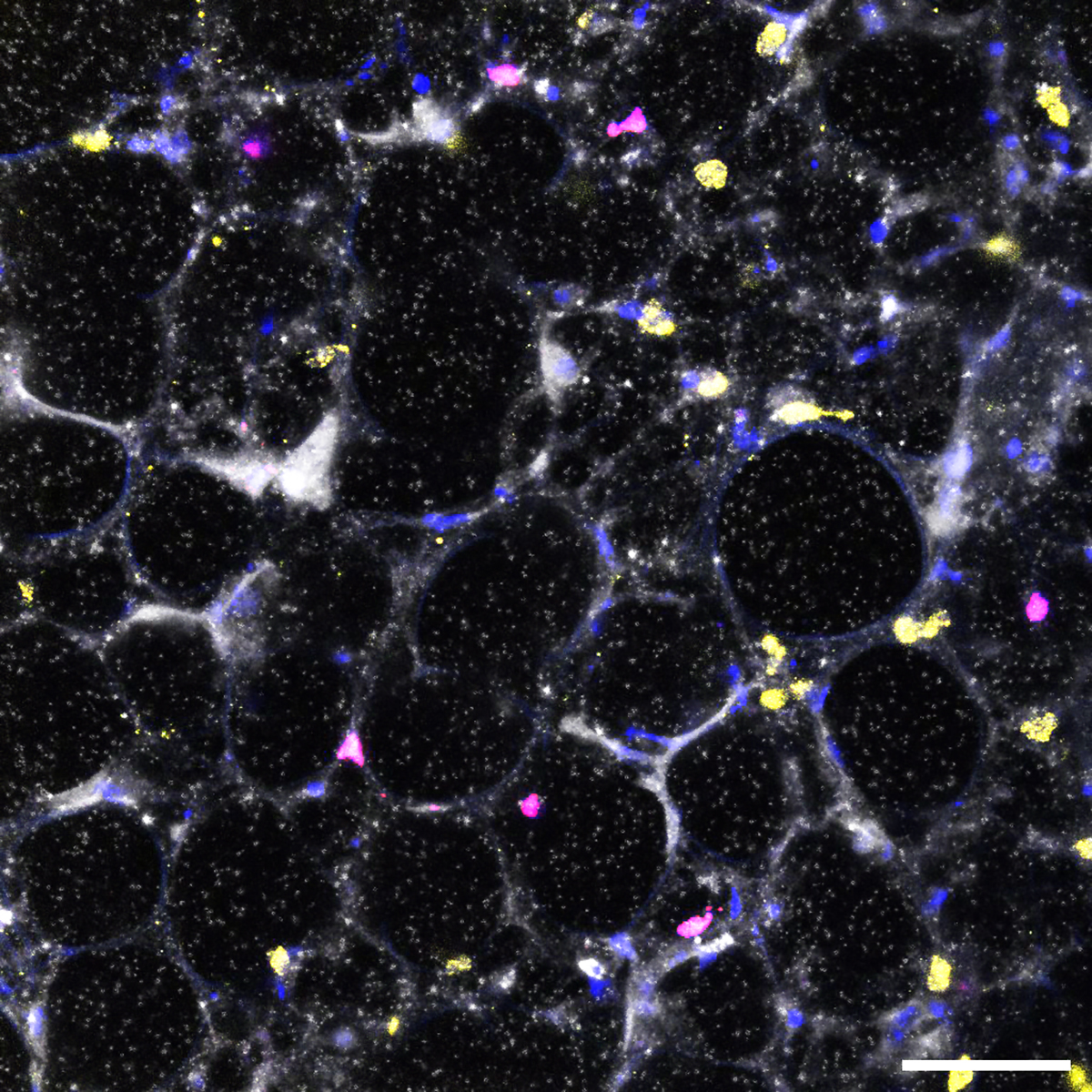

Supplement: Supplementary file 12 — Source data Fig. 2 [file 44318_2024_328_MOESM12_ESM.zip › Figure 2J. Explant_SFTPC-AGER-1.jpg]

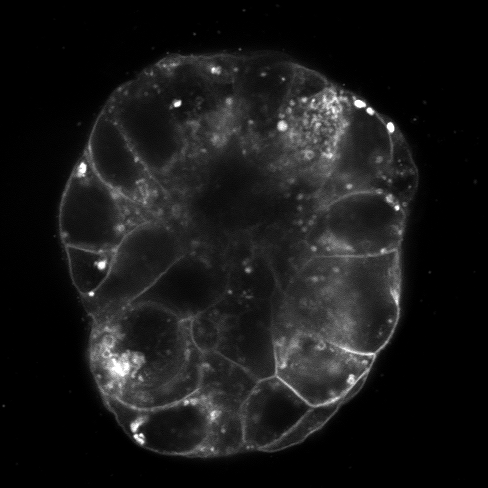

Supplement: Supplementary file 13 — Source data Fig. 3 [file 44318_2024_328_MOESM13_ESM.zip › Figure 3A_ C1-I73T day 10 11_Airyscan Processing.tif]

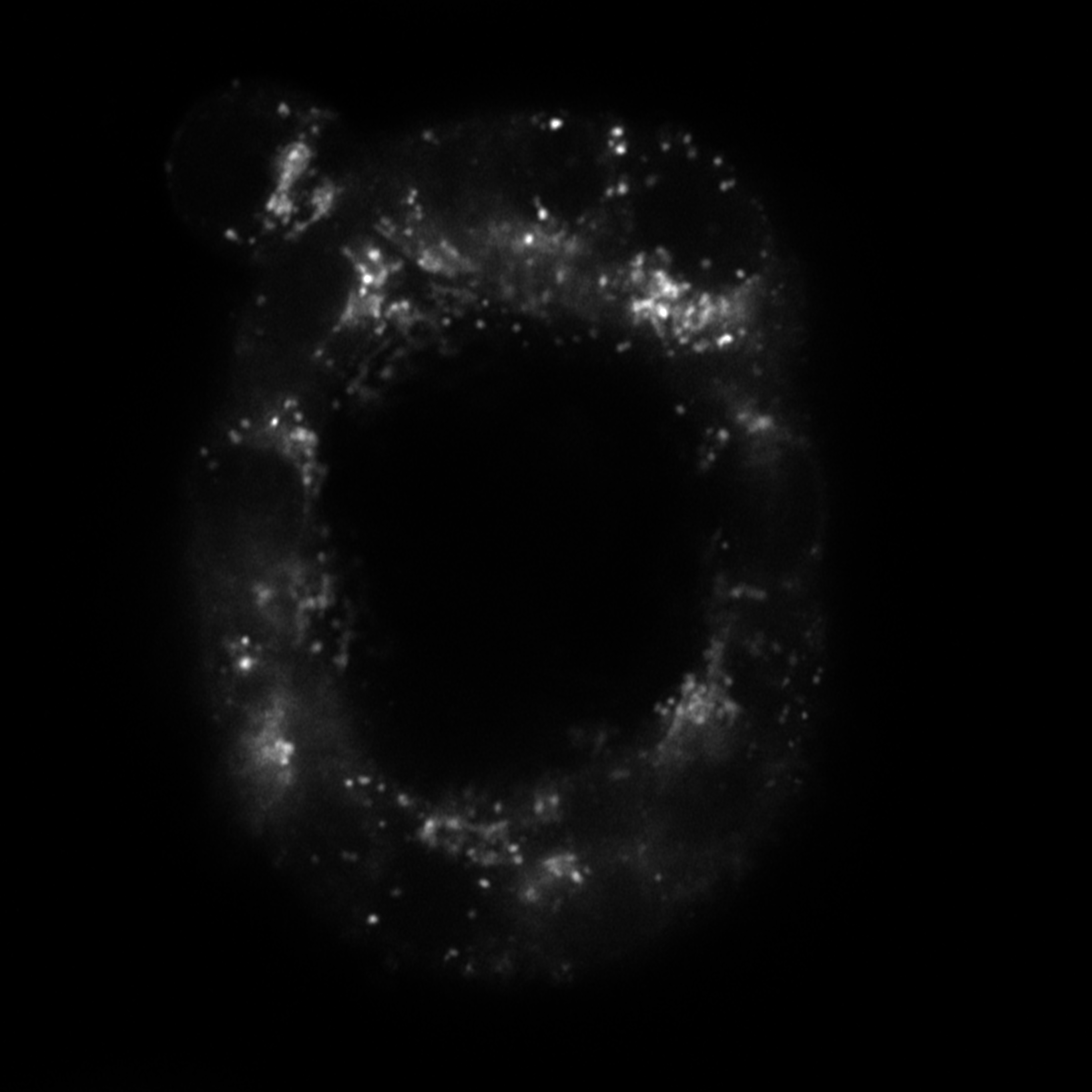

Supplement: Supplementary file 13 — Source data Fig. 3 [file 44318_2024_328_MOESM13_ESM.zip › Figure 3A_C1-WT DAY10 5_Airyscan Processing.tif]

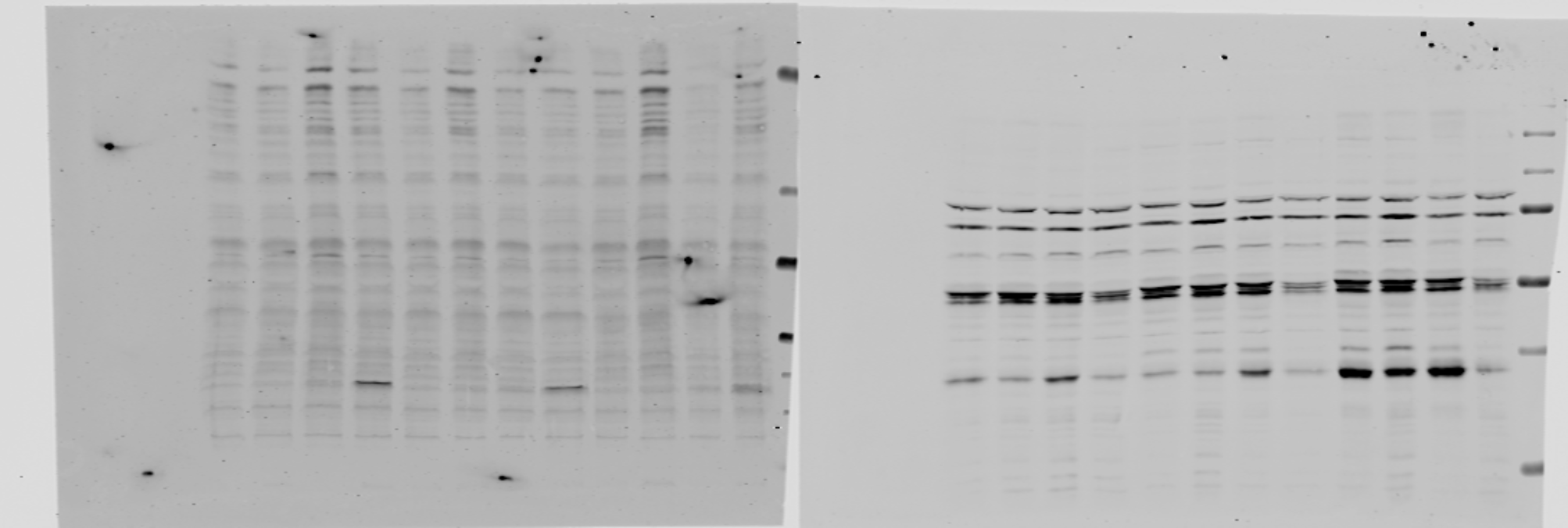

Supplement: Supplementary file 13 — Source data Fig. 3 [file 44318_2024_328_MOESM13_ESM.zip › Figure 3I_Image_0000136_01.tif]

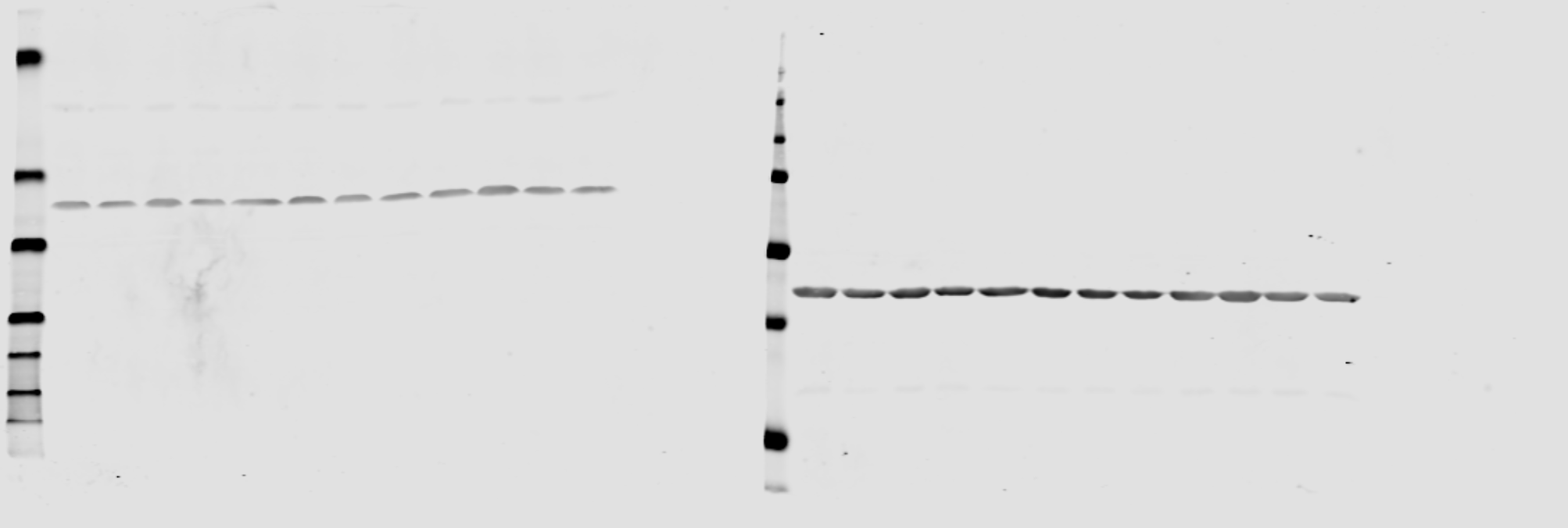

Supplement: Supplementary file 13 — Source data Fig. 3 [file 44318_2024_328_MOESM13_ESM.zip › Figure 3I_Image_Ima1geIDActin.tif]

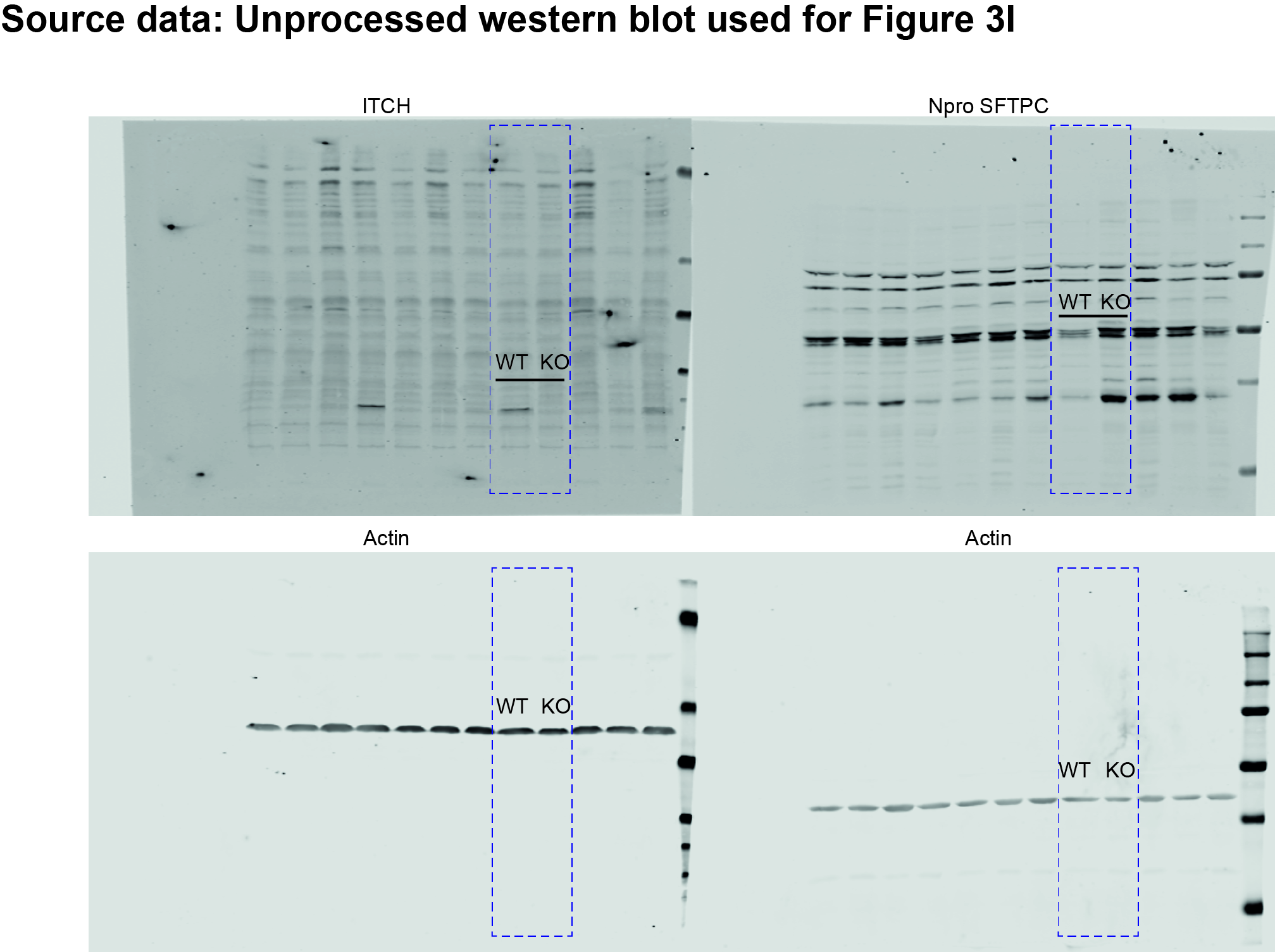

Supplement: Supplementary file 13 — Source data Fig. 3 [file 44318_2024_328_MOESM13_ESM.zip › Figure3I_WB images with relevant columns highlighted.tif]

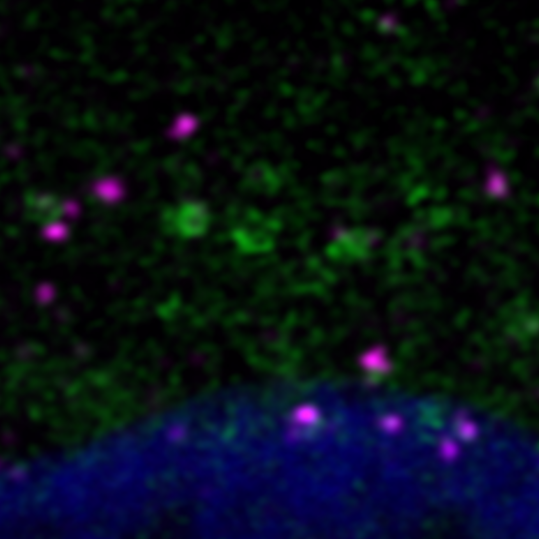

Supplement: Supplementary file 13 — Source data Fig. 3 [file 44318_2024_328_MOESM13_ESM.zip › Figure3J_Control proSPC EEA1 zoom.tif]

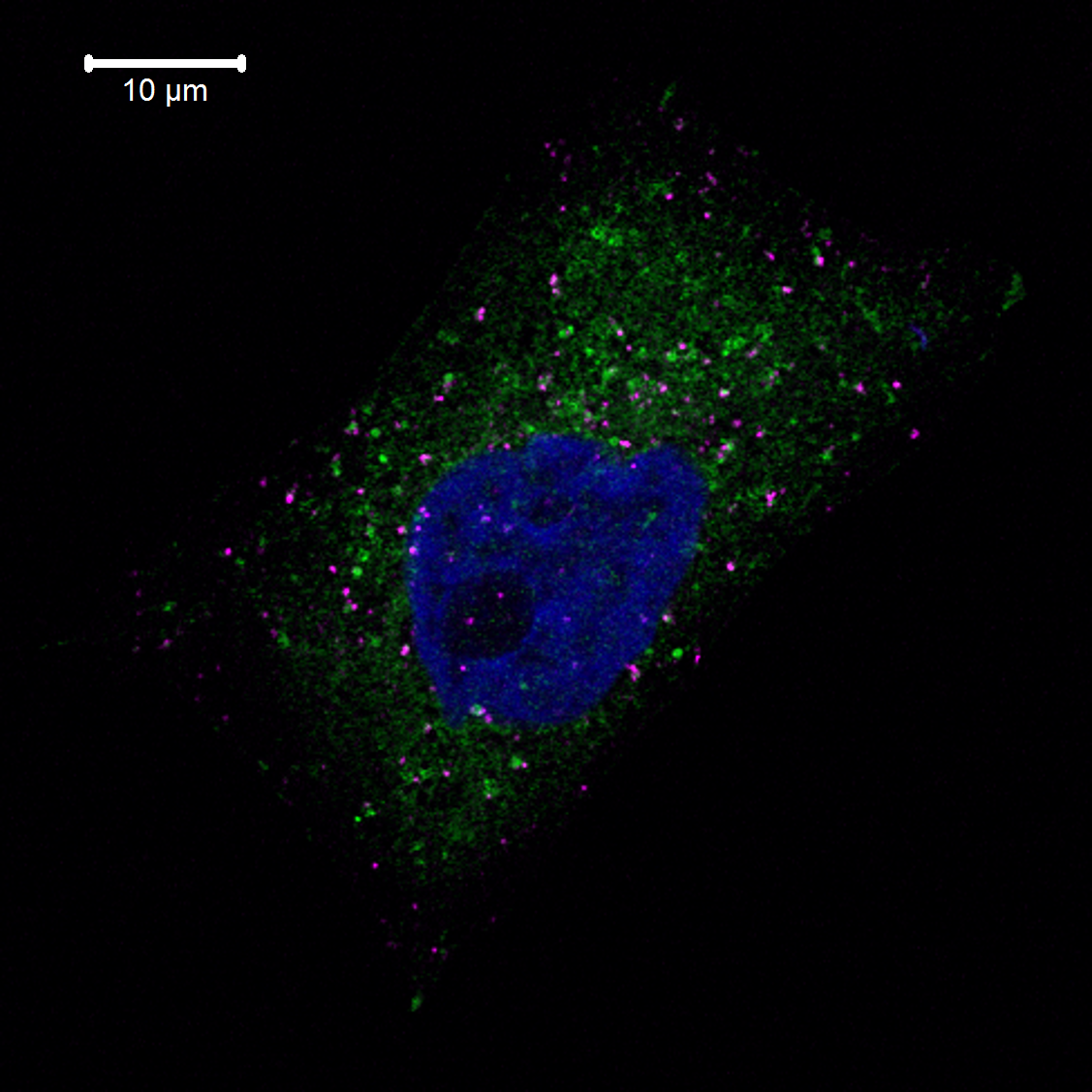

Supplement: Supplementary file 13 — Source data Fig. 3 [file 44318_2024_328_MOESM13_ESM.zip › Figure3J_Control proSPC EEA1.tif]

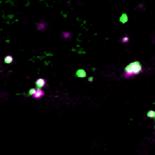

Supplement: Supplementary file 13 — Source data Fig. 3 [file 44318_2024_328_MOESM13_ESM.zip › Figure3J_Control proSPC LAMP3 zoom.tif]

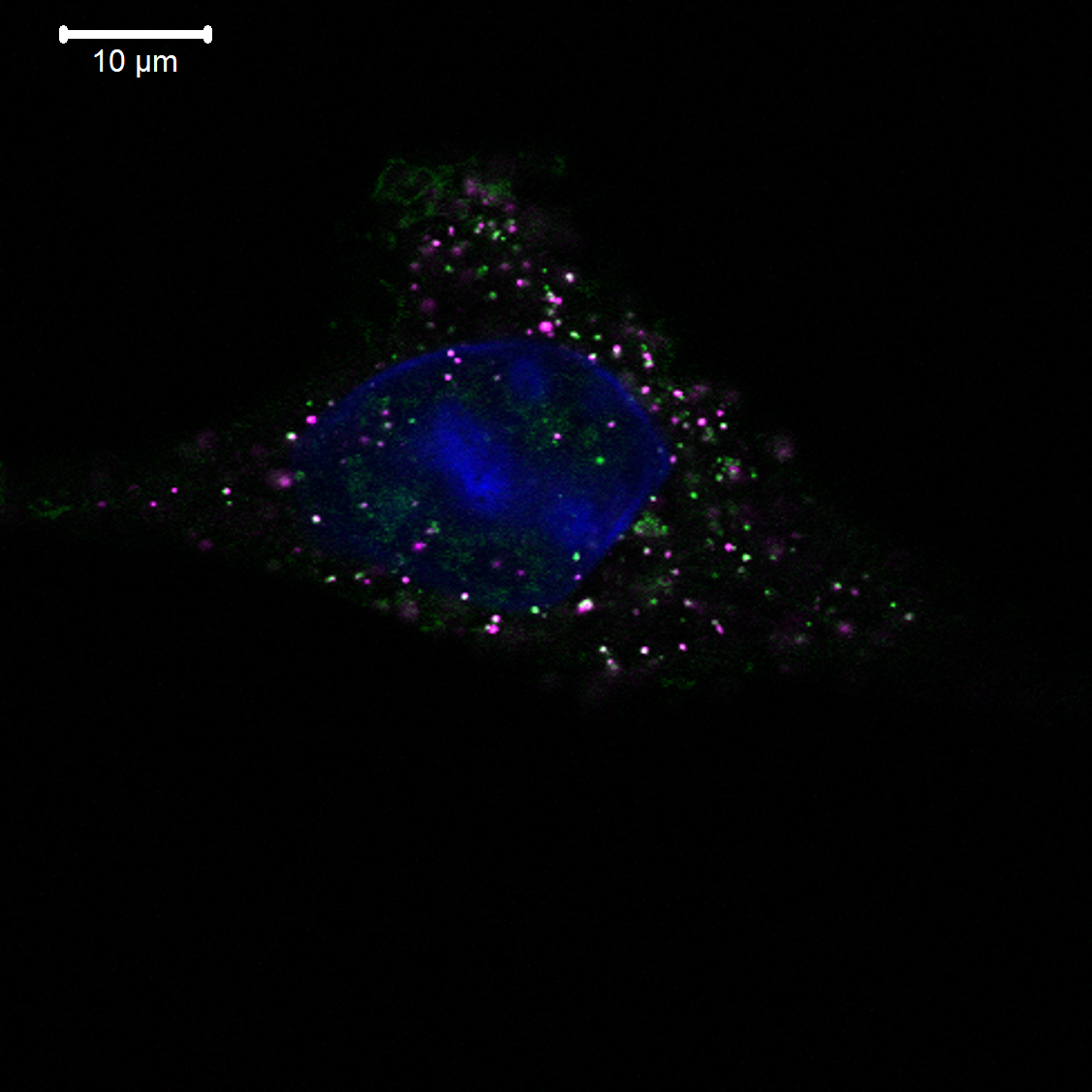

Supplement: Supplementary file 13 — Source data Fig. 3 [file 44318_2024_328_MOESM13_ESM.zip › Figure3J_Control proSPC LAMP3.tif]

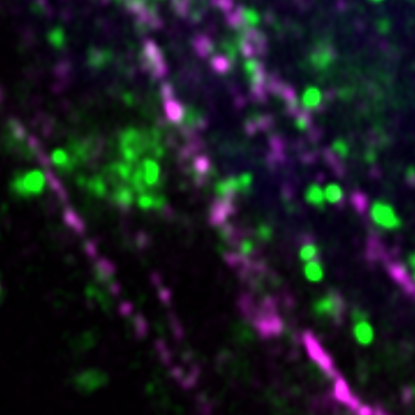

Supplement: Supplementary file 13 — Source data Fig. 3 [file 44318_2024_328_MOESM13_ESM.zip › Figure3J_Control proSPC MICALL1 zoom.tif]

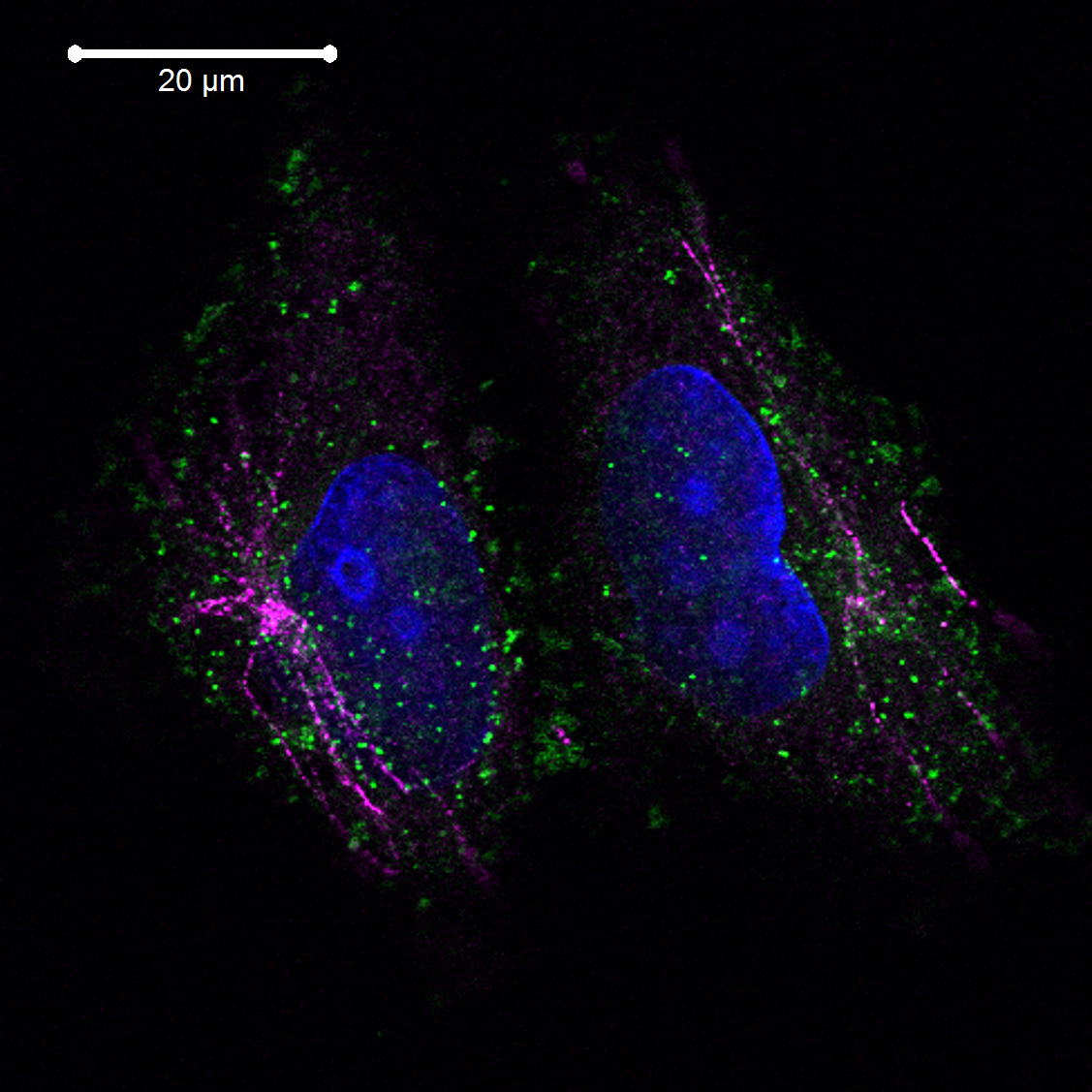

Supplement: Supplementary file 13 — Source data Fig. 3 [file 44318_2024_328_MOESM13_ESM.zip › Figure3J_Control proSPC MICALL1.tif]

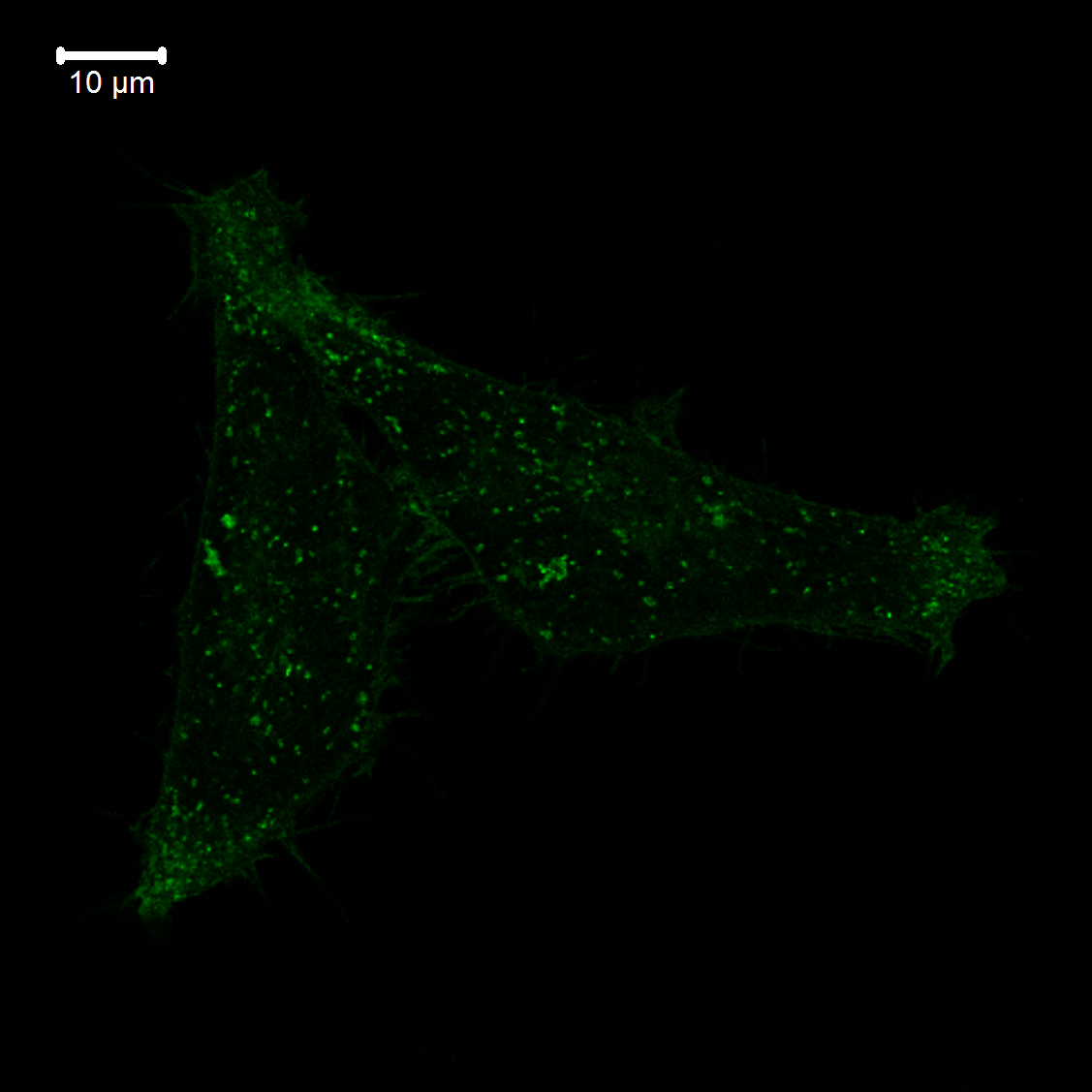

Supplement: Supplementary file 13 — Source data Fig. 3 [file 44318_2024_328_MOESM13_ESM.zip › Figure3J_Control proSPC.tif]

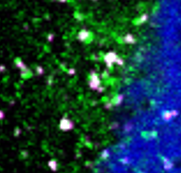

Supplement: Supplementary file 13 — Source data Fig. 3 [file 44318_2024_328_MOESM13_ESM.zip › Figure3J_ITCH KO proSPC EEA1 zoom.tif]

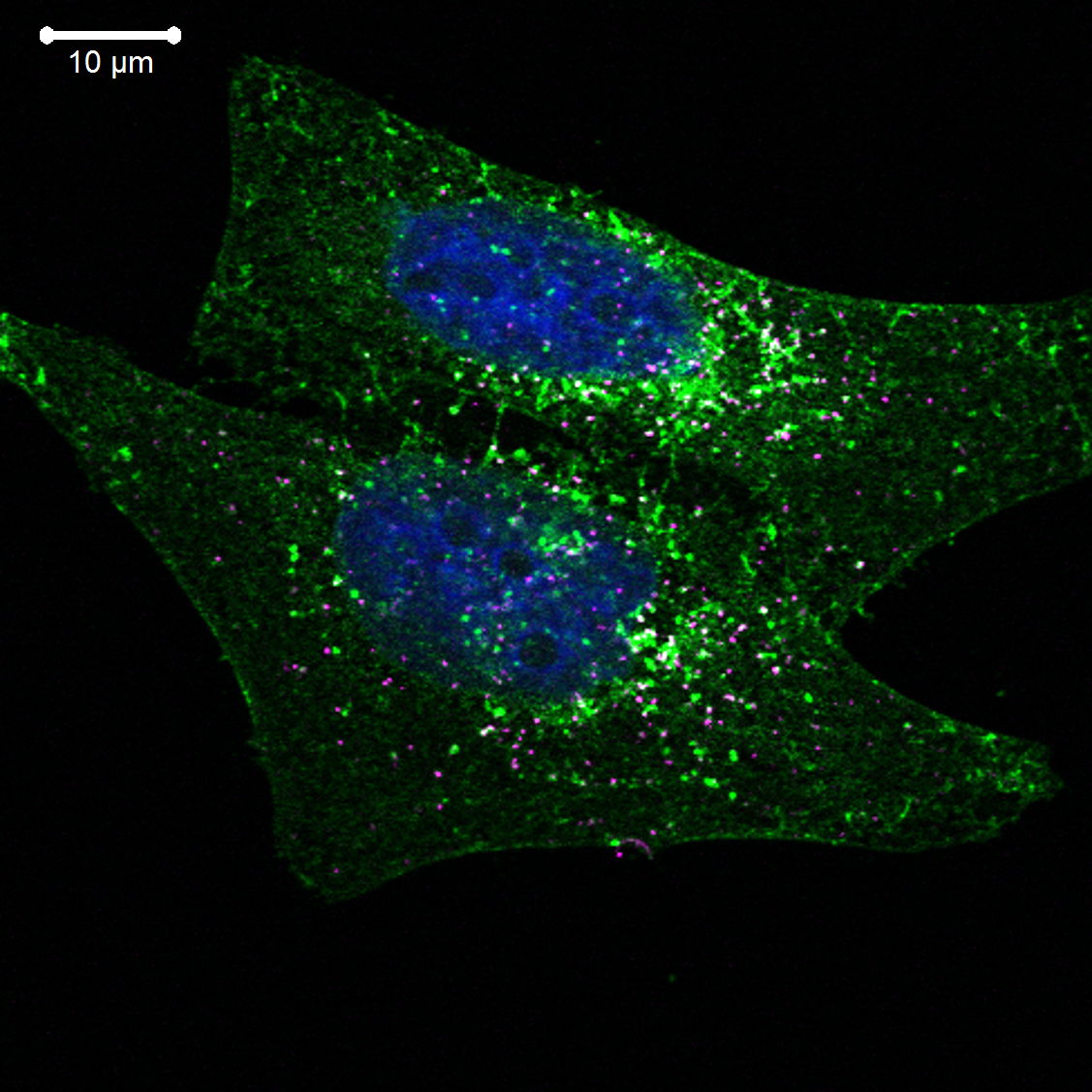

Supplement: Supplementary file 13 — Source data Fig. 3 [file 44318_2024_328_MOESM13_ESM.zip › Figure3J_ITCH KO proSPC EEA1.tif]

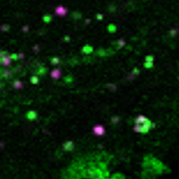

Supplement: Supplementary file 13 — Source data Fig. 3 [file 44318_2024_328_MOESM13_ESM.zip › Figure3J_ITCH KO proSPC LAMP3 zoom.tif]

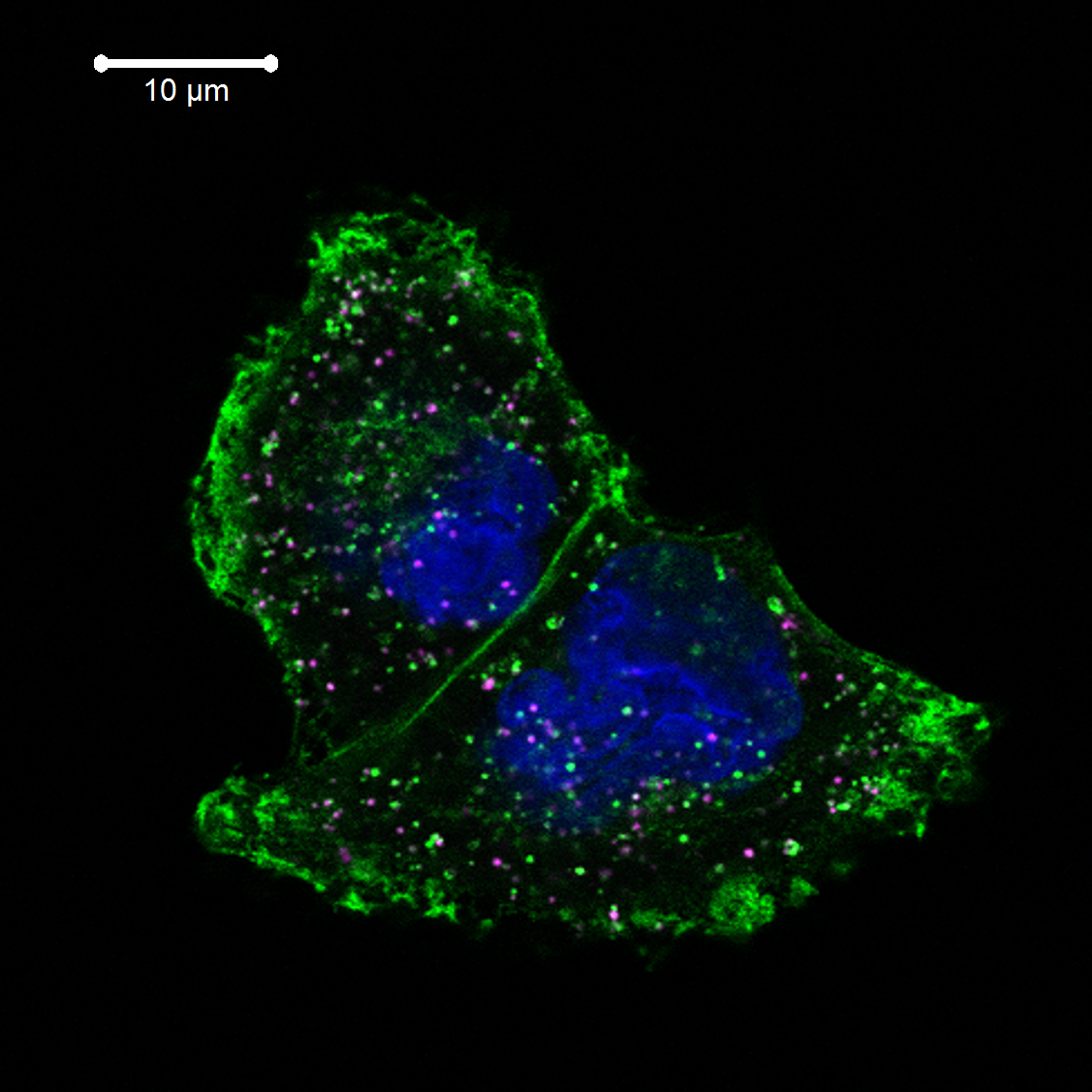

Supplement: Supplementary file 13 — Source data Fig. 3 [file 44318_2024_328_MOESM13_ESM.zip › Figure3J_ITCH KO proSPC LAMP3.tif]

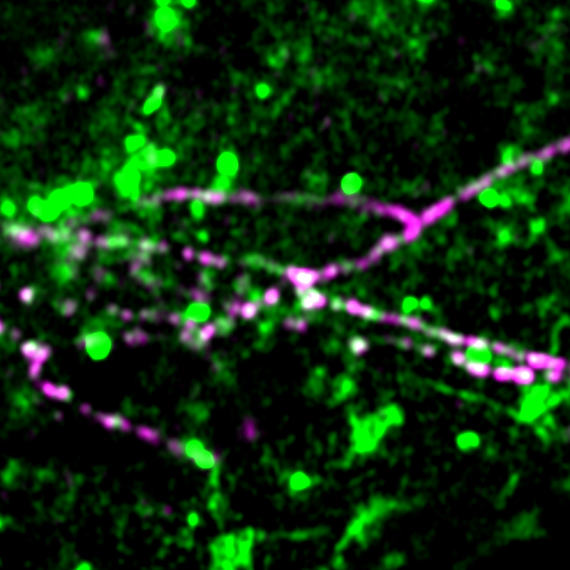

Supplement: Supplementary file 13 — Source data Fig. 3 [file 44318_2024_328_MOESM13_ESM.zip › Figure3J_ITCH KO proSPC MICALL1 zoom.tif]

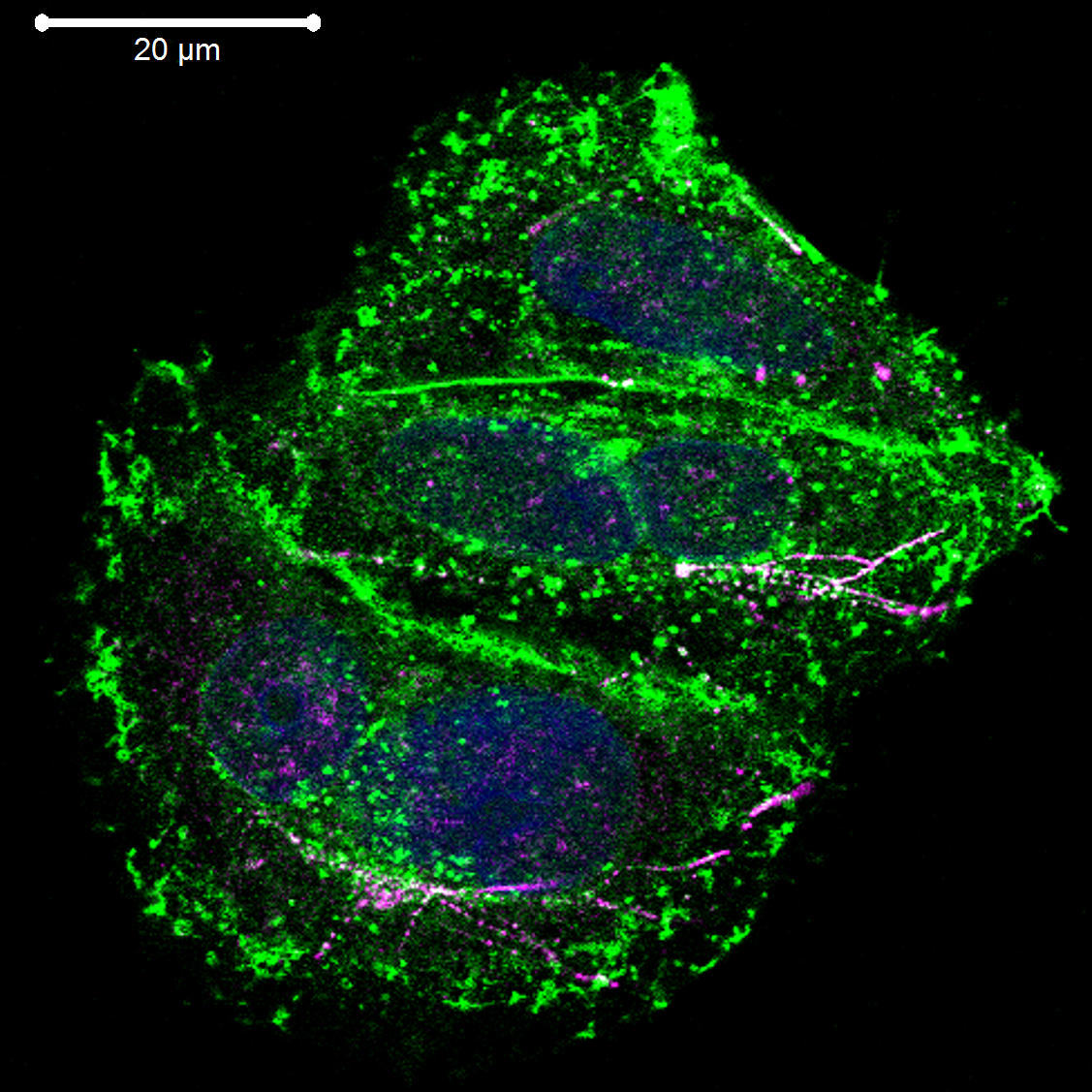

Supplement: Supplementary file 13 — Source data Fig. 3 [file 44318_2024_328_MOESM13_ESM.zip › Figure3J_ITCH KO proSPC MICALL1.tif]

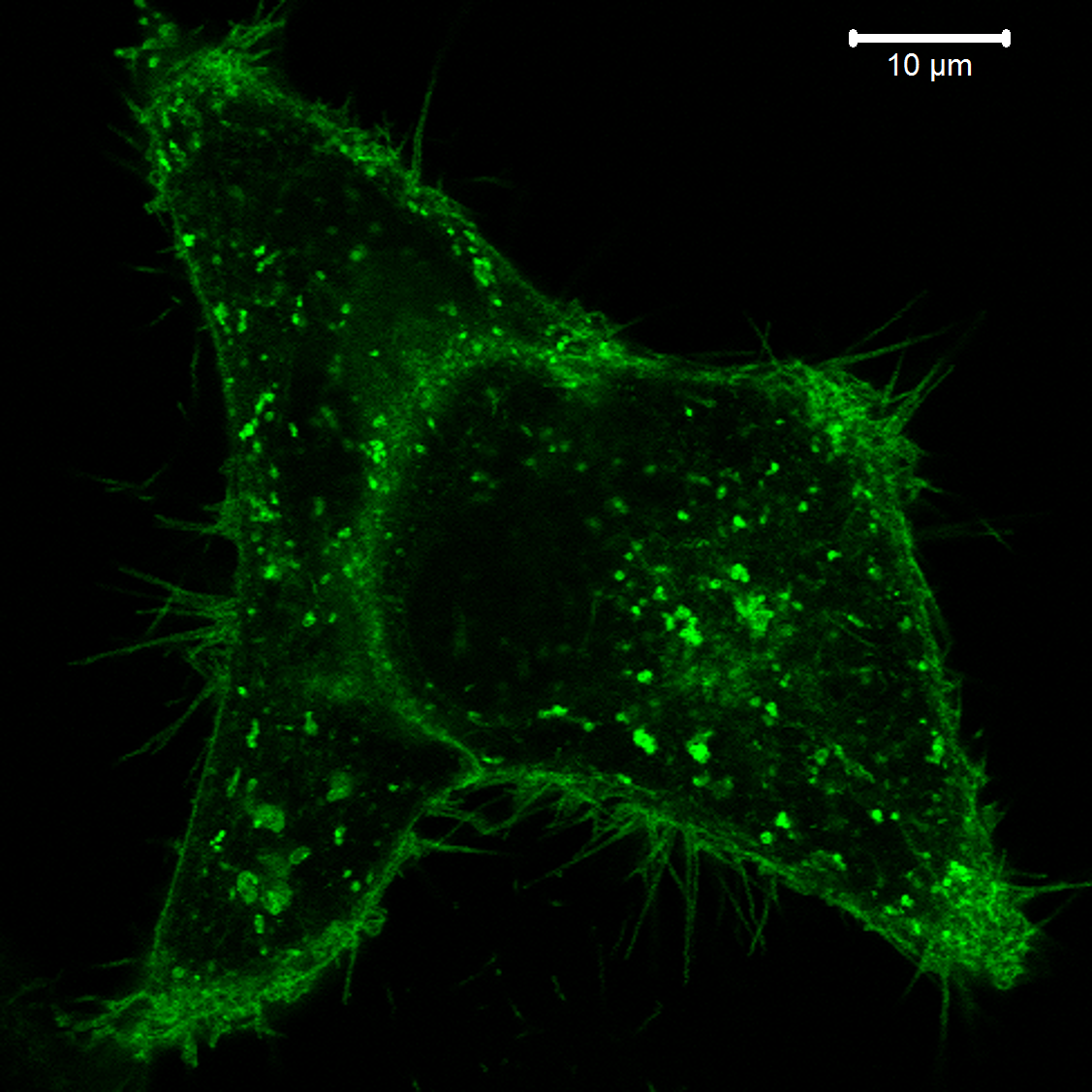

Supplement: Supplementary file 13 — Source data Fig. 3 [file 44318_2024_328_MOESM13_ESM.zip › Figure3J_ITCH KO proSPC.tif]

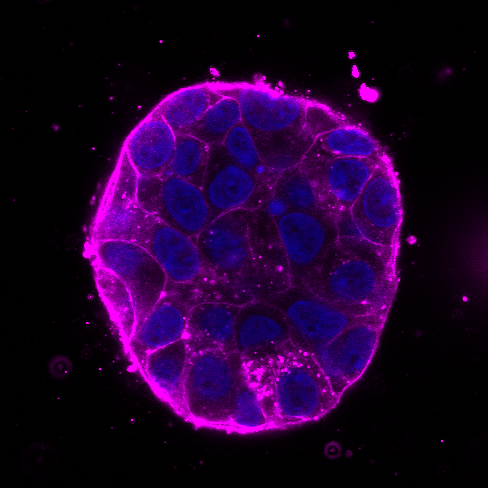

Supplement: Supplementary file 14 — Source data Fig. 4 [file 44318_2024_328_MOESM14_ESM.zip › Figure 4C_sgITCH proSFTPC.tif]

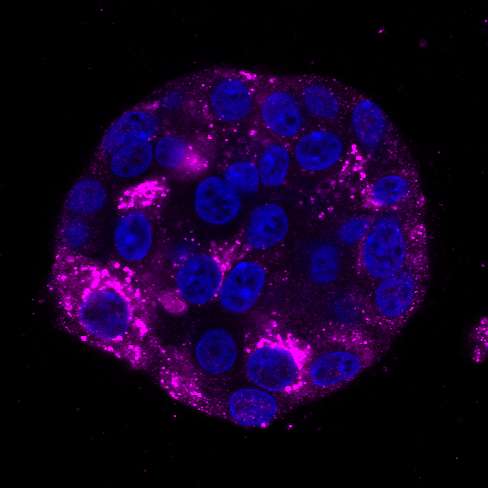

Supplement: Supplementary file 14 — Source data Fig. 4 [file 44318_2024_328_MOESM14_ESM.zip › Figure 4C_sgNT proSFTPC.tif]

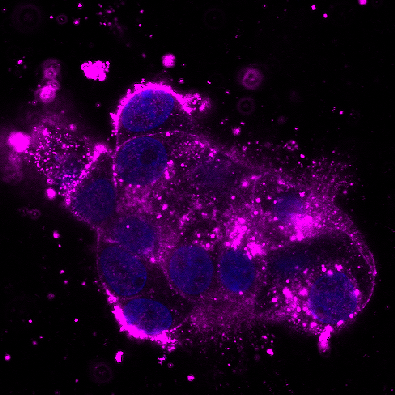

Supplement: Supplementary file 14 — Source data Fig. 4 [file 44318_2024_328_MOESM14_ESM.zip › Figure 4C_sgUBE2N proSFTPC.tif]

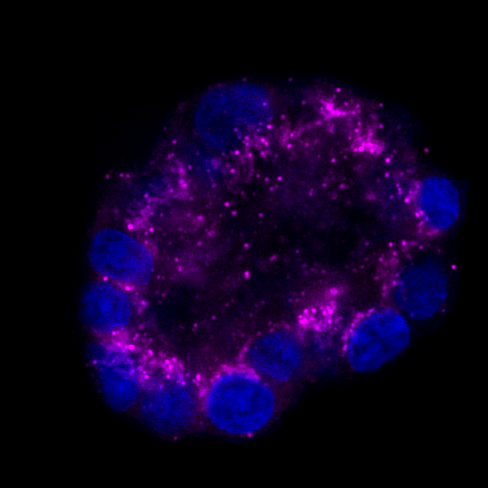

Supplement: Supplementary file 14 — Source data Fig. 4 [file 44318_2024_328_MOESM14_ESM.zip › Figure 4H_sgITCH recovery.tif]

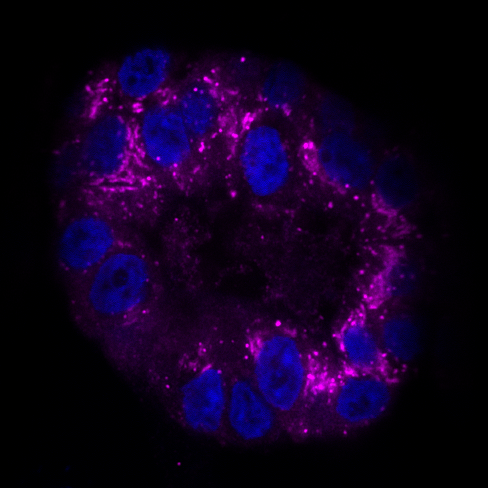

Supplement: Supplementary file 14 — Source data Fig. 4 [file 44318_2024_328_MOESM14_ESM.zip › Figure 4H_sgNT recovery.tif]

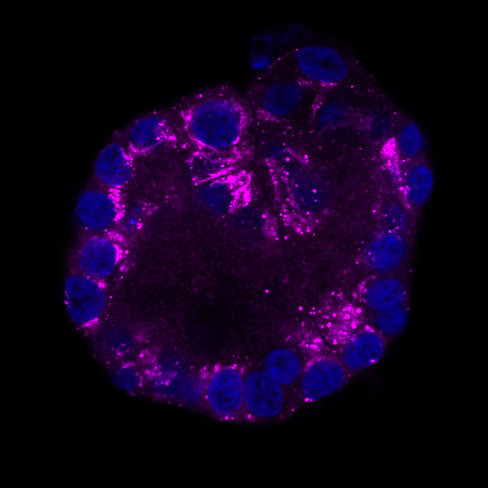

Supplement: Supplementary file 14 — Source data Fig. 4 [file 44318_2024_328_MOESM14_ESM.zip › Figure 4H_sgUBE2N recovery.tif]

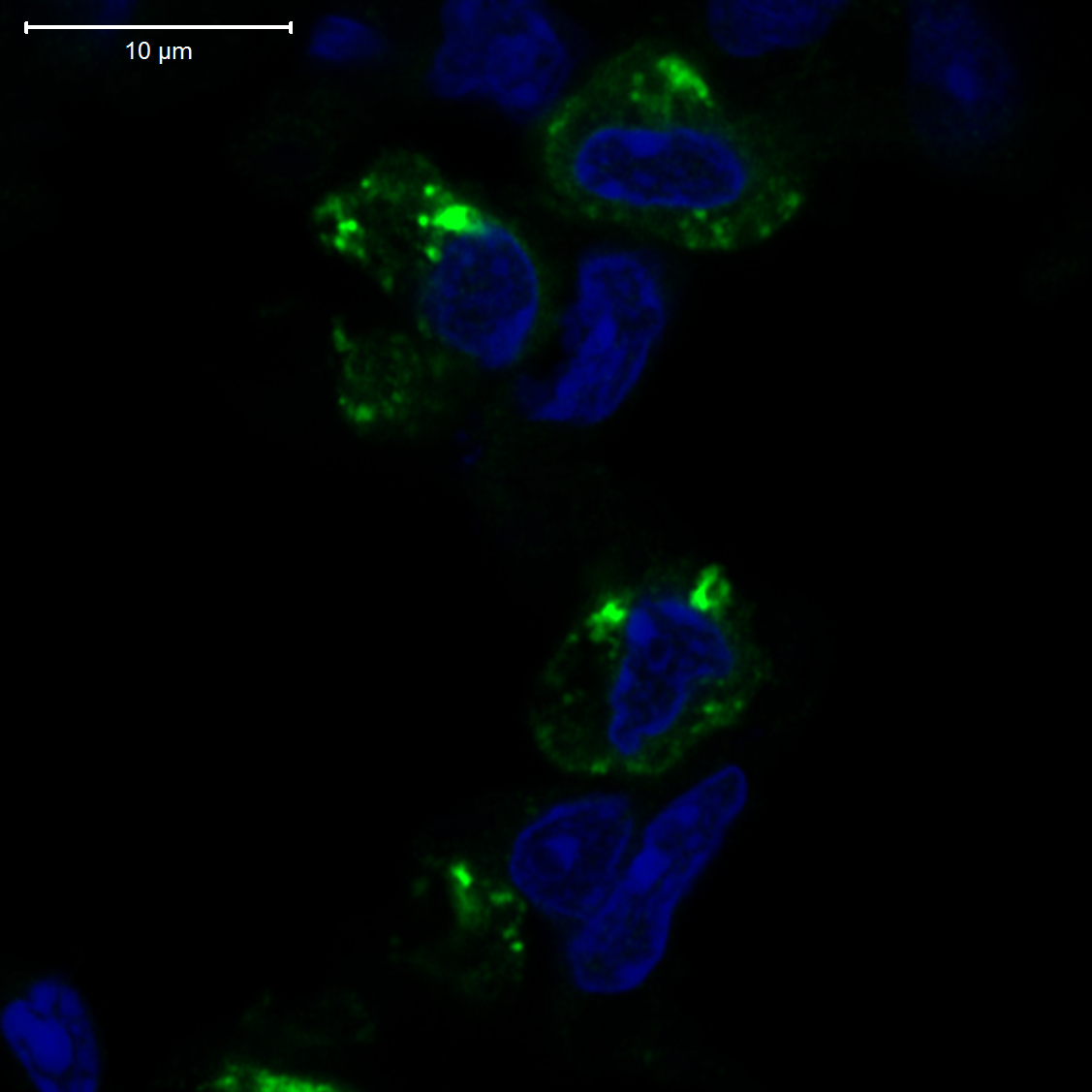

Supplement: Supplementary file 14 — Source data Fig. 4 [file 44318_2024_328_MOESM14_ESM.zip › Figure 4K_control 6 zoom_Airyscan Processing.tif]

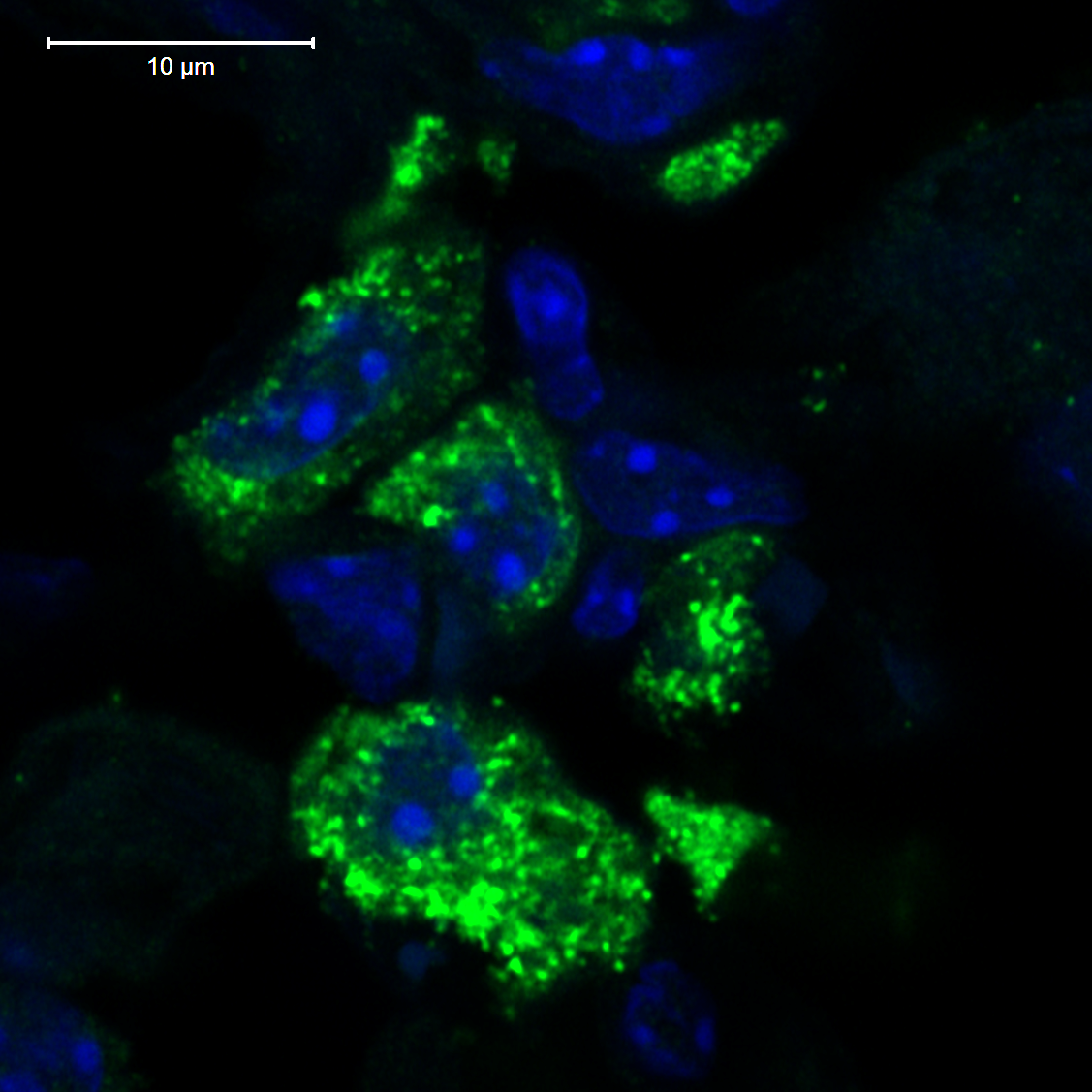

Supplement: Supplementary file 14 — Source data Fig. 4 [file 44318_2024_328_MOESM14_ESM.zip › Figure 4K_ko 1992 2 zoom_Airyscan Processing.tif]

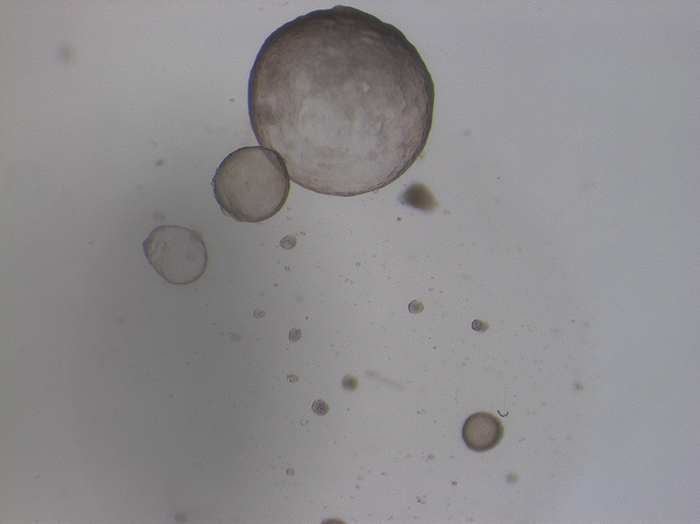

Supplement: Supplementary file 15 — Source data Fig. EV1 [file 44318_2024_328_MOESM15_ESM.zip › Extended Data Figure 1A. Airway_3A2-GFP_Brightfield_airway medium.jpg]

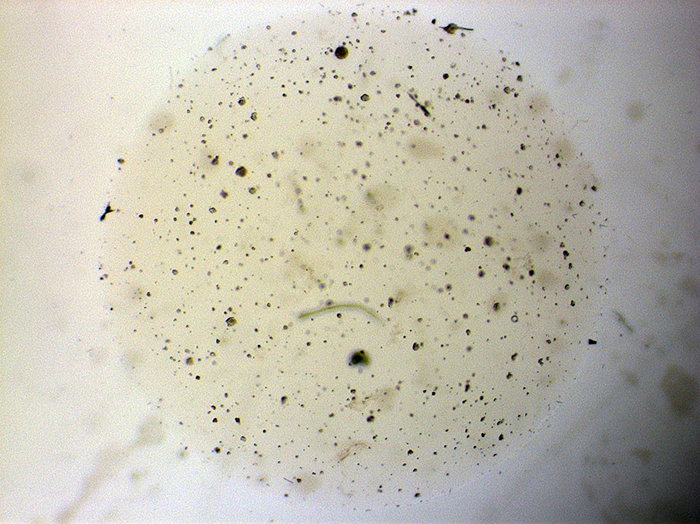

Supplement: Supplementary file 15 — Source data Fig. EV1 [file 44318_2024_328_MOESM15_ESM.zip › Extended Data Figure 1A. Airway_3A2-GFP_Brightfield_AT2 medium.jpg]

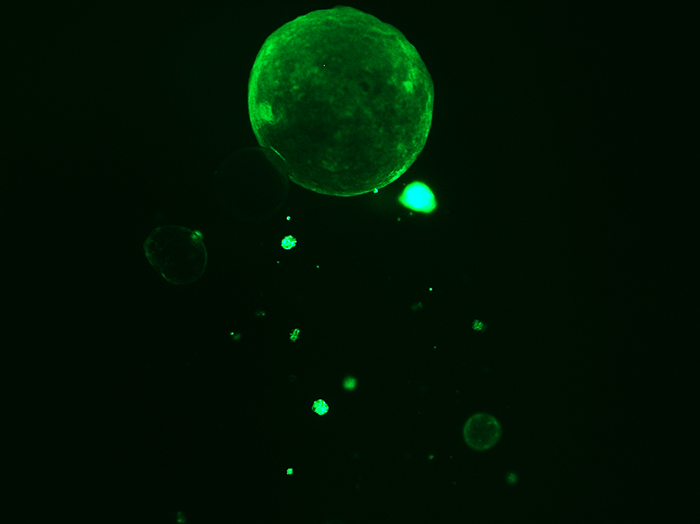

Supplement: Supplementary file 15 — Source data Fig. EV1 [file 44318_2024_328_MOESM15_ESM.zip › Extended Data Figure 1A. Airway_3A2-GFP_GFP_airway medium.jpg]

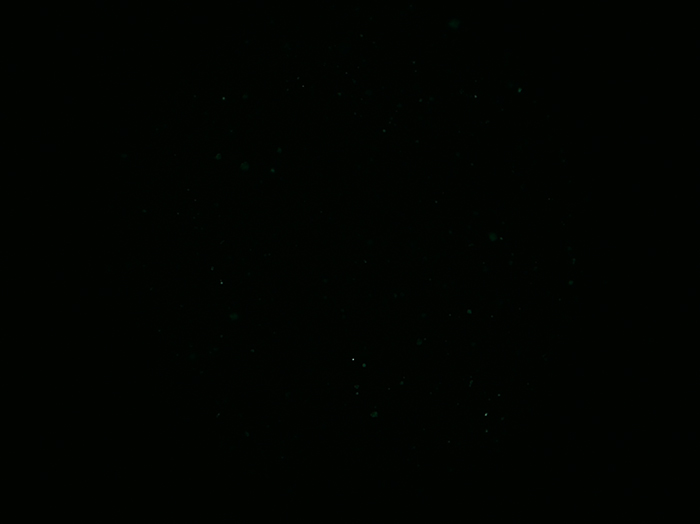

Supplement: Supplementary file 15 — Source data Fig. EV1 [file 44318_2024_328_MOESM15_ESM.zip › Extended Data Figure 1A. Airway_3A2-GFP_GFP_AT2 medium.jpg]

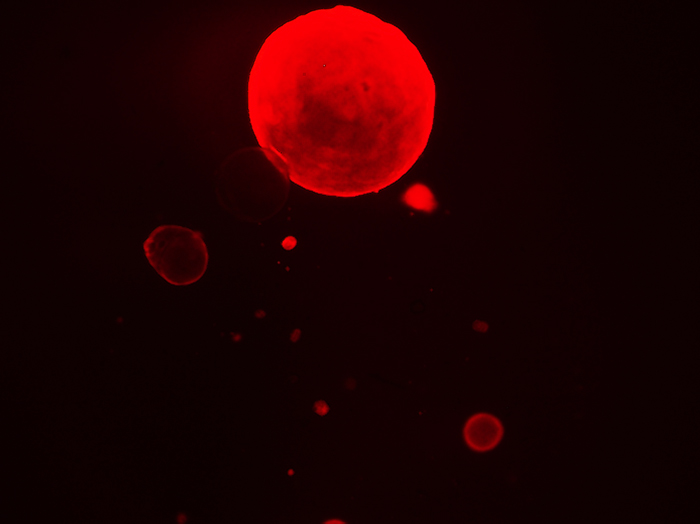

Supplement: Supplementary file 15 — Source data Fig. EV1 [file 44318_2024_328_MOESM15_ESM.zip › Extended Data Figure 1A. Airway_3A2-GFP_RFP_airway medium.jpg]

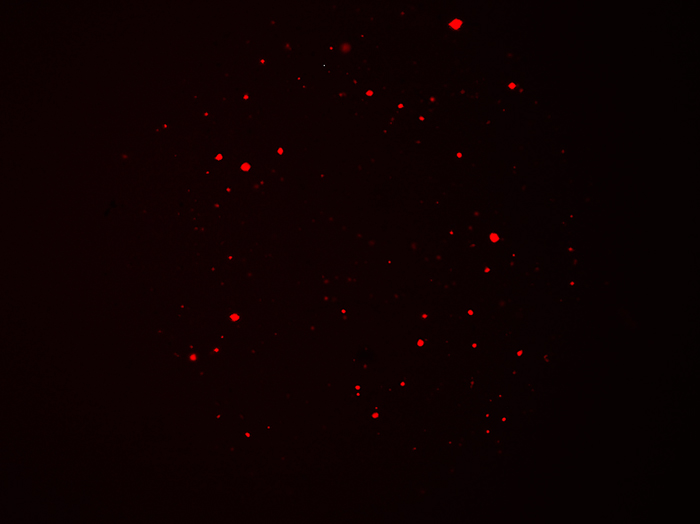

Supplement: Supplementary file 15 — Source data Fig. EV1 [file 44318_2024_328_MOESM15_ESM.zip › Extended Data Figure 1A. Airway_3A2-GFP_RFP_AT2 medium.jpg]

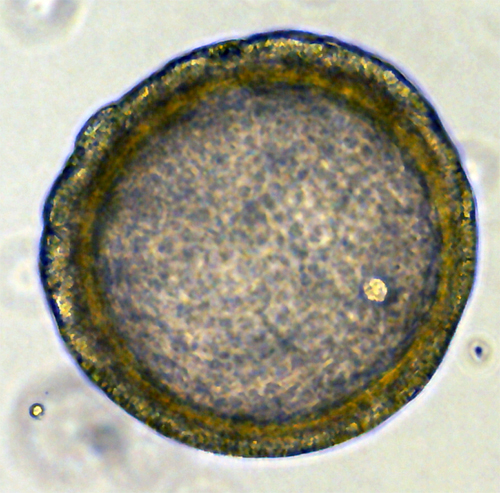

Supplement: Supplementary file 15 — Source data Fig. EV1 [file 44318_2024_328_MOESM15_ESM.zip › Extended Data Figure 1A. Airway_3week_Airway medium.jpg]

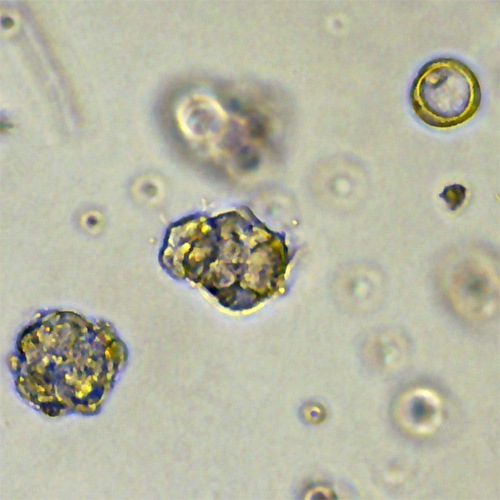

Supplement: Supplementary file 15 — Source data Fig. EV1 [file 44318_2024_328_MOESM15_ESM.zip › Extended Data Figure 1A. Airway_3week_AT2 medium.jpg]

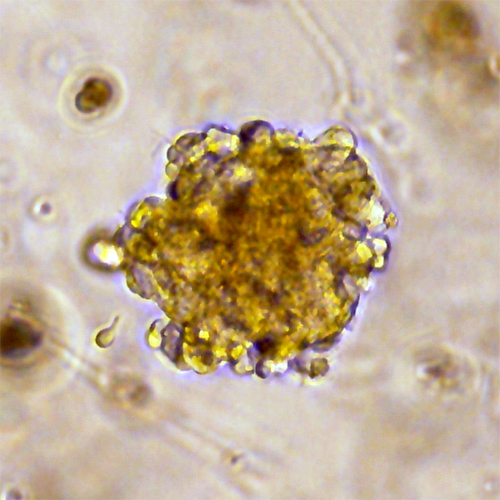

Supplement: Supplementary file 15 — Source data Fig. EV1 [file 44318_2024_328_MOESM15_ESM.zip › Extended Data Figure 1A. Tip_3week_AT2 medium.jpg]

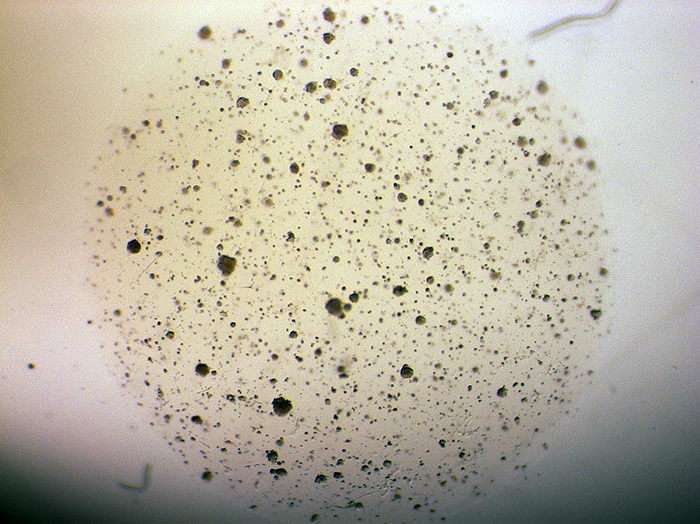

Supplement: Supplementary file 15 — Source data Fig. EV1 [file 44318_2024_328_MOESM15_ESM.zip › Extended Data Figure 1A. Tip_SPC-GFP_Brightfield.jpg]

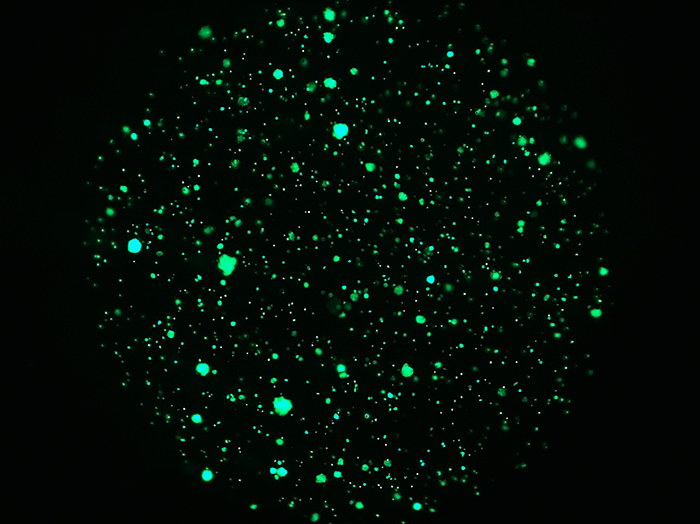

Supplement: Supplementary file 15 — Source data Fig. EV1 [file 44318_2024_328_MOESM15_ESM.zip › Extended Data Figure 1A. Tip_SPC-GFP_GFP.jpg]

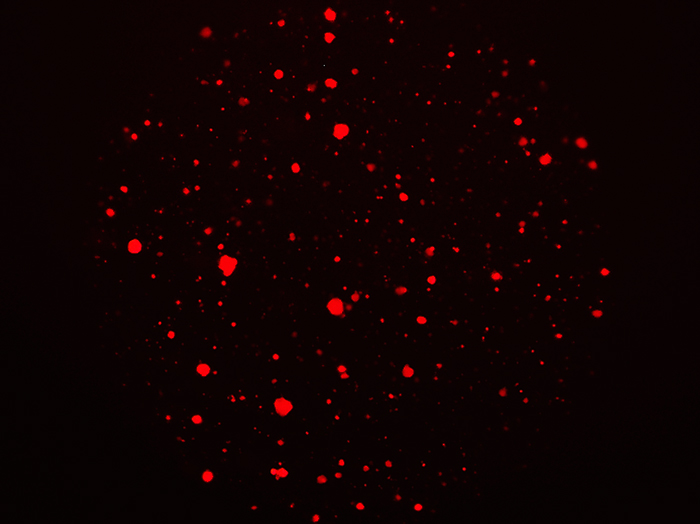

Supplement: Supplementary file 15 — Source data Fig. EV1 [file 44318_2024_328_MOESM15_ESM.zip › Extended Data Figure 1A. Tip_SPC-GFP_RFP.jpg]

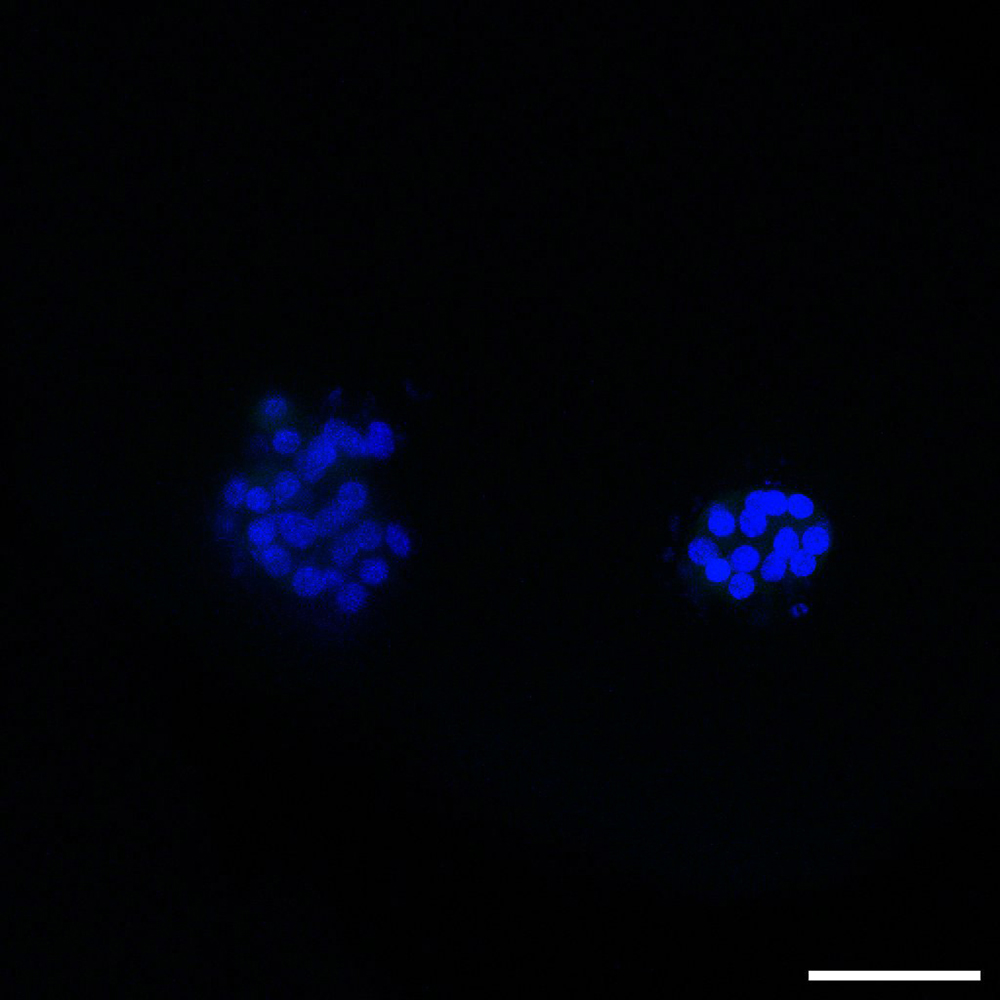

Supplement: Supplementary file 15 — Source data Fig. EV1 [file 44318_2024_328_MOESM15_ESM.zip › Extended Data Figure 1C. SCGB3A2-GFP_AT2 medium-1.jpg]

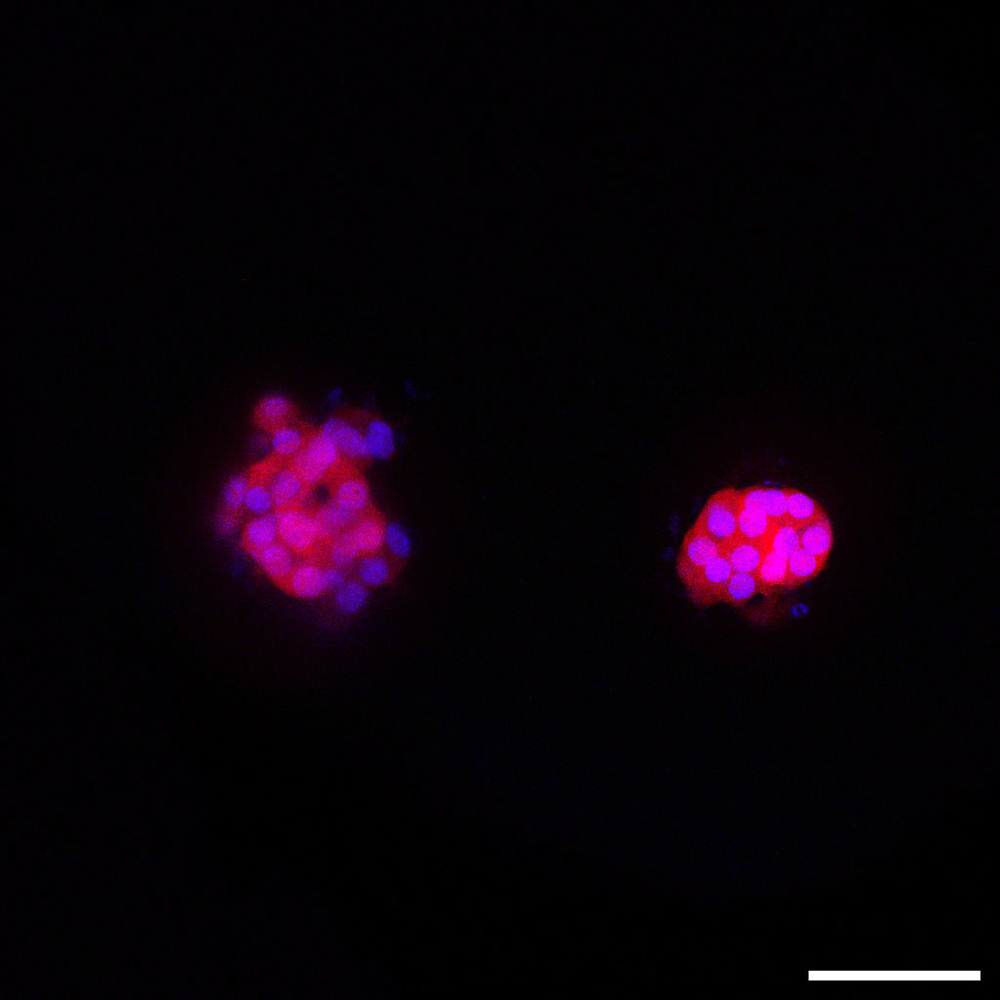

Supplement: Supplementary file 15 — Source data Fig. EV1 [file 44318_2024_328_MOESM15_ESM.zip › Extended Data Figure 1C. SCGB3A2-GFP_AT2 medium-2.jpg]

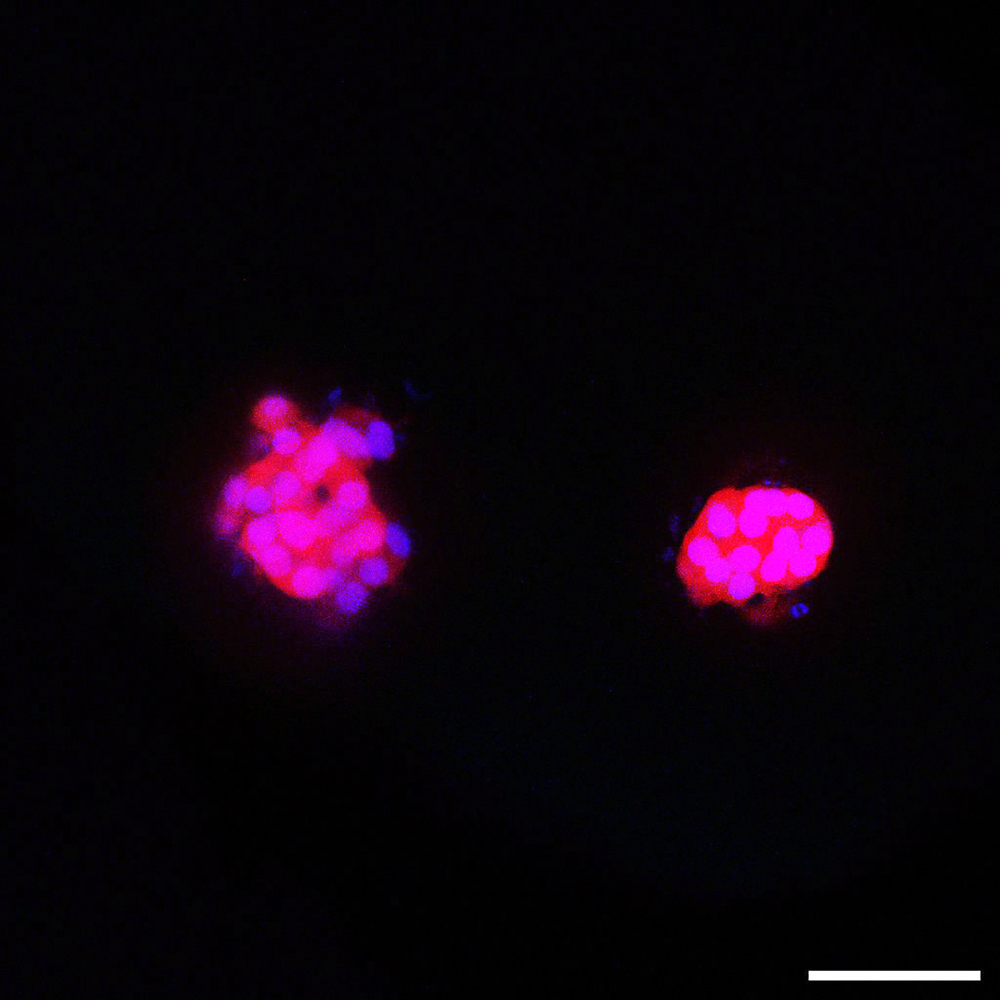

Supplement: Supplementary file 15 — Source data Fig. EV1 [file 44318_2024_328_MOESM15_ESM.zip › Extended Data Figure 1C. SCGB3A2-GFP_AT2 medium-3.jpg]

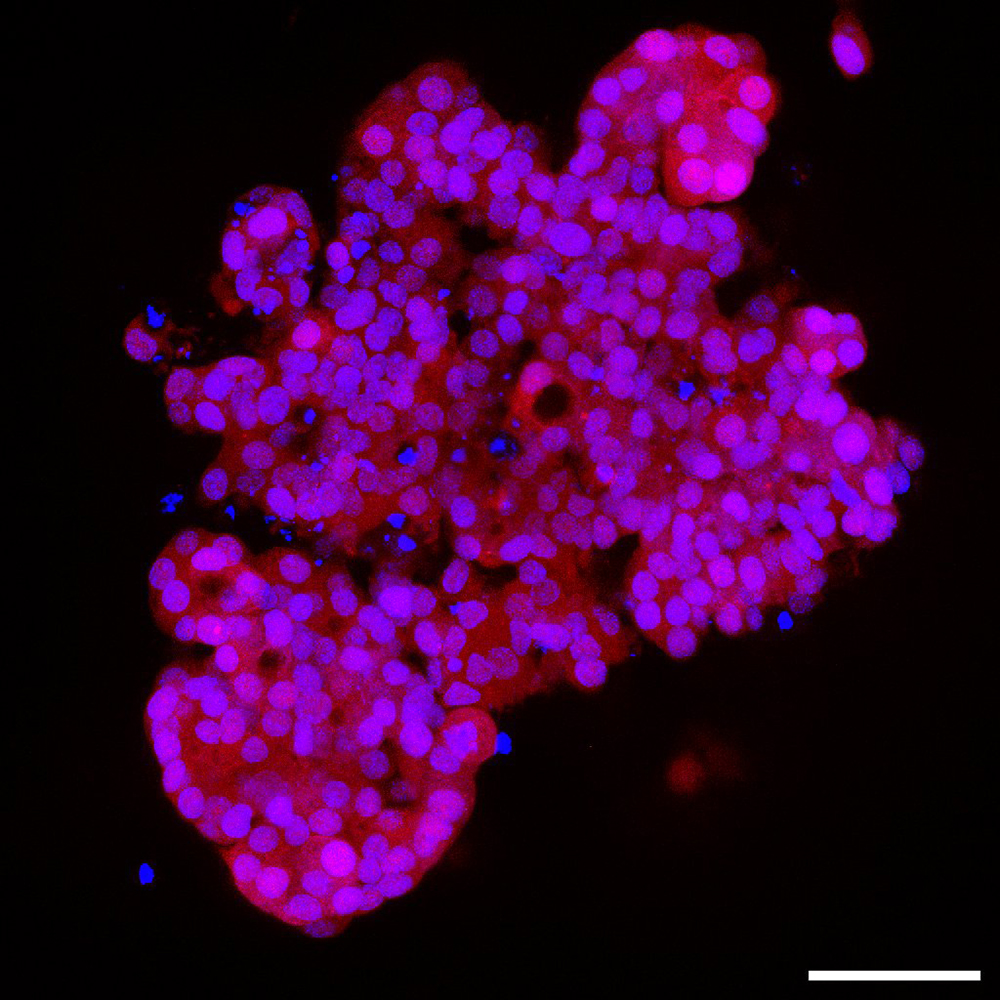

Supplement: Supplementary file 15 — Source data Fig. EV1 [file 44318_2024_328_MOESM15_ESM.zip › Extended Data Figure 1C. SPC-GFP_AT2 medium-1.jpg]

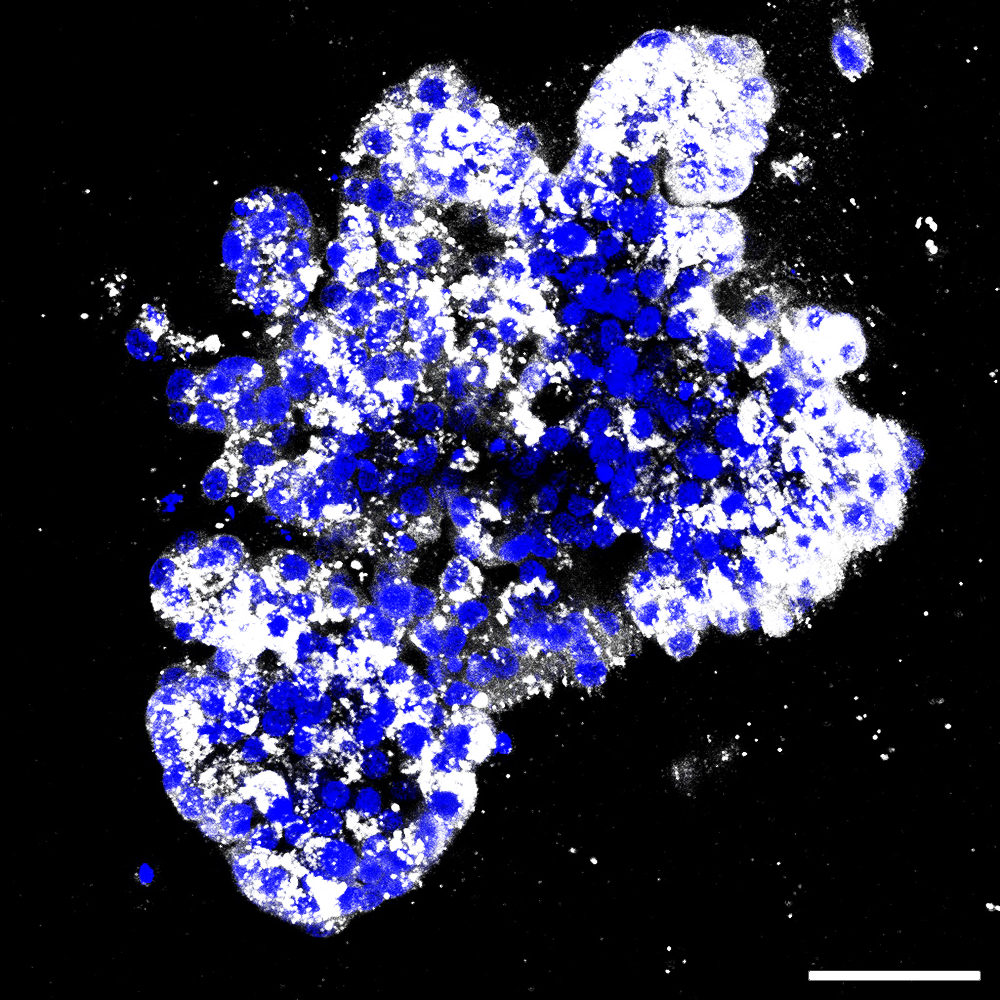

Supplement: Supplementary file 15 — Source data Fig. EV1 [file 44318_2024_328_MOESM15_ESM.zip › Extended Data Figure 1C. SPC-GFP_AT2 medium-2.jpg]

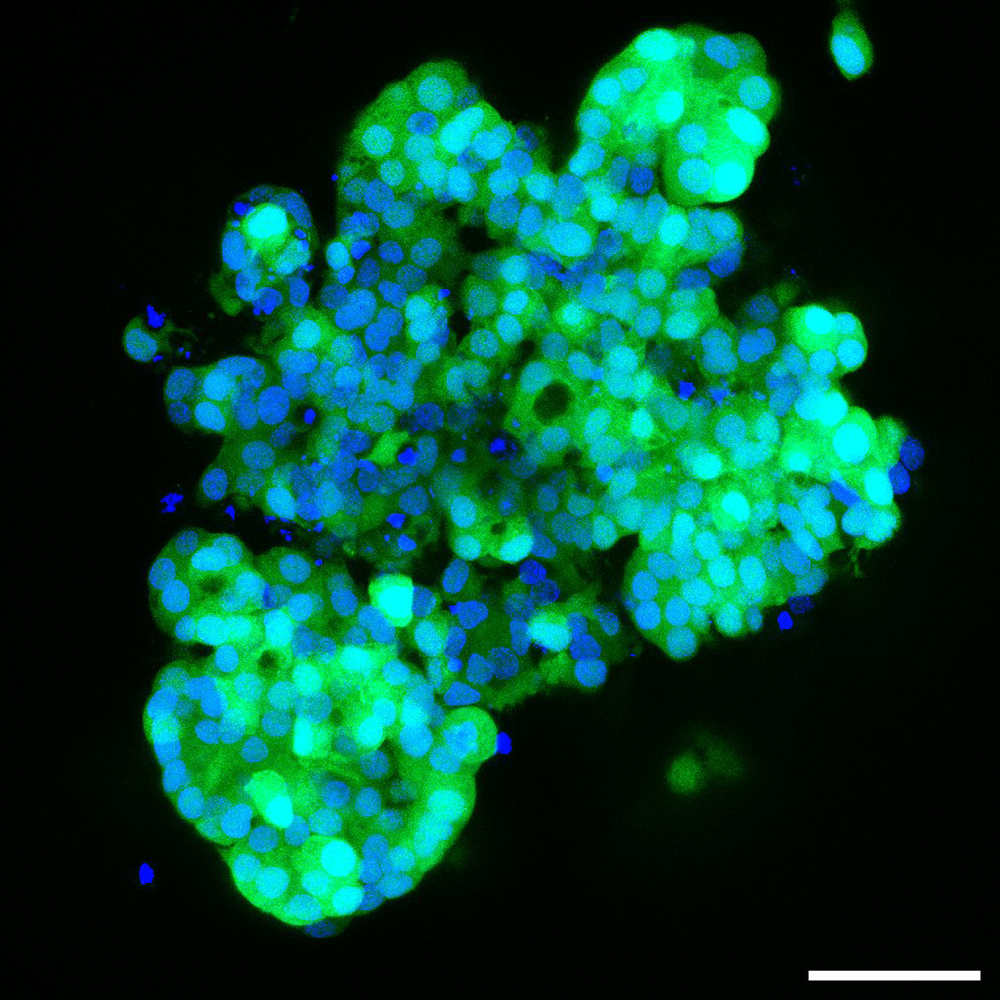

Supplement: Supplementary file 15 — Source data Fig. EV1 [file 44318_2024_328_MOESM15_ESM.zip › Extended Data Figure 1C. SPC-GFP_AT2 medium-3.jpg]

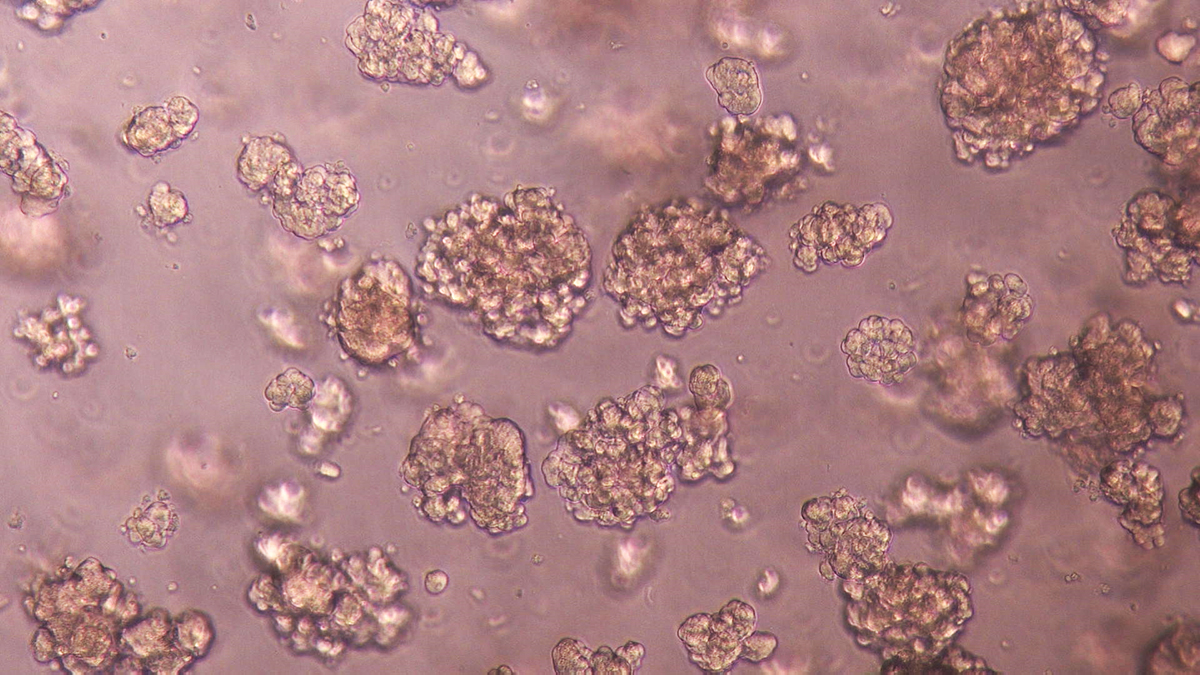

Supplement: Supplementary file 15 — Source data Fig. EV1 [file 44318_2024_328_MOESM15_ESM.zip › Extended Data Figure 1D. AT2_early_passages-1_Brightfield.jpg]

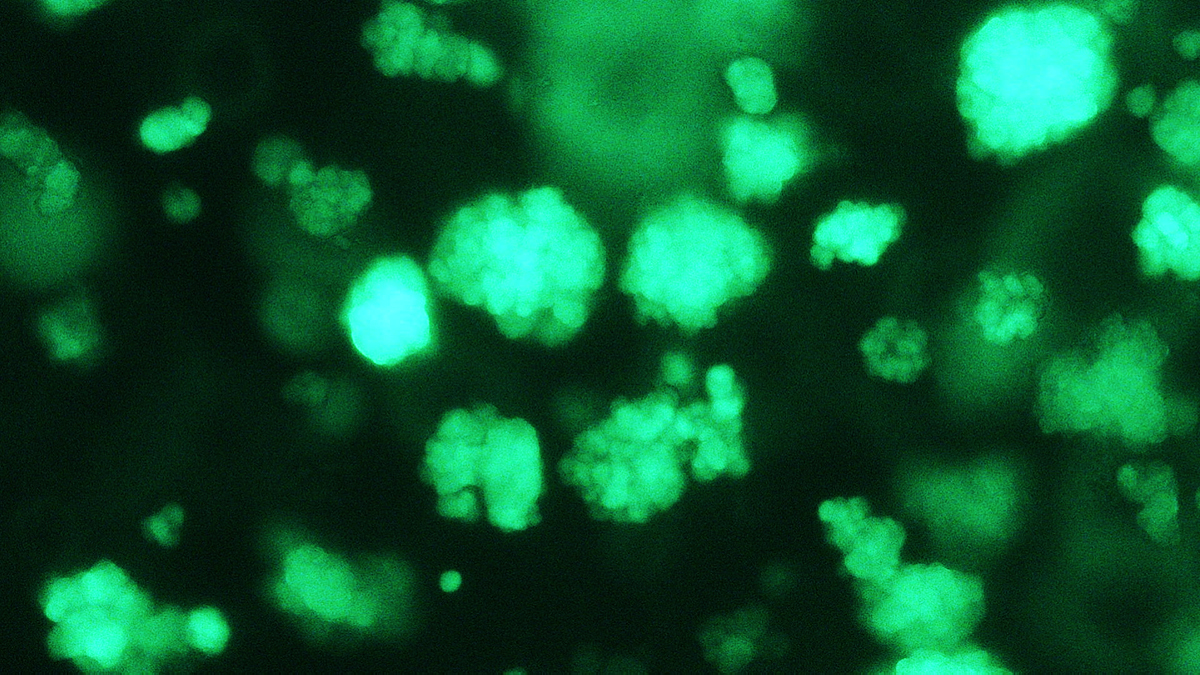

Supplement: Supplementary file 15 — Source data Fig. EV1 [file 44318_2024_328_MOESM15_ESM.zip › Extended Data Figure 1D. AT2_early_passages-1_SPC-GFP.jpg]

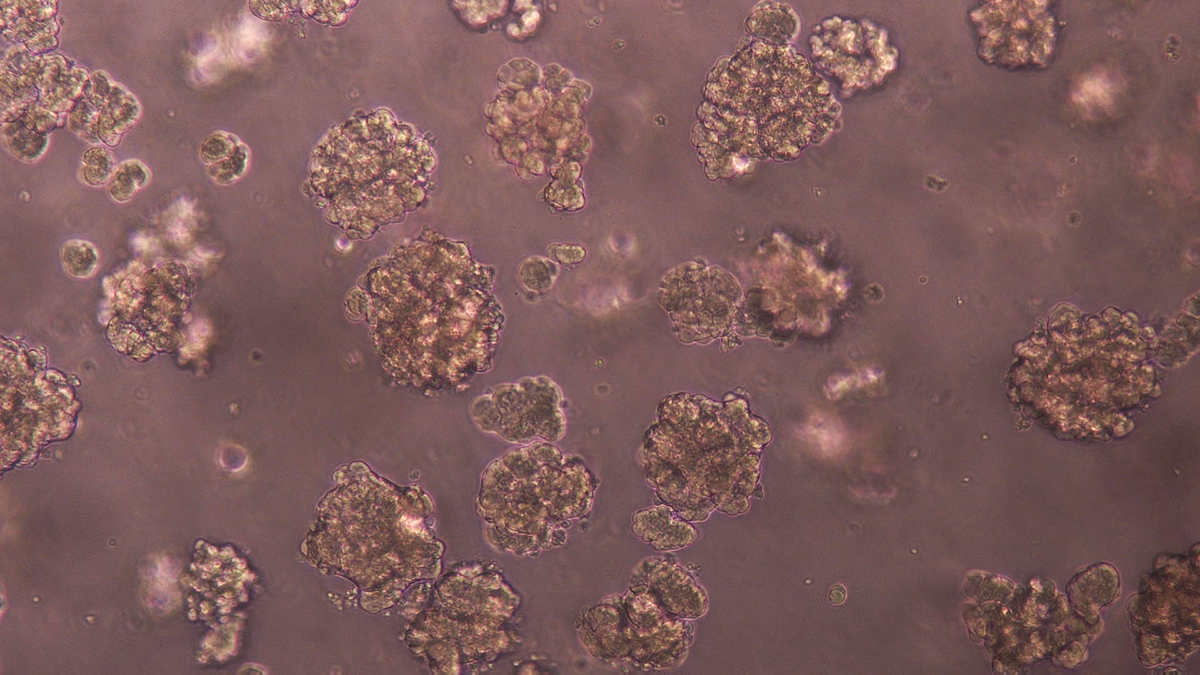

Supplement: Supplementary file 15 — Source data Fig. EV1 [file 44318_2024_328_MOESM15_ESM.zip › Extended Data Figure 1D. AT2_early_passages-2_Brightfield.jpg]

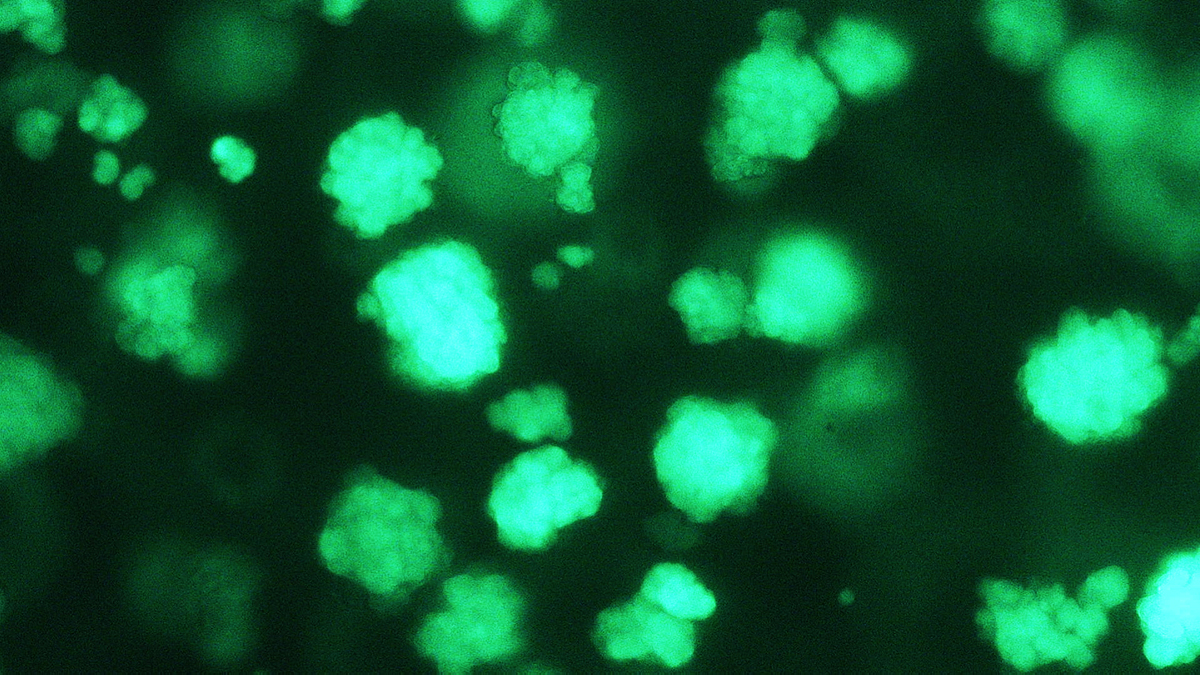

Supplement: Supplementary file 15 — Source data Fig. EV1 [file 44318_2024_328_MOESM15_ESM.zip › Extended Data Figure 1D. AT2_early_passages-2_SPC-GFP.jpg]

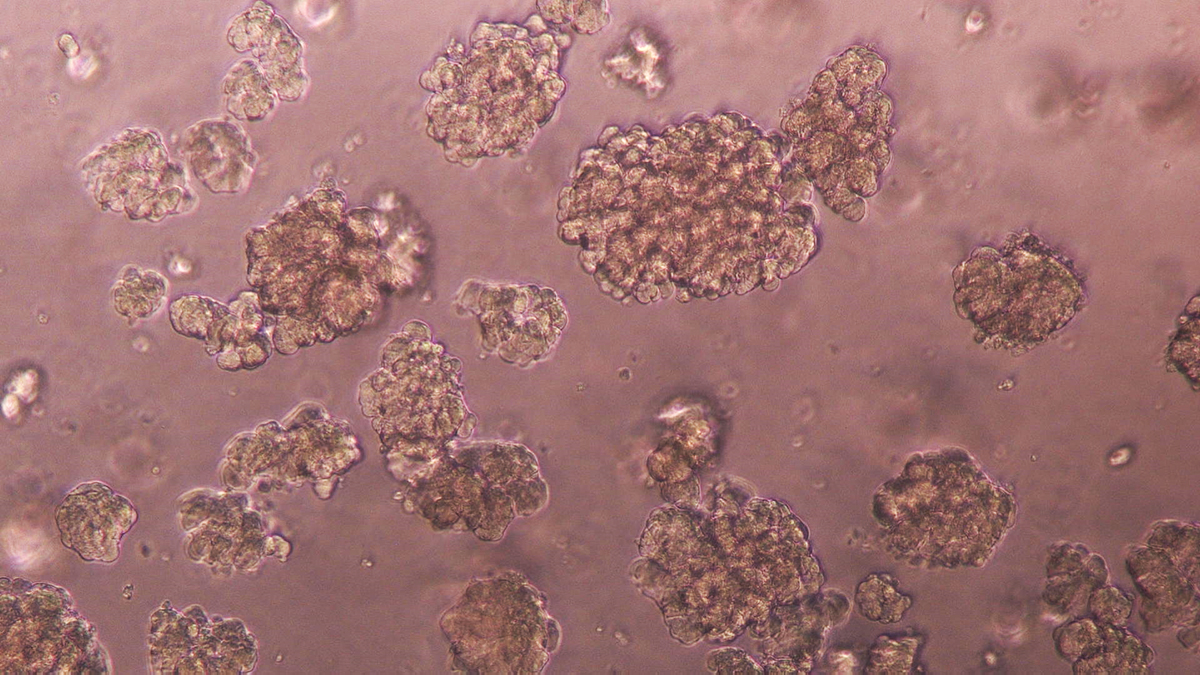

Supplement: Supplementary file 15 — Source data Fig. EV1 [file 44318_2024_328_MOESM15_ESM.zip › Extended Data Figure 1D. AT2_late_passages-1_Brightfield.jpg]

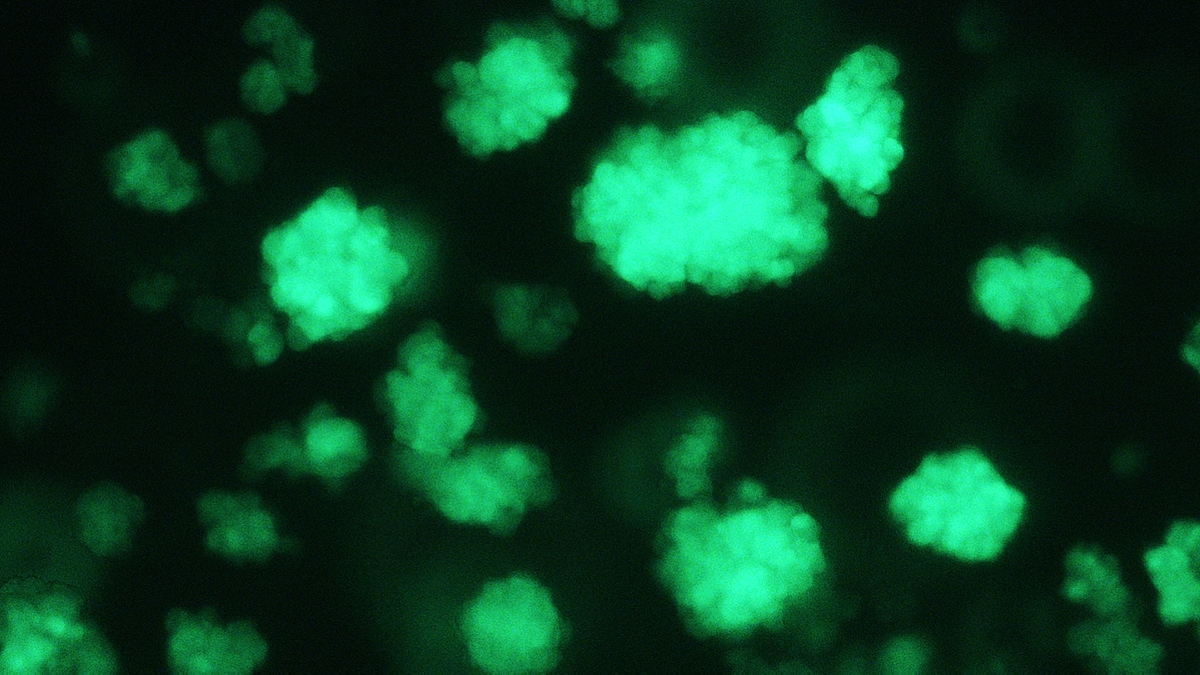

Supplement: Supplementary file 15 — Source data Fig. EV1 [file 44318_2024_328_MOESM15_ESM.zip › Extended Data Figure 1D. AT2_late_passages-1_SPC-GFP.jpg]

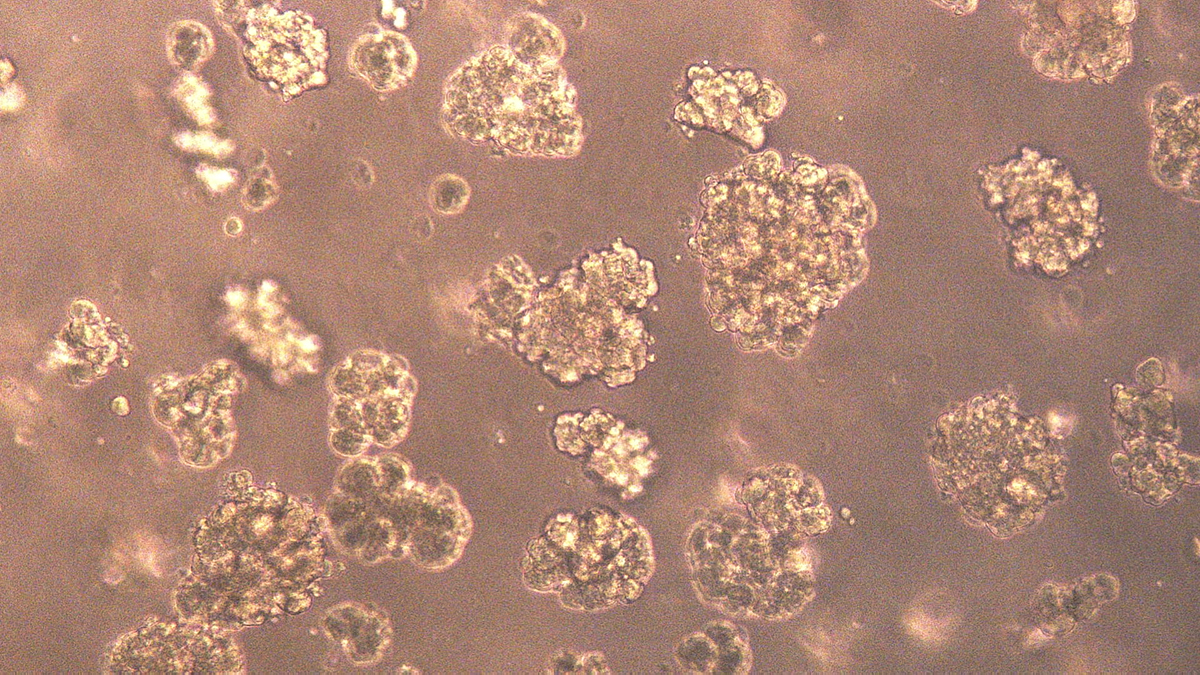

Supplement: Supplementary file 15 — Source data Fig. EV1 [file 44318_2024_328_MOESM15_ESM.zip › Extended Data Figure 1D. AT2_late_passages-2_Brightfield.jpg]

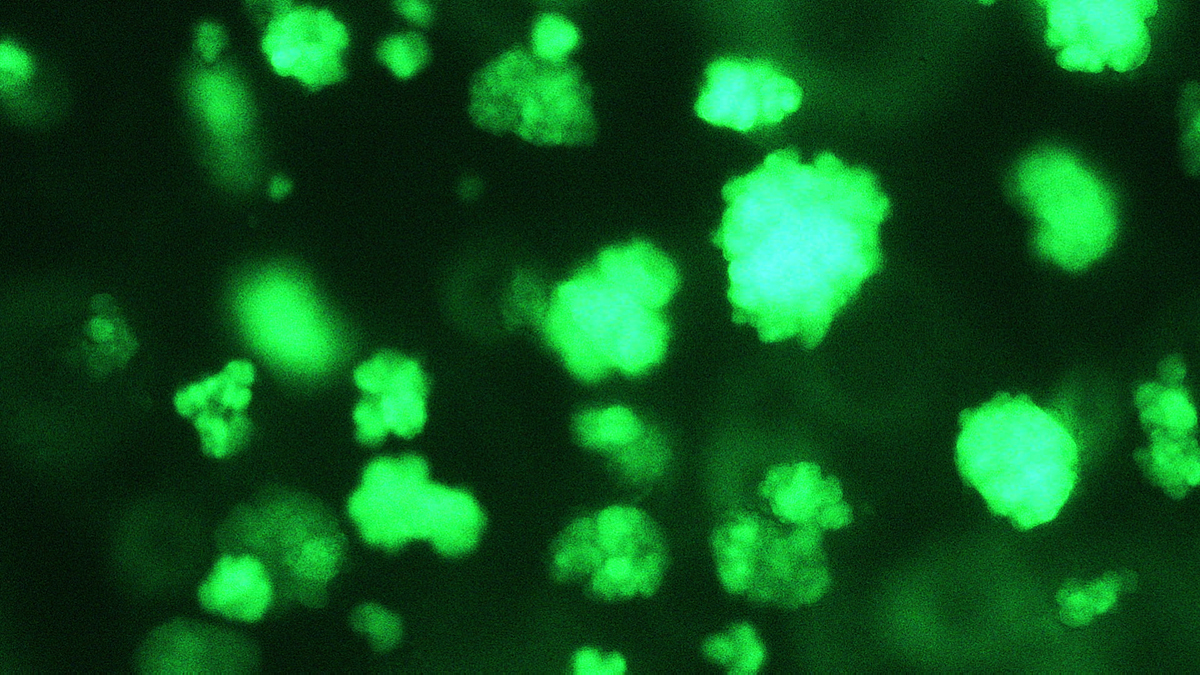

Supplement: Supplementary file 15 — Source data Fig. EV1 [file 44318_2024_328_MOESM15_ESM.zip › Extended Data Figure 1D. AT2_late_passages-2_SPC-GFP.jpg]

ACTB

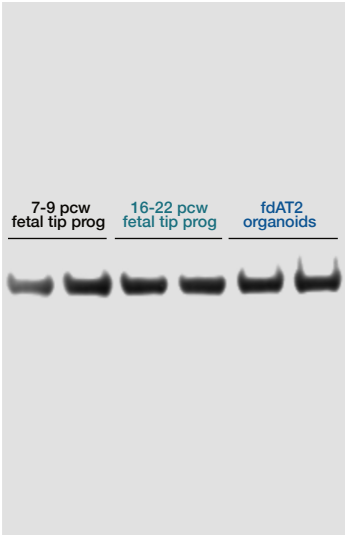

mature SFTPC

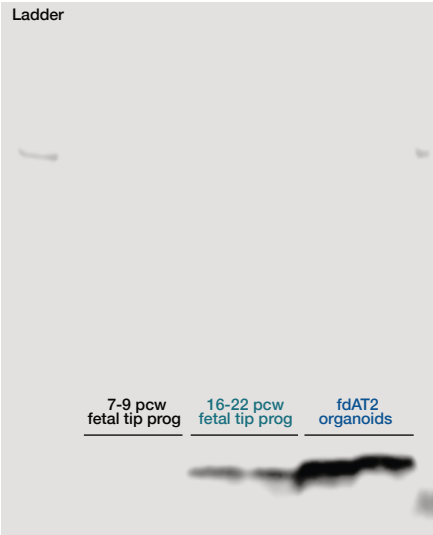

mature SFTPB

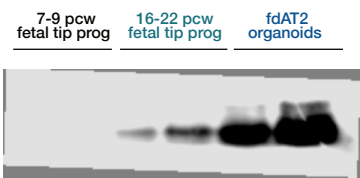

Supplement: Supplementary file 15 — Source data Fig. EV1 [file 44318_2024_328_MOESM15_ESM.zip › Extended Data Figure 1G. WB blot_mature surfactants in early-late-AT2 organoids.pdf]

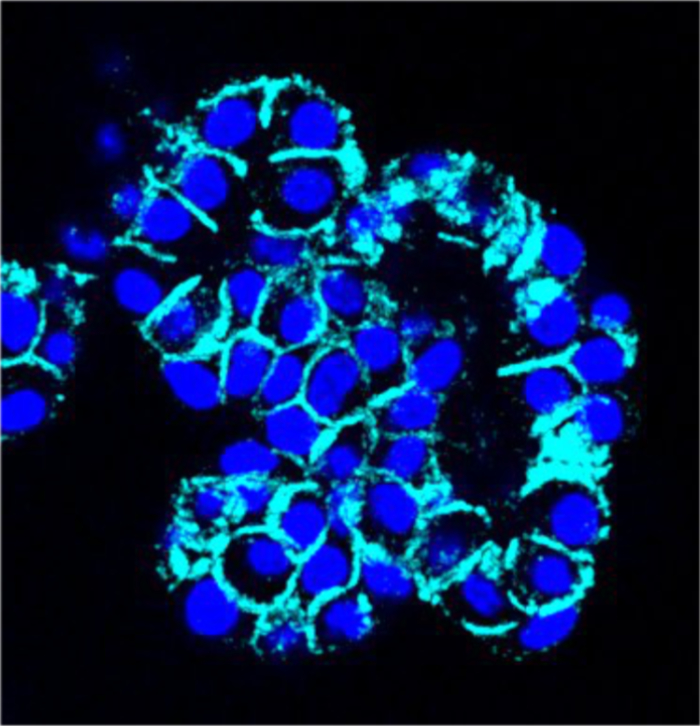

Supplement: Supplementary file 15 — Source data Fig. EV1 [file 44318_2024_328_MOESM15_ESM.zip › Extended Data Figure 1H. Sectred mature surfactant in fdAT2 organoid-1.jpg]

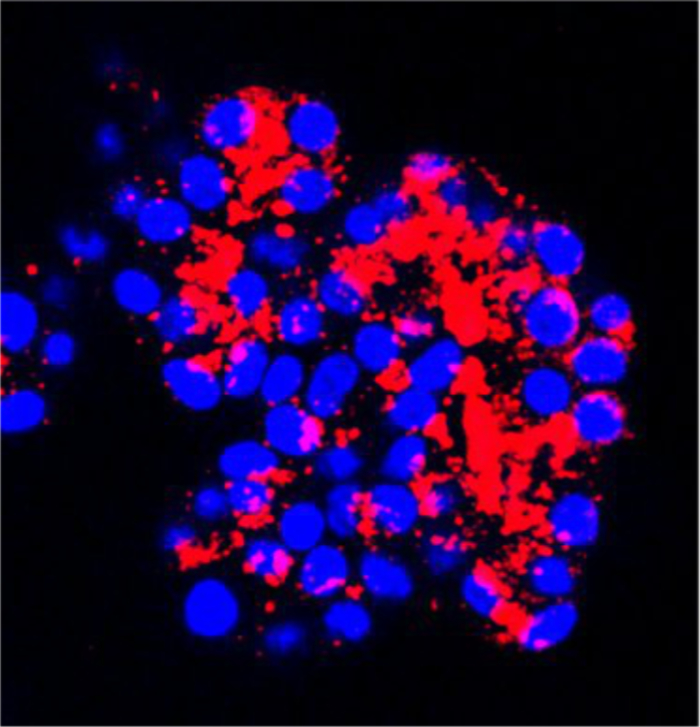

Supplement: Supplementary file 15 — Source data Fig. EV1 [file 44318_2024_328_MOESM15_ESM.zip › Extended Data Figure 1H. Sectred mature surfactant in fdAT2 organoid-2.jpg]

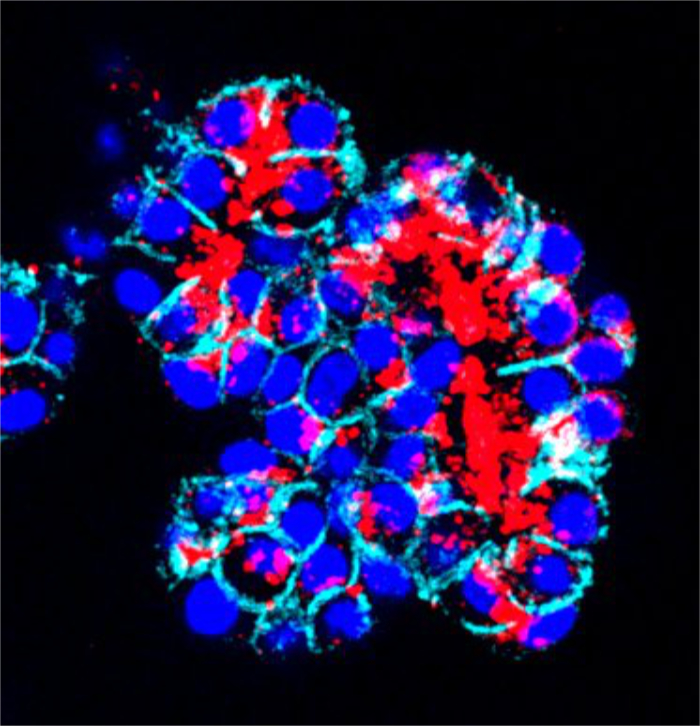

Supplement: Supplementary file 15 — Source data Fig. EV1 [file 44318_2024_328_MOESM15_ESM.zip › Extended Data Figure 1H. Sectred mature surfactant in fdAT2 organoid-3.jpg]

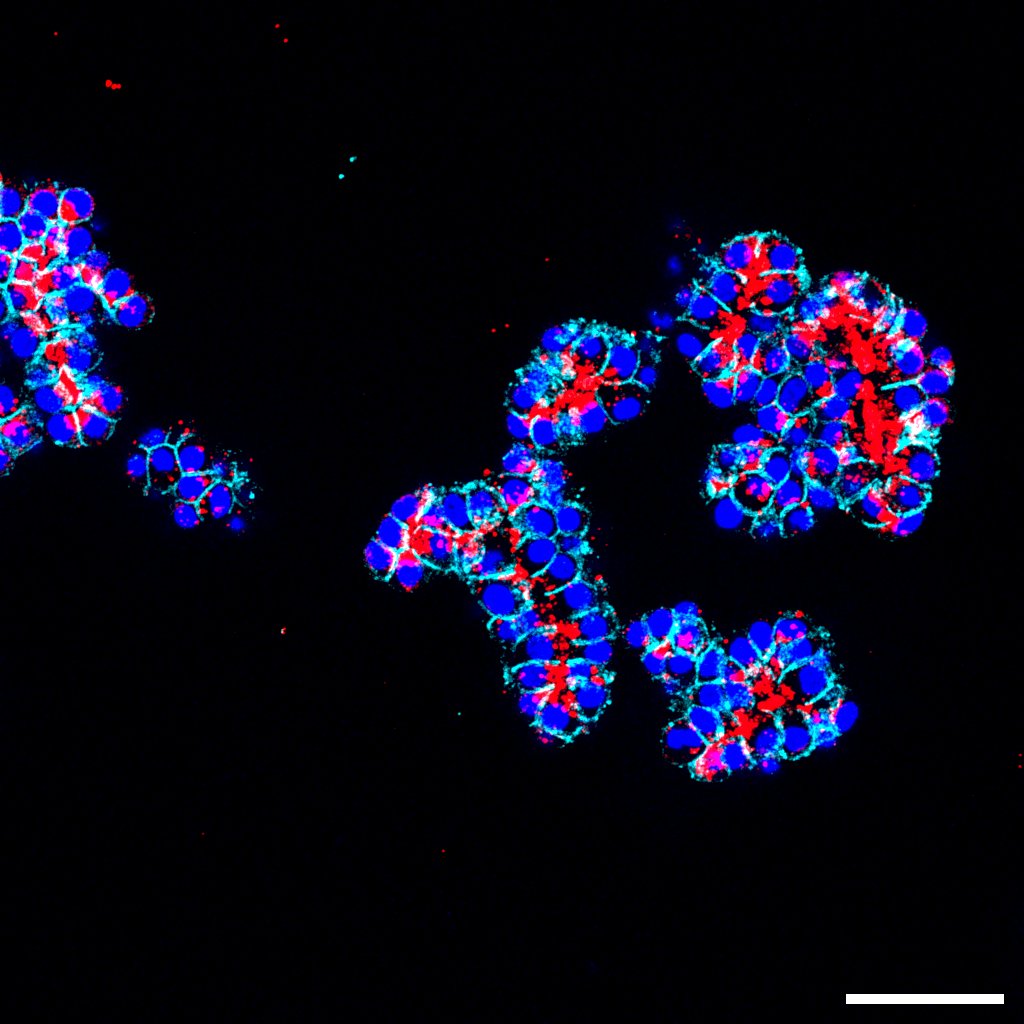

Supplement: Supplementary file 15 — Source data Fig. EV1 [file 44318_2024_328_MOESM15_ESM.zip › Extended Data Figure 1H. Sectred mature surfactant in fdAT2 organoid-4.jpg]

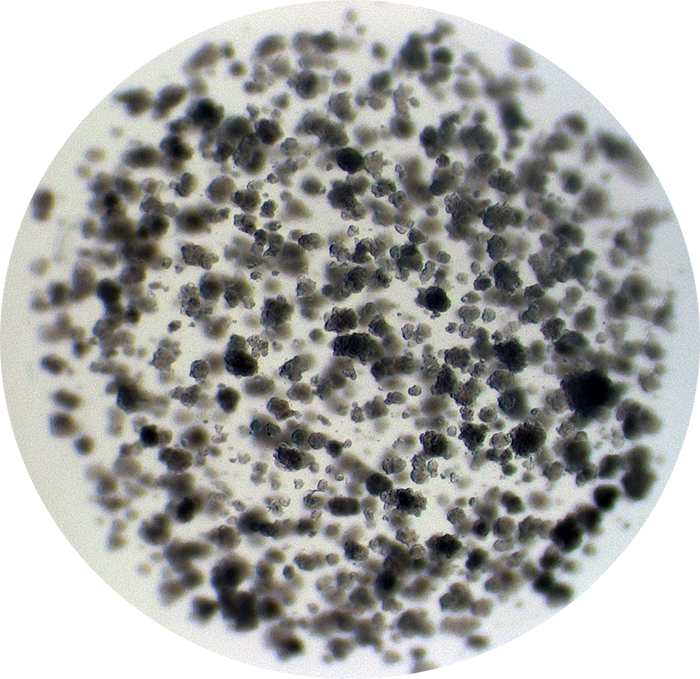

Supplement: Supplementary file 15 — Source data Fig. EV1 [file 44318_2024_328_MOESM15_ESM.zip › Extended Data Figure 1I-1.jpg]

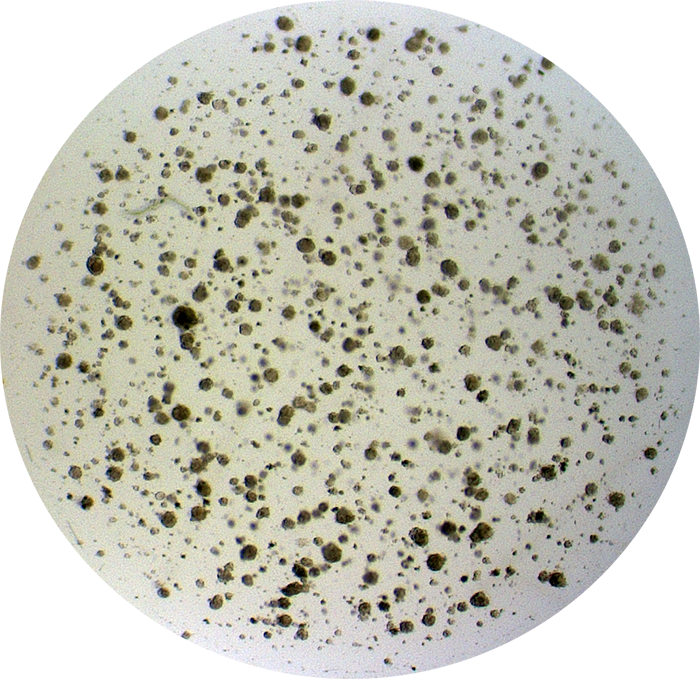

Supplement: Supplementary file 15 — Source data Fig. EV1 [file 44318_2024_328_MOESM15_ESM.zip › Extended Data Figure 1I-2.jpg]

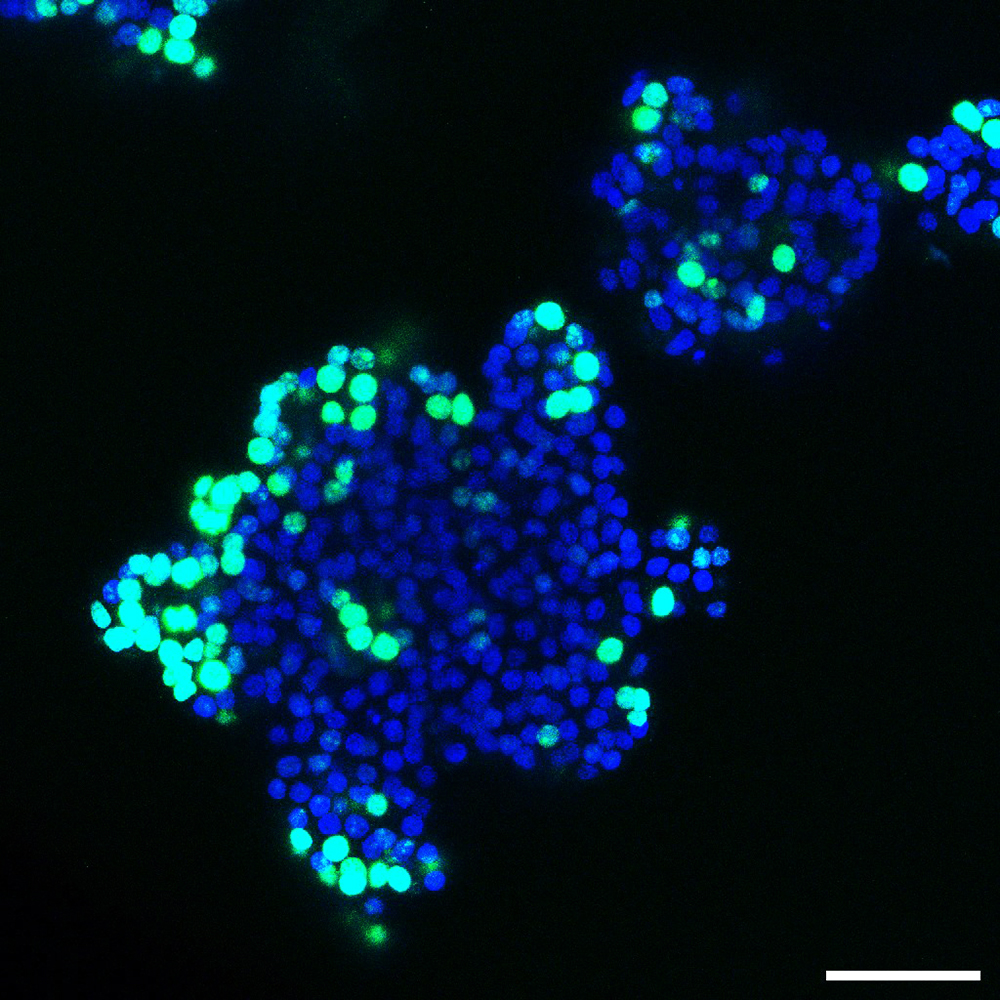

Supplement: Supplementary file 15 — Source data Fig. EV1 [file 44318_2024_328_MOESM15_ESM.zip › Extended Data Figure 1K. AT2 with FGF7-1.jpg]
