# Supplementary figures and images for: A novel human fetal lung-derived alveolar organoid model reveals mechanisms of surfactant protein C maturation relevant to interstitial lung disease (part 2 of 2)
Source: EMBO J. 2025 Jan 15;44(3):639–64. doi: 10.1038/s44318-024-00328-6 (PMC11790967; doi:10.1038/s44318-024-00328-6)

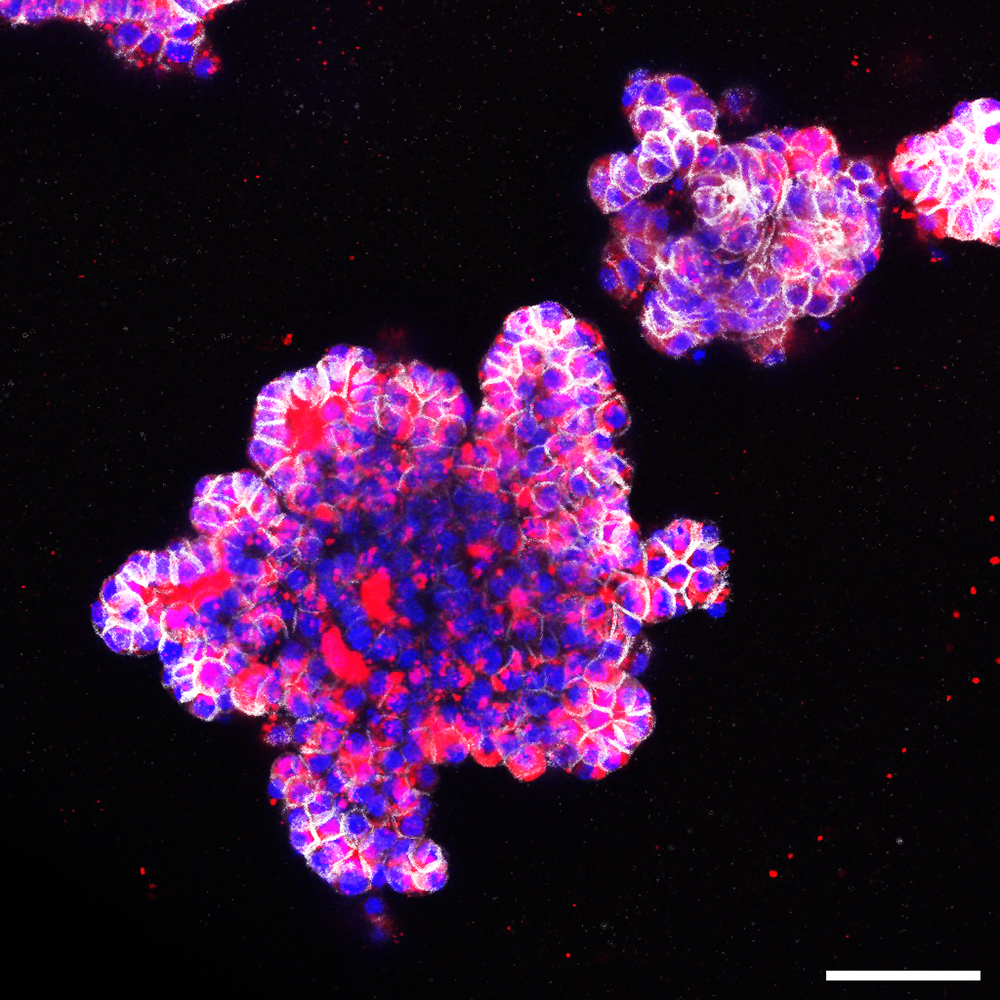

Supplement: Supplementary file 15 — Source data Fig. EV1 [file 44318_2024_328_MOESM15_ESM.zip › Extended Data Figure 1K. AT2 with FGF7-2.jpg]

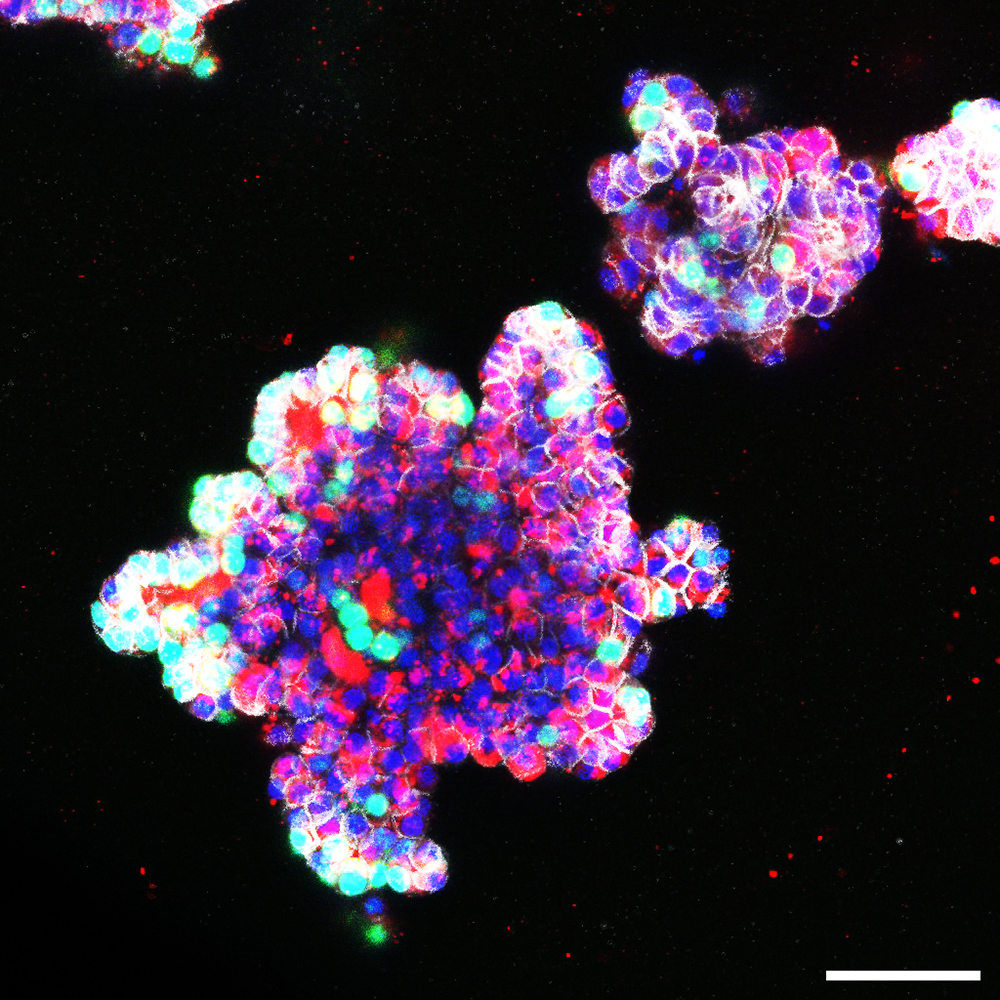

Supplement: Supplementary file 15 — Source data Fig. EV1 [file 44318_2024_328_MOESM15_ESM.zip › Extended Data Figure 1K. AT2 with FGF7-3.jpg]

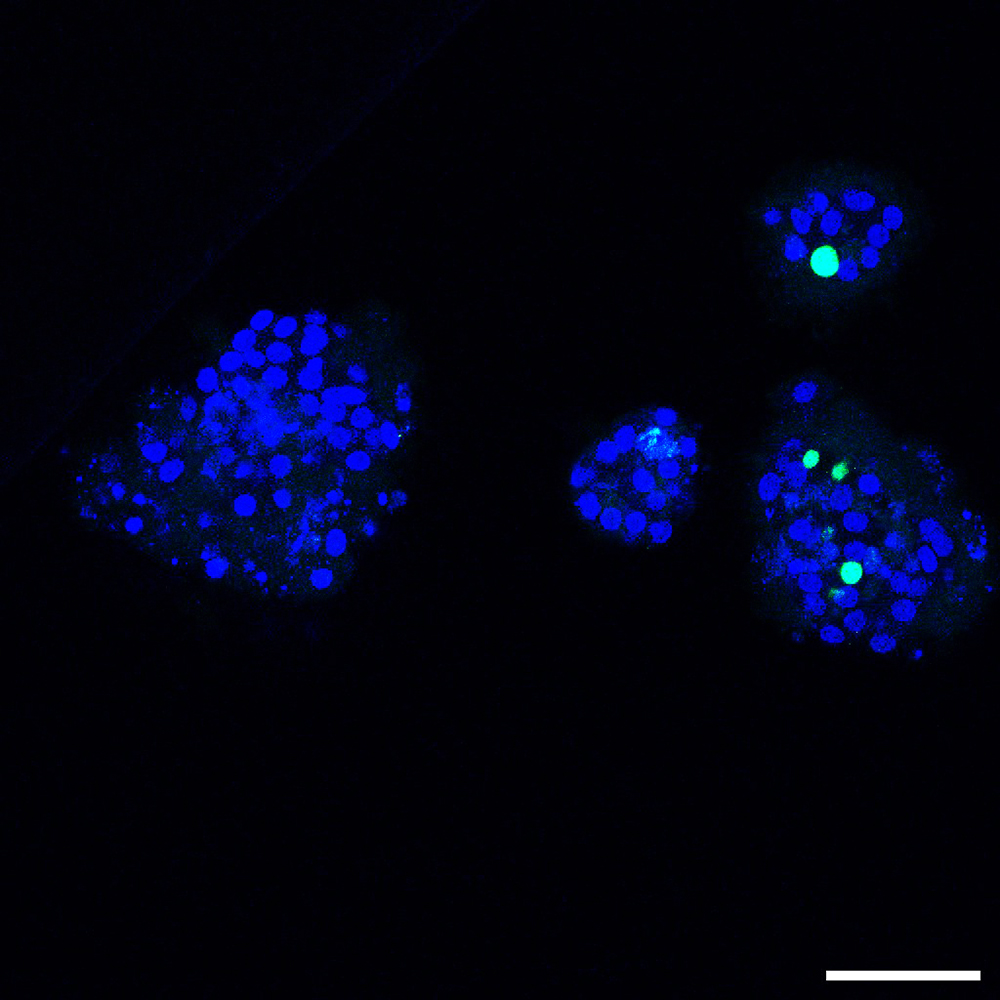

Supplement: Supplementary file 15 — Source data Fig. EV1 [file 44318_2024_328_MOESM15_ESM.zip › Extended Data Figure 1K. AT2 without FGF7-1.jpg]

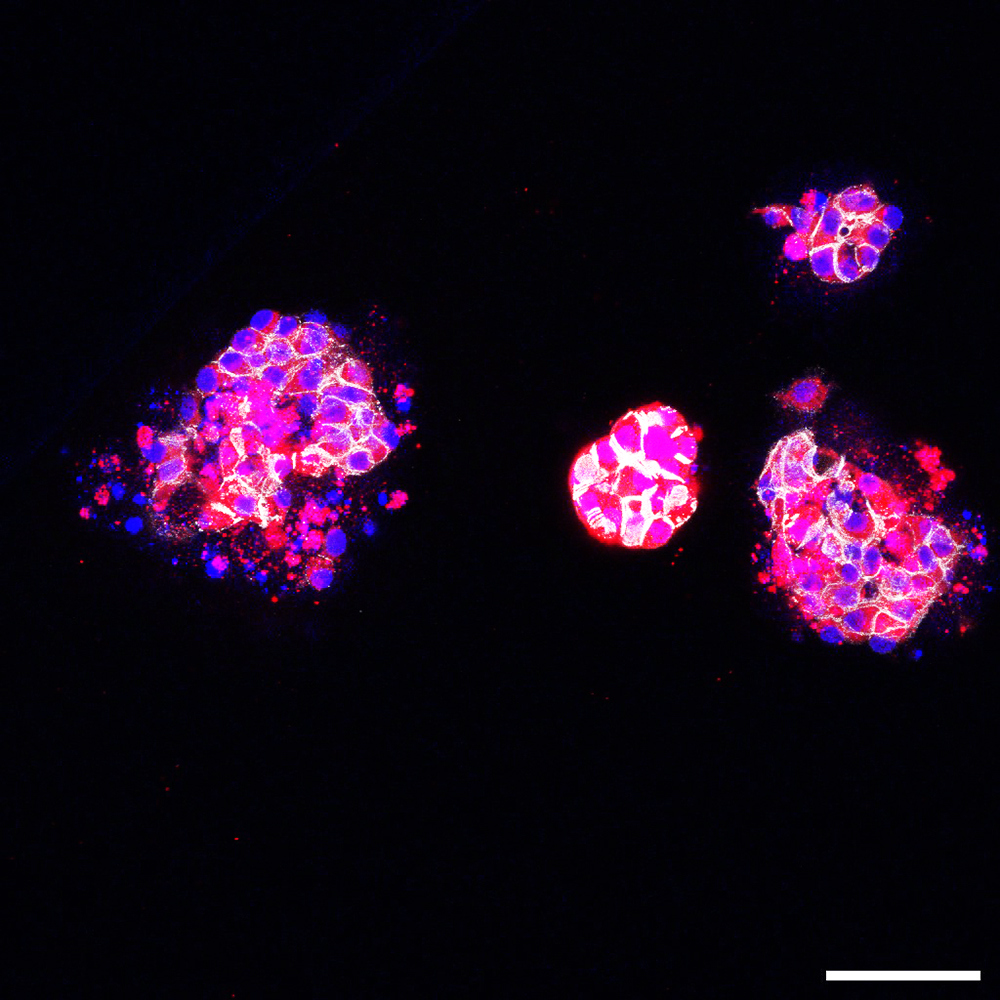

Supplement: Supplementary file 15 — Source data Fig. EV1 [file 44318_2024_328_MOESM15_ESM.zip › Extended Data Figure 1K. AT2 without FGF7-2.jpg]

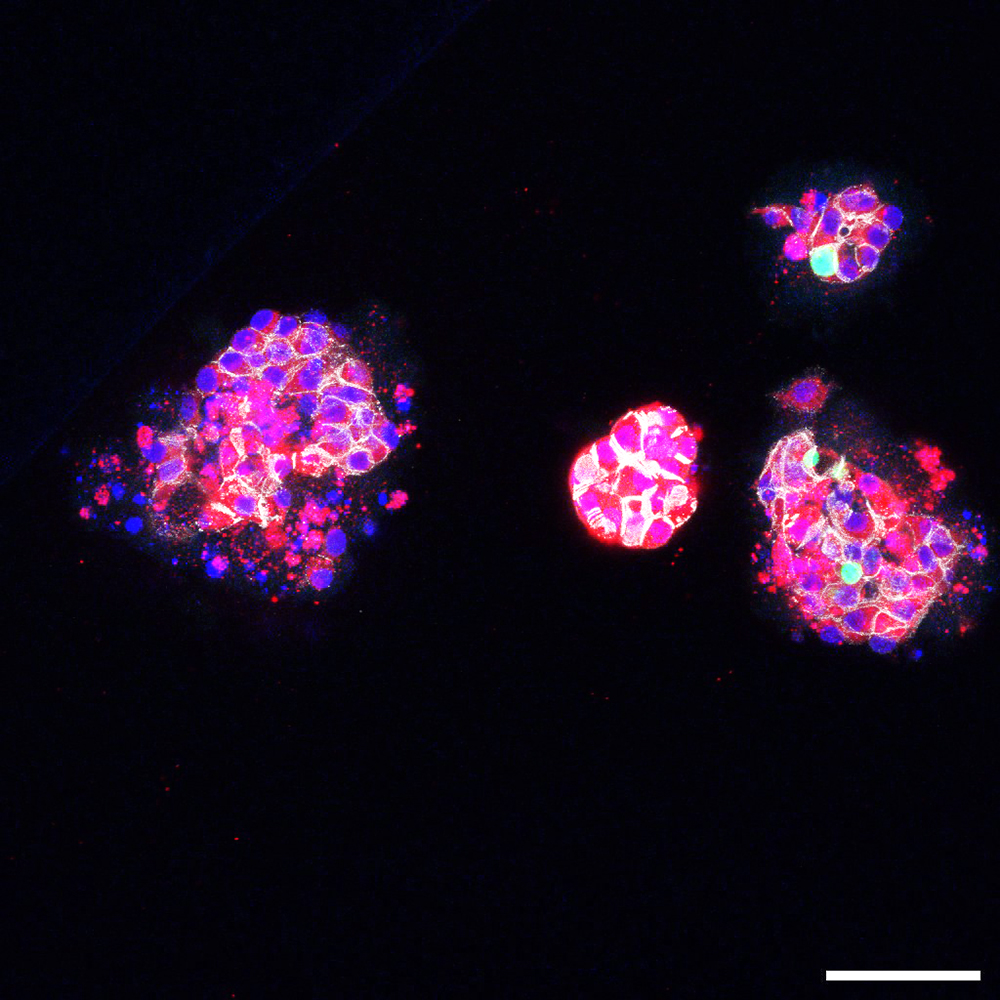

Supplement: Supplementary file 15 — Source data Fig. EV1 [file 44318_2024_328_MOESM15_ESM.zip › Extended Data Figure 1K. AT2 without FGF7-3.jpg]

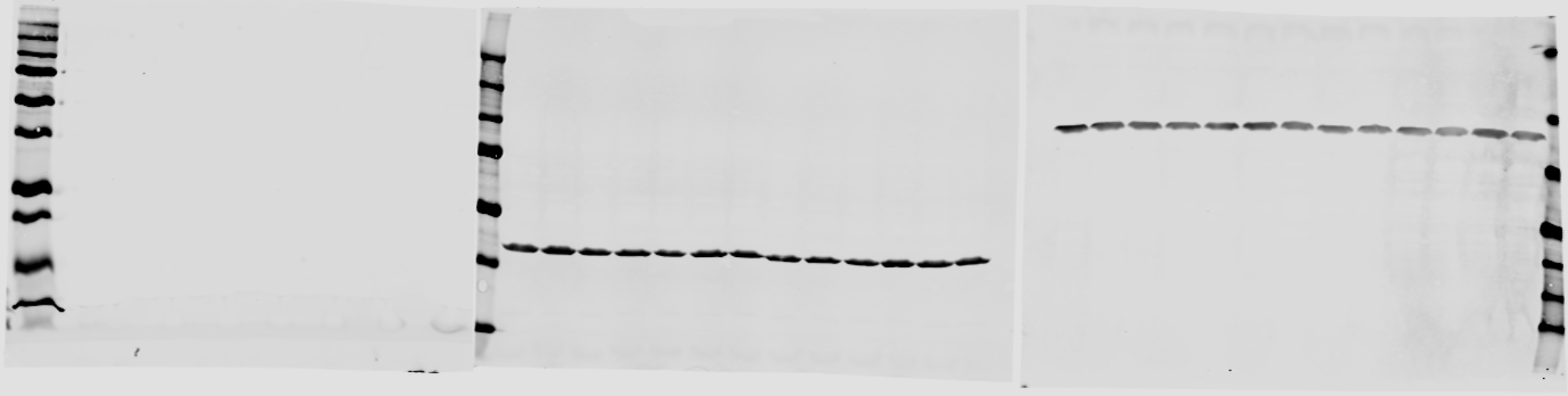

Supplement: Supplementary file 16 — Source data Fig. EV5 [file 44318_2024_328_MOESM16_ESM.zip › FigureEV5B_Image_ImageIDactin.tif]

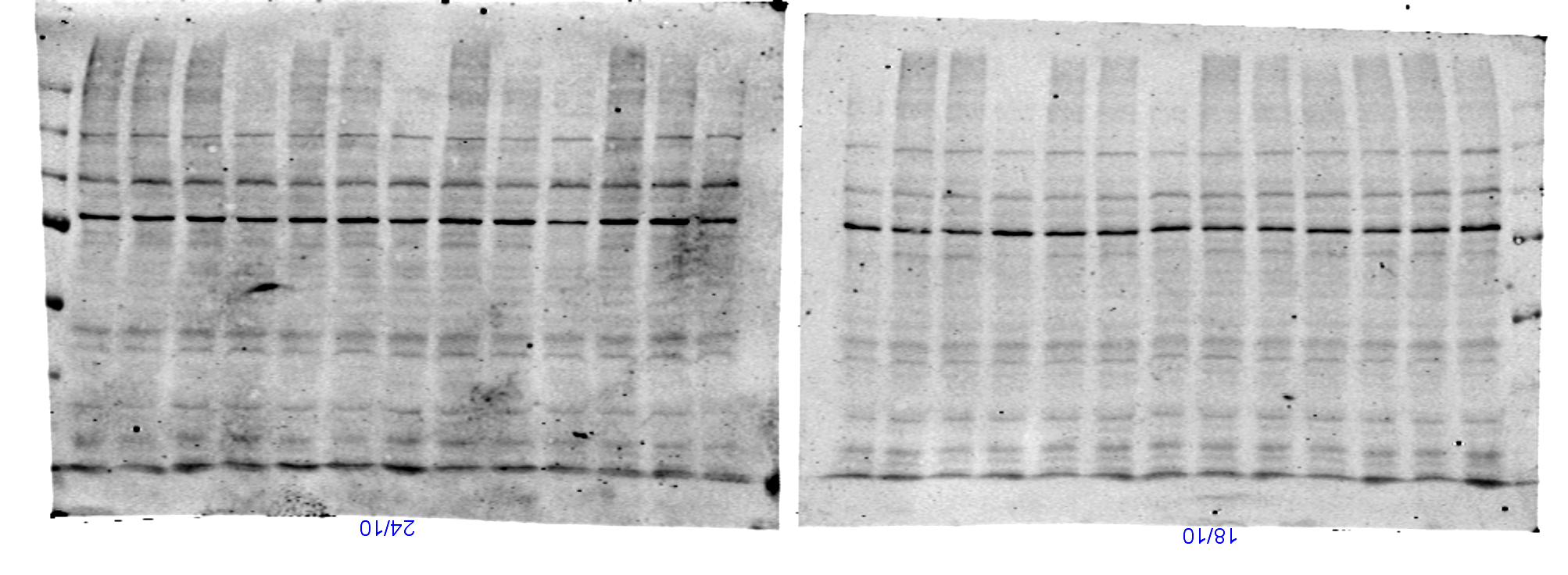

Supplement: Supplementary file 16 — Source data Fig. EV5 [file 44318_2024_328_MOESM16_ESM.zip › FigureEV5B_TAK-981 HeLa n=2_3.tif]

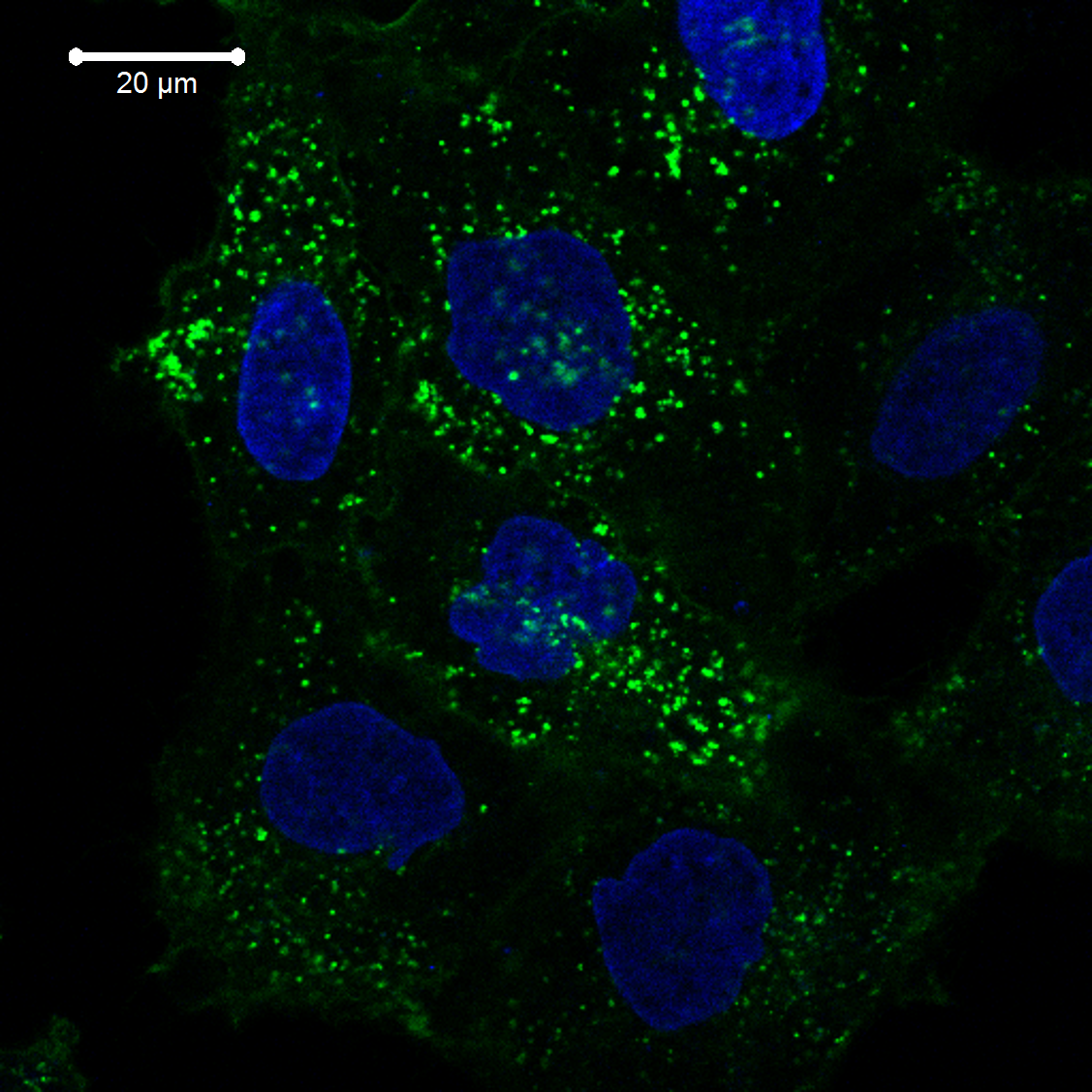

Supplement: Supplementary file 16 — Source data Fig. EV5 [file 44318_2024_328_MOESM16_ESM.zip › FigureEV5E_091123 ctrl 3zoom outczi.tif]

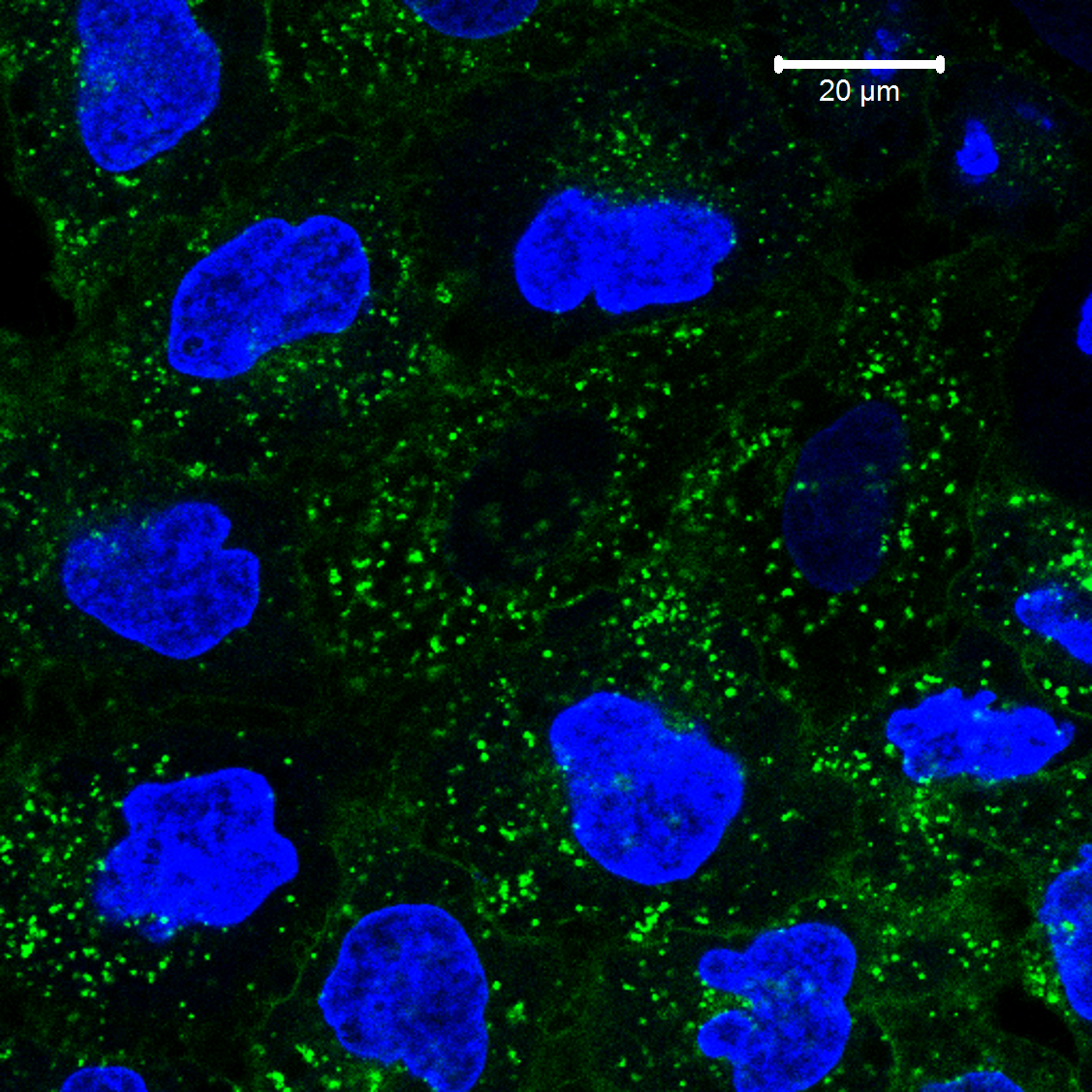

Supplement: Supplementary file 16 — Source data Fig. EV5 [file 44318_2024_328_MOESM16_ESM.zip › FigureEV5E_091123 TAK 4hrs zoom out.tif]

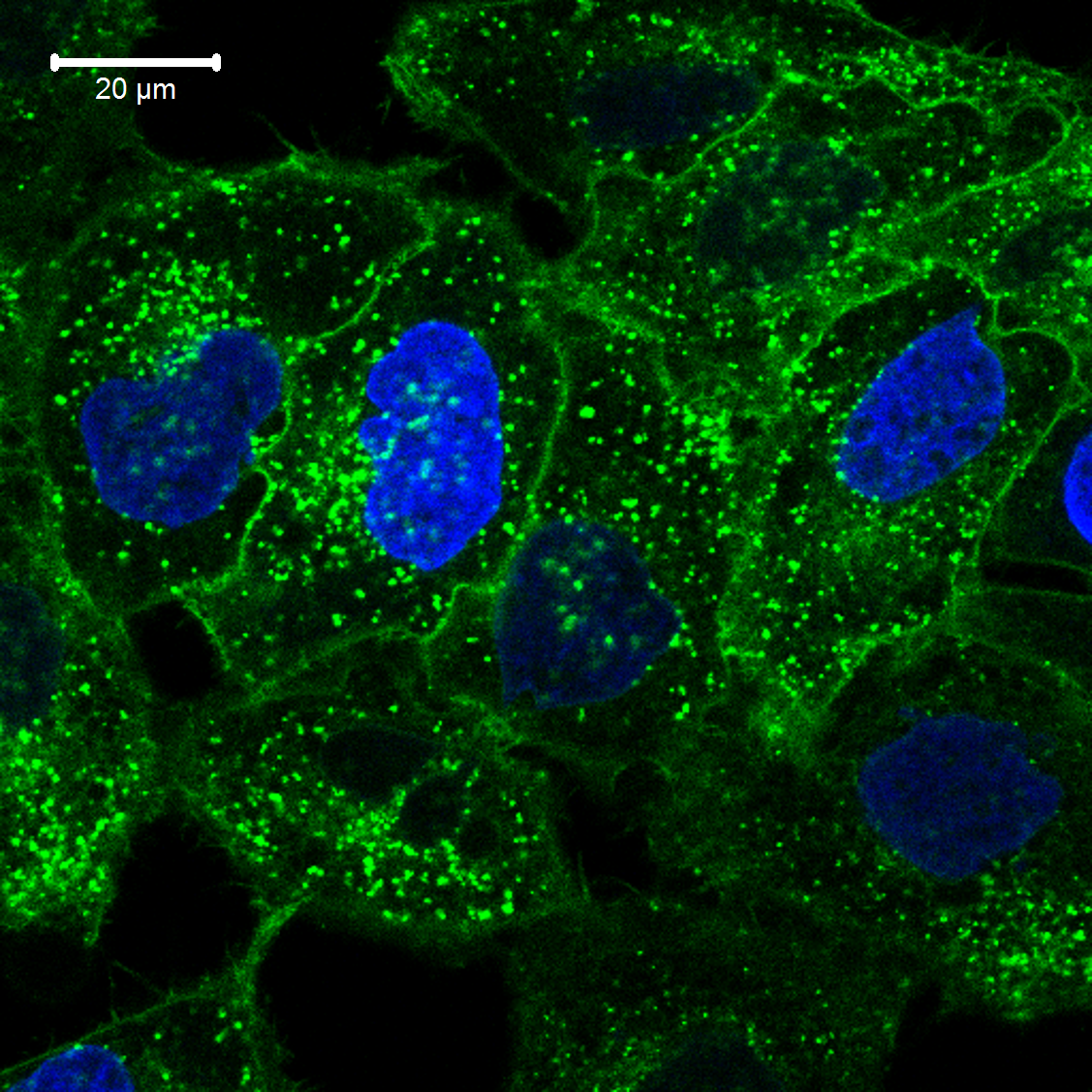

Supplement: Supplementary file 16 — Source data Fig. EV5 [file 44318_2024_328_MOESM16_ESM.zip › FigureEV5E_091123 TAK 20hrs zoom out.tif]

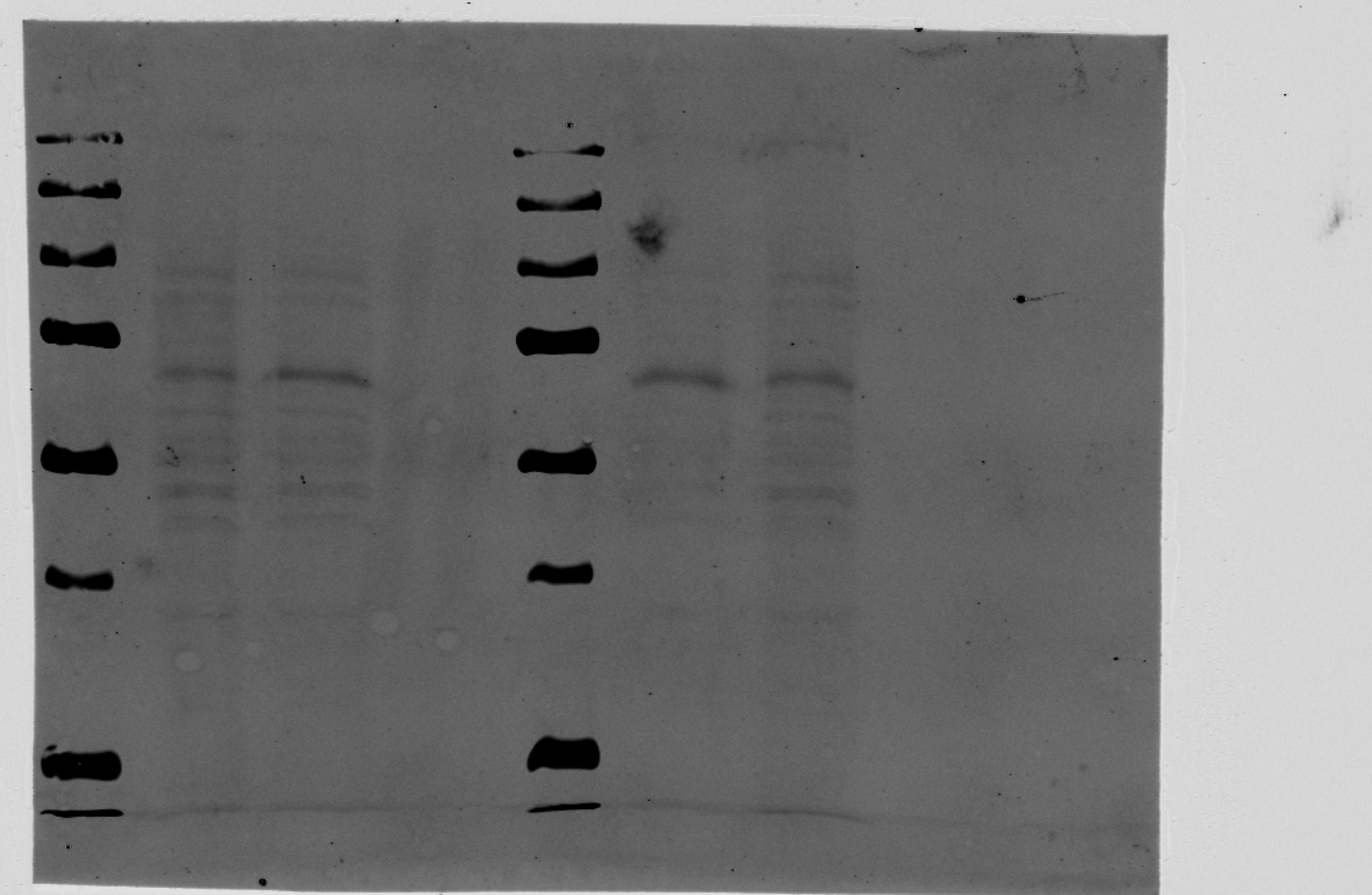

Supplement: Supplementary file 16 — Source data Fig. EV5 [file 44318_2024_328_MOESM16_ESM.zip › FigureEV5G_Image_0000007_01 (2).tif]

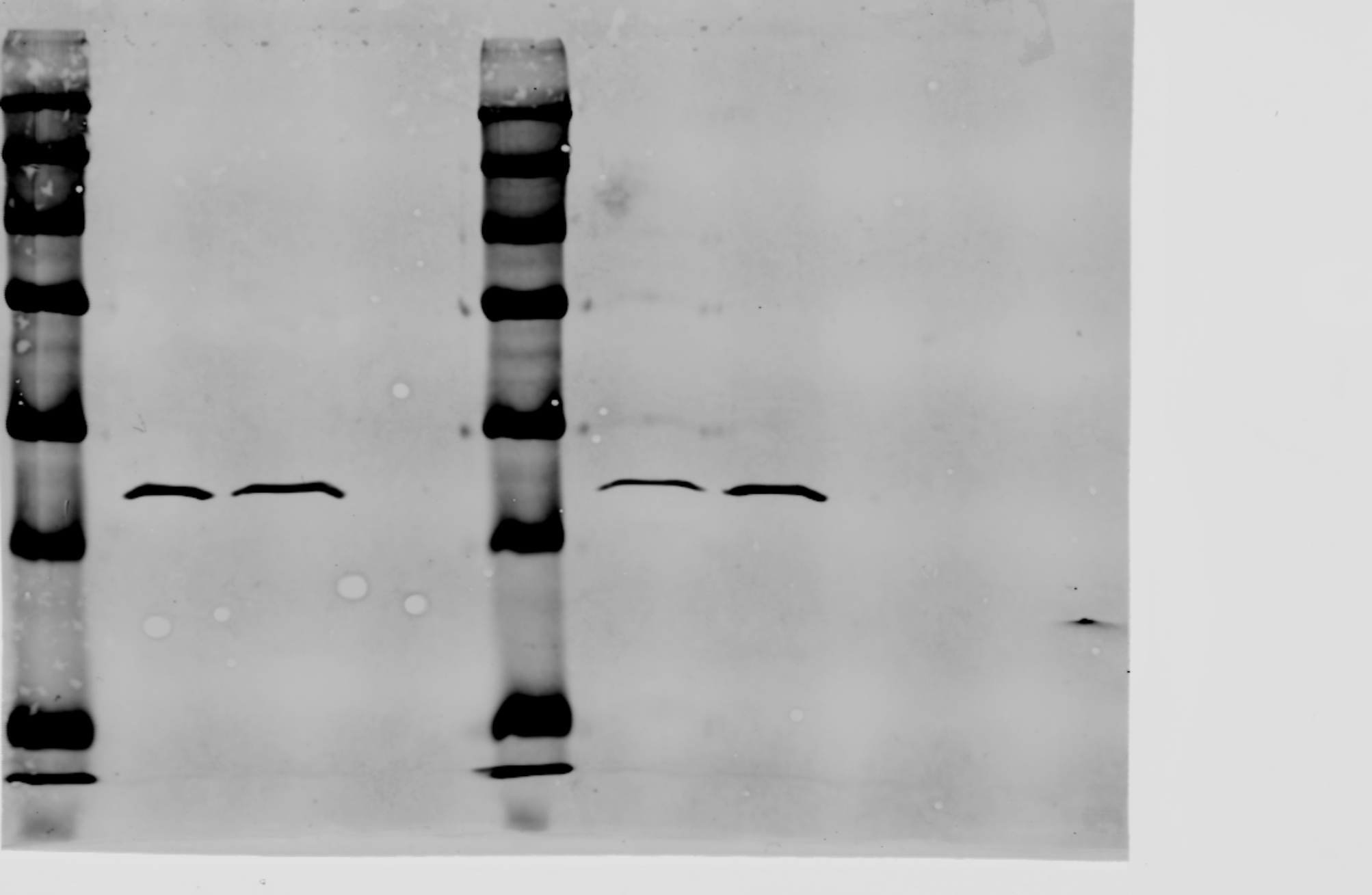

Supplement: Supplementary file 16 — Source data Fig. EV5 [file 44318_2024_328_MOESM16_ESM.zip › FigureEV5G_Image_actin (1).tif]

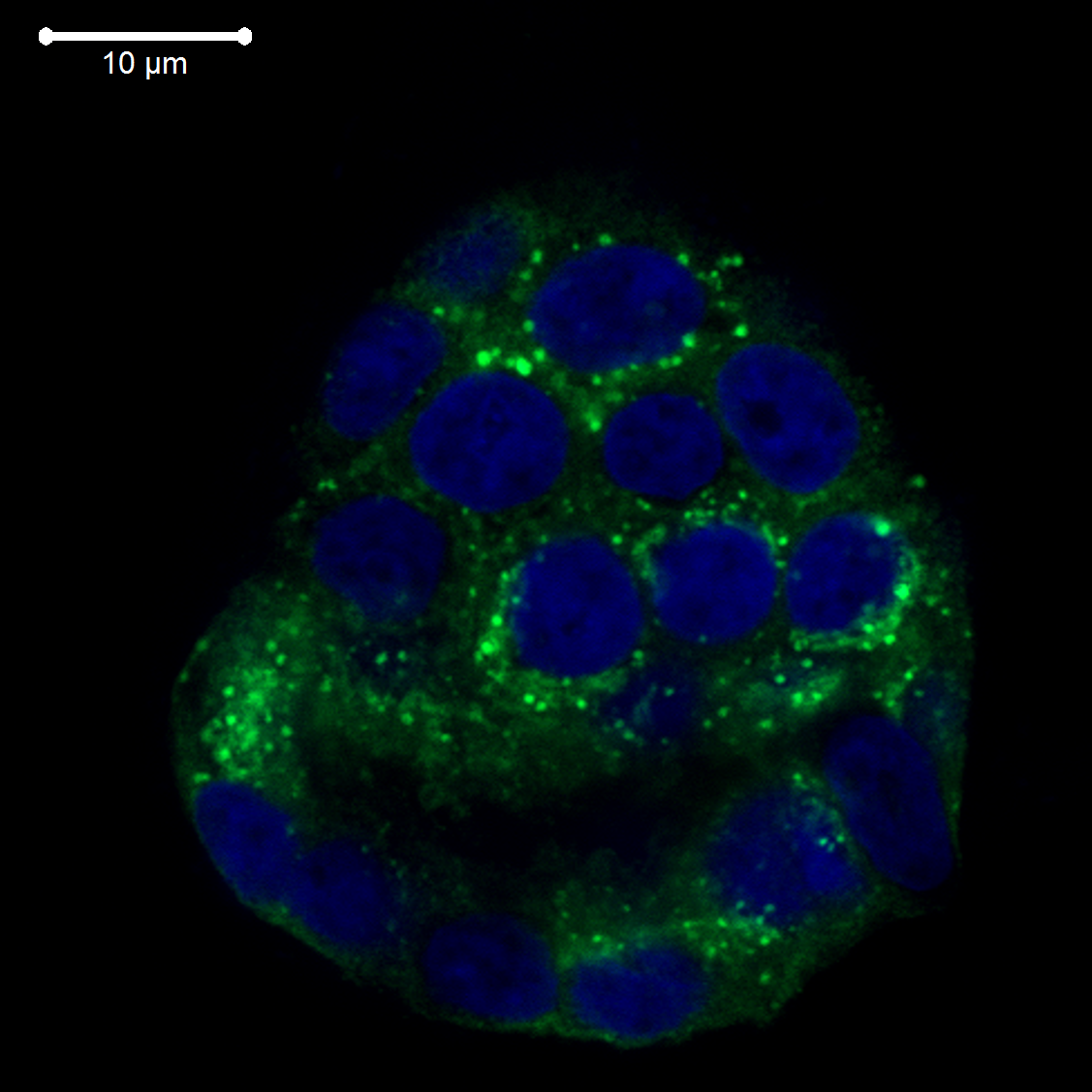

Supplement: Supplementary file 16 — Source data Fig. EV5 [file 44318_2024_328_MOESM16_ESM.zip › FigureEV5K_ 16011 ctrl 3_Airyscan Processing.tif]

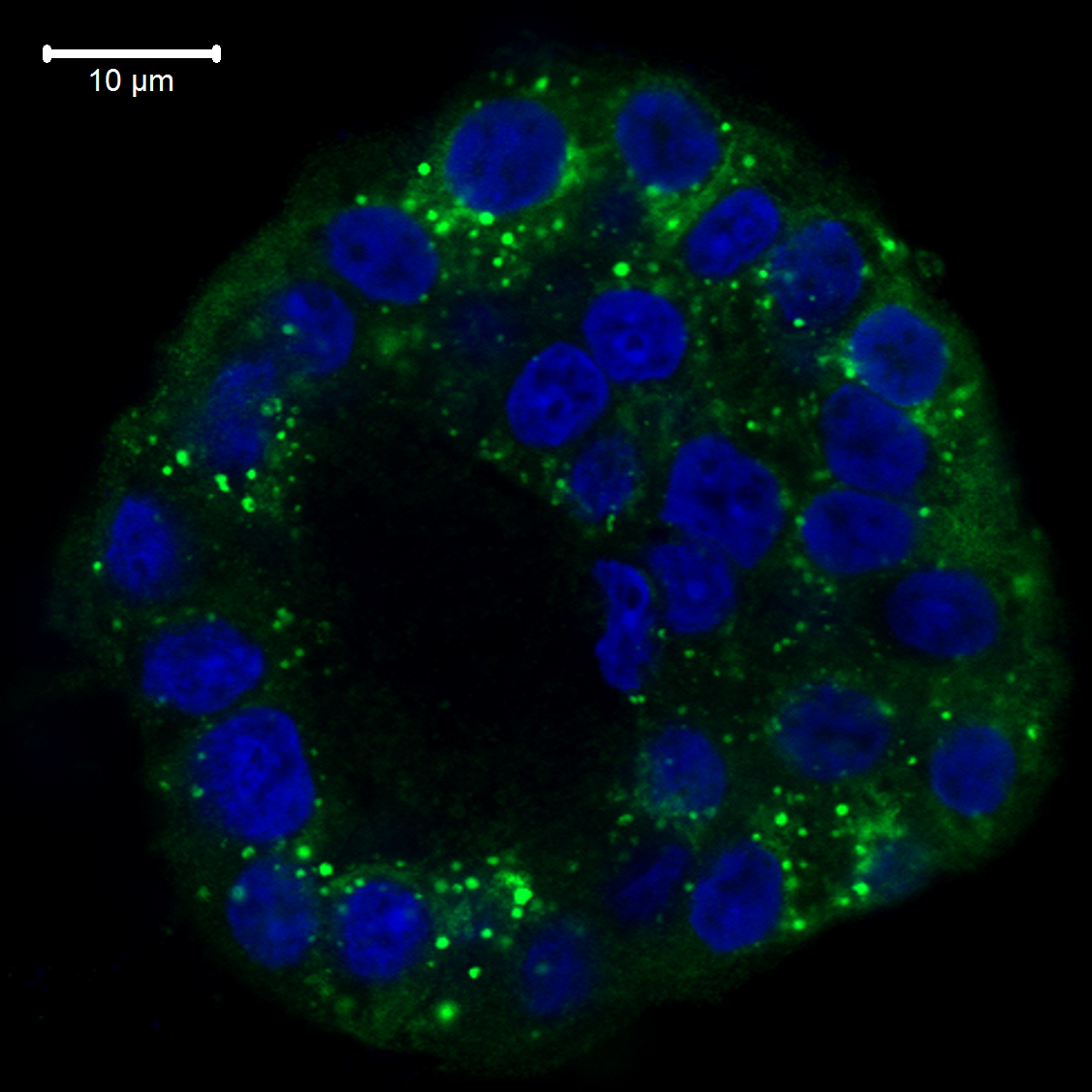

Supplement: Supplementary file 16 — Source data Fig. EV5 [file 44318_2024_328_MOESM16_ESM.zip › FigureEV5K_ 16011 TAK 4_Airyscan Processing.tif]

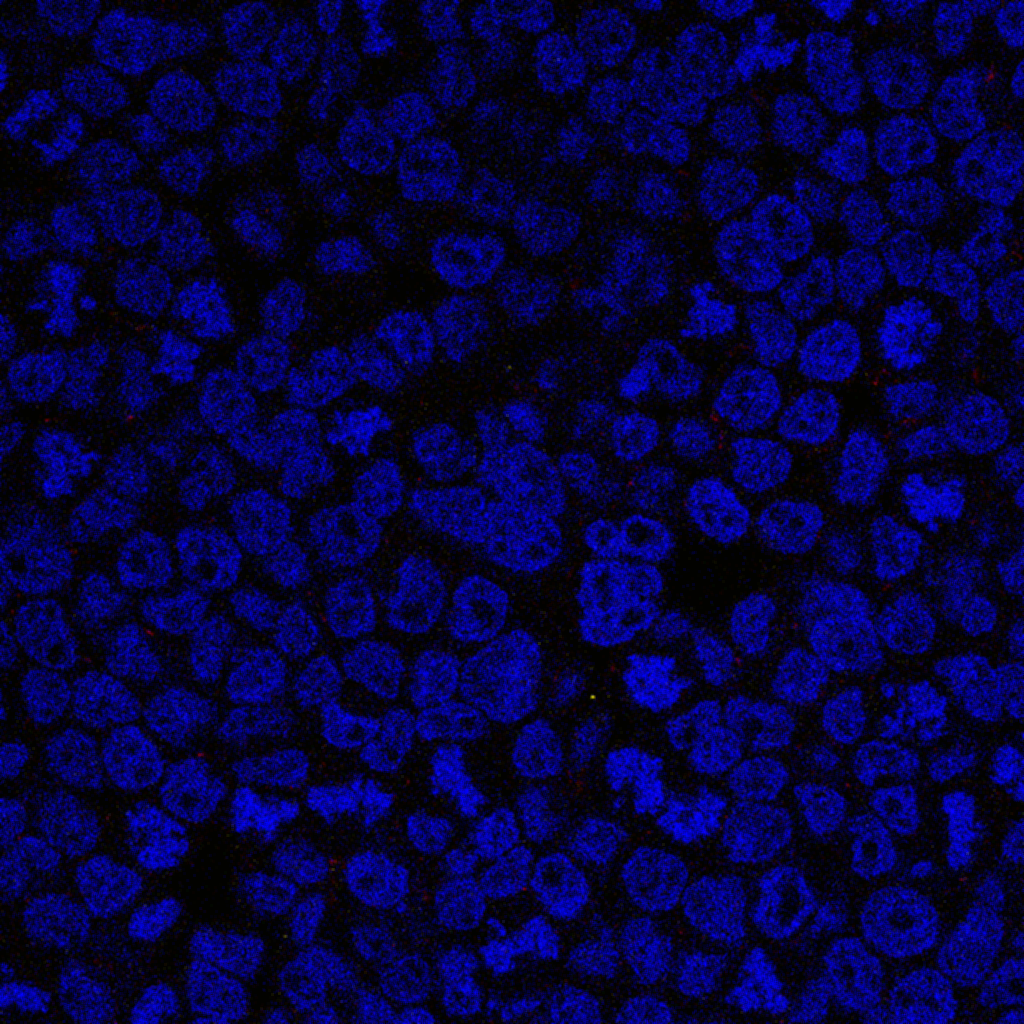

Supplement: Supplementary file 17 — Appendix Figure S1 Source Data [file 44318_2024_328_MOESM17_ESM.zip › HEK293T_Zo1-HTII-280-Ecad.tif]

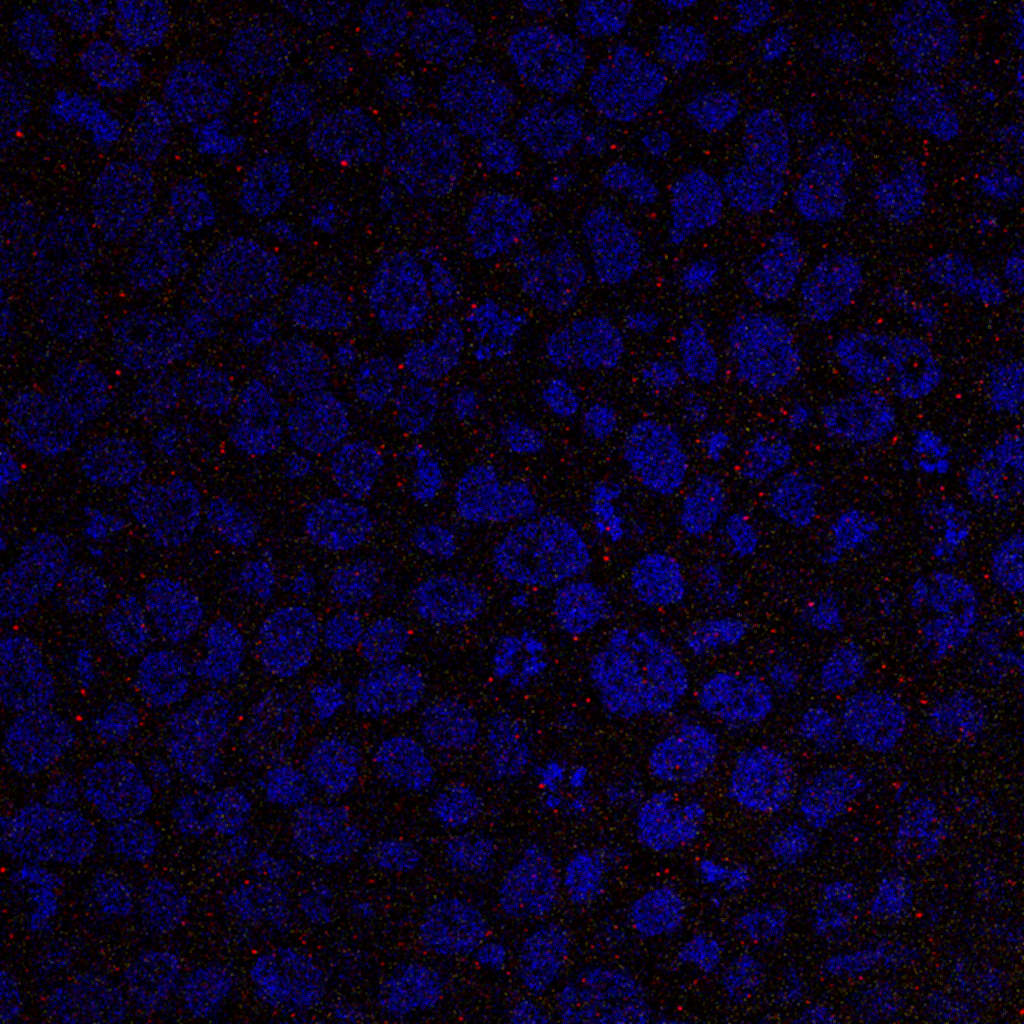

Supplement: Supplementary file 17 — Appendix Figure S1 Source Data [file 44318_2024_328_MOESM17_ESM.zip › HEK293T_mSFTPC-SOX9.tif]
