# Supplementary material for: A novel human fetal lung-derived alveolar organoid model reveals mechanisms of surfactant protein C maturation relevant to interstitial lung disease
Source: EMBO J. 2025 Jan 15;44(3):639–64. doi: 10.1038/s44318-024-00328-6 (PMC11790967; doi:10.1038/s44318-024-00328-6)
Supplement: Supplementary file 18 — Expanded View Figures [file 44318_2024_328_MOESM18_ESM.pdf]

Expanded View Figures

Figure EV1. Characterization of AT2 organoids.

(**A**) Derivation and establishment of fdAT2 organoids from lung tip progenitor cells (upper panel), or proximal airway progenitors (lower panel) of human fetal lungs at 22 pcw. Upper panel: The isolated tip progenitor cells were immediately transduced and selected based on *SFTPC*-GFP and EF1a-TagRFP after 48 h of transduction; *SFTPC*-GFP and EF1a-TagRFP reporter positive cells were efficiently expanded into fdAT2 organoids when grown in AT2 medium for 3 weeks. Lower panel: Proximal airway cells were immediately transduced with *SCGB3A2*-GFP, EF1a-TagRFP reporter lentivirus and the airway progenitor cells were selectively isolated by *SCGB3A2*-GFP after 48 h of transduction; *SCGB3A2*-GFP, *EF1a*-TagRFP reporter positive cells and expanded into small AT2-like organoids when grown in AT2 medium for 3 weeks, but efficiently formed airway organoids when grown in airway medium for the same period. Scale bar, 50 µm. (**B**) Size of organoids expanded from tip progenitors (*SFTPC*-GFP$^+$) or proximal airway progenitors (*SCGB3A2*-GFP$^+$) in AT2 medium was measured; mean ± SD, $n = 50$ organoids from one biological line (unpaired t test, two-tailed; $n = 1$ for each group); $P = 0.000000000000297$. (**C**) Expression of mature SFTPC protein in organoids expanded from tip progenitors or airway progenitors in AT2 medium. DAPI, nuclei. Scale bar, 50 µm. (**D**) Cultured fdAT2 organoids at early and late passages, stably expressing *SFTPC* promoter-driven GFP (*SFTPC*-GFP). Two independent lines of AT2 organoids at P3 and P17, and P4 and P20. Scale bar, 50 µm. (**E**) RT-qPCR analysis of alveolar type 2 cell lineage markers, *NKX2.1*, *SFTPC*, *ABCA3*, and *LAMP3*, in 7–9 pcw and 16–22 pcw tip progenitor organoids, and fdAT2 organoids at P12, P20, and P21. Data were normalized to 7–9 pcw tip organoids; mean ± SD, $n = 3$ biologically independent organoid lines (one-way ANOVA with Tukey multiple comparison post-test). (**F**) Additional electron microscopy showing the presence of lamellar bodies with characteristic concentric lamellar membranes within the cytosol. Scale bar, 1 µm. (**G**) Immunoblot of mature forms of SFTPC and SFTPB in human fetal lung tip progenitor-derived organoids at 7–9 pcw and 16–22 pcw, respectively, and the fdAT2 organoids. Two biologically independent organoid lines were used. (**H**) FdAT2 organoids were immunostained for E-cadherin (cyan) and mature SFTPC (red). Mature SFTPC is observed within the cells packaged into lamellar bodies and secreted into the lumen of the organoids. The lumen border is labeled by the dotted line. Arrowheads indicate cells without mature SFTPC expression on the merged panel. Scale bar, 50 µm. (**I**) FdAT2 organoids were cultured in AT2 medium for 2 weeks, in the presence (control) or absence of FGF7 (-FGF7) and AT2 lineage markers were measured by RT-qPCR (**J**) after 7 and 14 days of culture. Data were normalized to AT2 organoids cultured in the AT2 medium containing FGF7 (control); mean ± SD, $n = 3$ biologically independent organoid lines (one-way ANOVA with Tukey multiple comparison post-test). (**K**) Mature SFTPC protein expression was visualized with a proliferation marker, KI67, and E-cadherin by immunofluorescence staining, at 14 days of culture. DAPI, nuclei. Scale bar, 50 µm. Source data are available online for this figure.

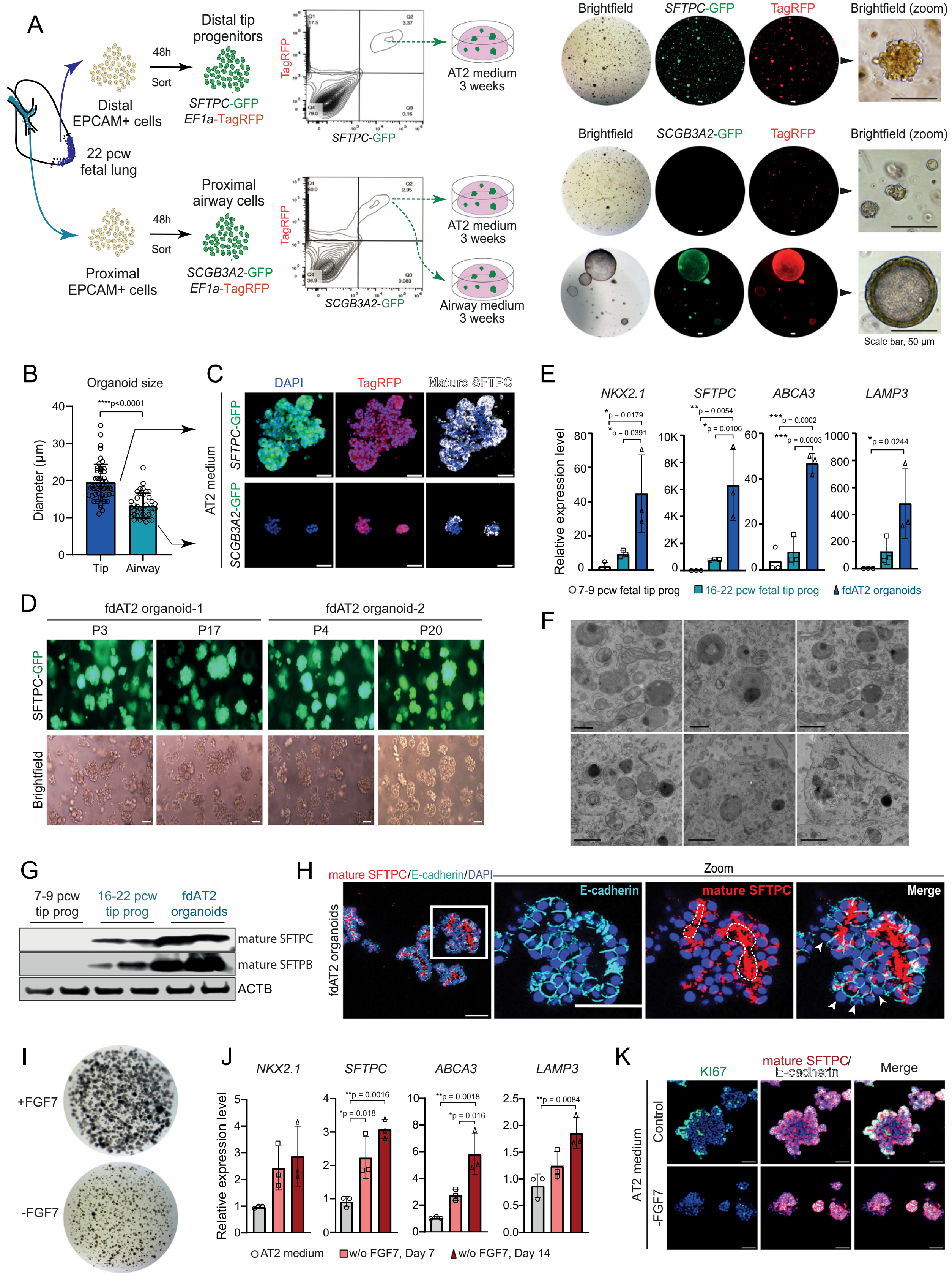
A
Distal tip progenitors
48h
Sort
Distal EPCAM+ cells
SFTPC-GFP
EF1a-TagRFP
22 pcw fetal lung
Proximal airway cells
Proximal EPCAM+ cells
SCGB3A2-GFP
TagRFP
AT2 medium 3 weeks
Airway medium 3 weeks
Brightfield
TagRFP
Brightfield (zoom)
Scale bar, 50 μm
B
Organoid size
****p<0.0001
Diameter (μm)
Tip
Airway
C
DAPI
Mature SFTPC
AT2 medium
E
NKX2.1
SFTPC
ABCA3
LAMP3
Relative expression level
*p = 0.0179
*p = 0.0391
**p = 0.0054
*p = 0.0106
***p = 0.0002
***p = 0.0003
*p = 0.0244
7-9 pcw fetal tip prog
16-22 pcw fetal tip prog
fdAT2 organoids
D
fdAT2 organoid-1
fdAT2 organoid-2
P3
P17
P4
P20
F
G
7-9 pcw tip prog
16-22 pcw tip prog
mature SFTPC
mature SFTPB
ACTB
H
mature SFTPC/E-cadherin/DAPI
Zoom
E-cadherin
Merge
fdAT2 organoids
I
+FGF7
-FGF7
J
*p = 0.018
**p = 0.0016
**p = 0.0018
*p = 0.016
**p = 0.0084
AT2 medium
w/o FGF7, Day 7
w/o FGF7, Day 14
K
KI67
Control

 © The Author(s)

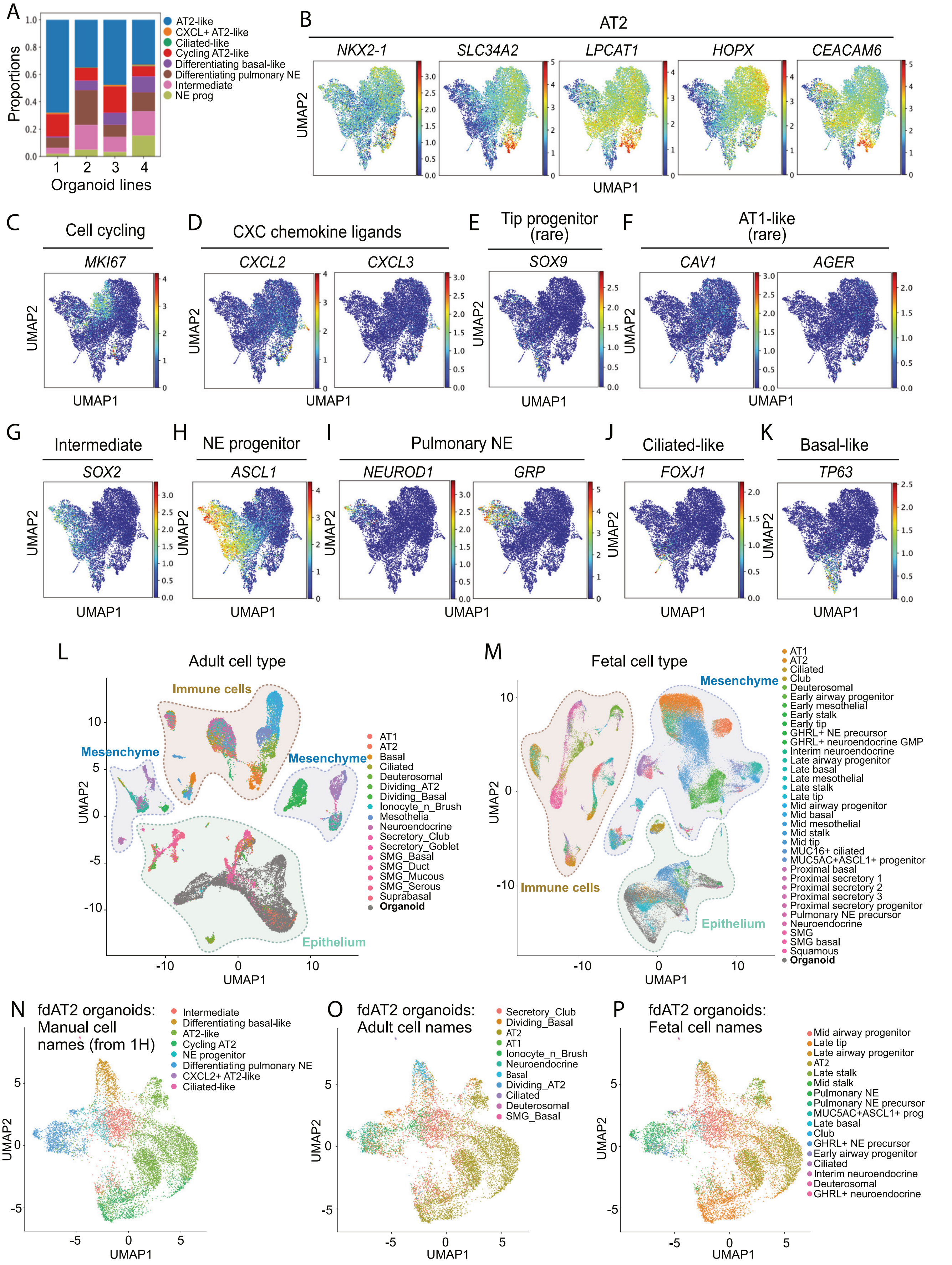
A
Proportions
Organoid lines
AT2-like
CXCL+ AT2-like
Ciliated-like
Cycling AT2-like
Differentiating basal-like
Differentiating pulmonary NE
Intermediate
NE prog
B
AT2
NKX2-1
SLC34A2
LPCAT1
HOPX
CEACAM6
UMAP2
UMAP1
C
Cell cycling
MKI67
D
CXC chemokine ligands
CXCL2
CXCL3
E
Tip progenitor (rare)
SOX9
F
AT1-like (rare)
CAV1
AGER
G
Intermediate
SOX2
H
NE progenitor
ASCL1
I
Pulmonary NE
NEUROD1
GRP
J
Ciliated-like
FOXJ1
K
Basal-like
TP63
L
Adult cell type
Immune cells
Mesenchyme
Mesenchyme
Epithelium
AT1
AT2
Basal
Ciliated
Deuterosomal
Dividing_AT2
Dividing_Basal
Ionocyte_n_Brush
Mesothelia
Neuroendocrine
Secretory_Club
Secretory_Goblet
SMG_Basal
SMG_Duct
SMG_Mucous
SMG_Serous
Suprabasal
Organoid
M
Fetal cell type
Mesenchyme
Immune cells
Epithelium
AT1
AT2
Ciliated
Club
Deuterosomal
Early airway progenitor
Early mesothelial
Early stalk
Early tip
GHRL+ NE precursor
GHRL+ neuroendocrine GMP
Interim neuroendocrine
Late airway progenitor
Late basal
Late mesothelial
Late stalk
Late tip
Mid airway progenitor
Mid basal
Mid mesothelial
Mid stalk
Mid tip
MUC16+ ciliated
MUC5AC+ASCL1+ progenitor
Proximal basal
Proximal secretory 1
Proximal secretory 2
Proximal secretory 3
Proximal secretory progenitor
Pulmonary NE precursor
Neuroendocrine
SMG
SMG basal
Squamous
Organoid
N
fdAT2 organoids: Manual cell names (from 1H)
Intermediate
Differentiating basal-like
AT2-like
Cycling AT2
NE progenitor
Differentiating pulmonary NE
CXCL2+ AT2-like
Ciliated-like
O
fdAT2 organoids: Adult cell names
Secretory_Club
Dividing_Basal
AT2
AT1
Ionocyte_n_Brush
Neuroendocrine
Basal
Dividing_AT2
Ciliated
Deuterosomal
SMG_Basal
P
fdAT2 organoids: Fetal cell names
Mid airway progenitor
Late tip
Late airway progenitor
AT2
Late stalk
Mid stalk
Pulmonary NE
Pulmonary NE precursor
MUC5AC+ASCL1+ prog
Late basal
Club
GHRL+ NE precursor
Early airway progenitor
Ciliated
Interim neuroendocrine
Deuterosomal
GHRL+ neuroendocrine

Figure EV2. Single-cell RNA sequencing analysis of the fdAT2 organoids.

(**A**) Graph showing the proportions of each cell type obtained from the four independent organoid lines. (**B–K**) UMAP plots showing transcript expression of AT2 lineage markers *NKX2-1*, *SLC34A2*, *LPCAT1*, *HOPX*, *CEACAM6* (**B**), *MKI67* (**C**), CXC chemokine ligands *CXCL2*, *CXCL3* (**D**), *SOX9* (**E**), AT1-like markers *CAV1*, *AGER* (**F**), *SOX2* (**G**), *ASCL1* (**H**), neuroendocrine cells *NEUROD1*, *GRP* (**I**), *FOXJ1* (**J**), *TP63* (**K**). (**L**, **M**) UMAPs showing fdAT2 organoid scRNA-seq data integrated with published adult (**L**) or fetal (**M**) scRNA-seq atlas data. FdAT2 organoid cells are shown in gray. (**N**, **O**) UMAPs showing fdAT2 organoid scRNA-seq with cell clusters named by manual annotation as in Fig. 1H (**N**), label transfer from the adult cell atlas (**O**), or label transfer from the fetal cell atlas (**P**).

 © The Author(s)

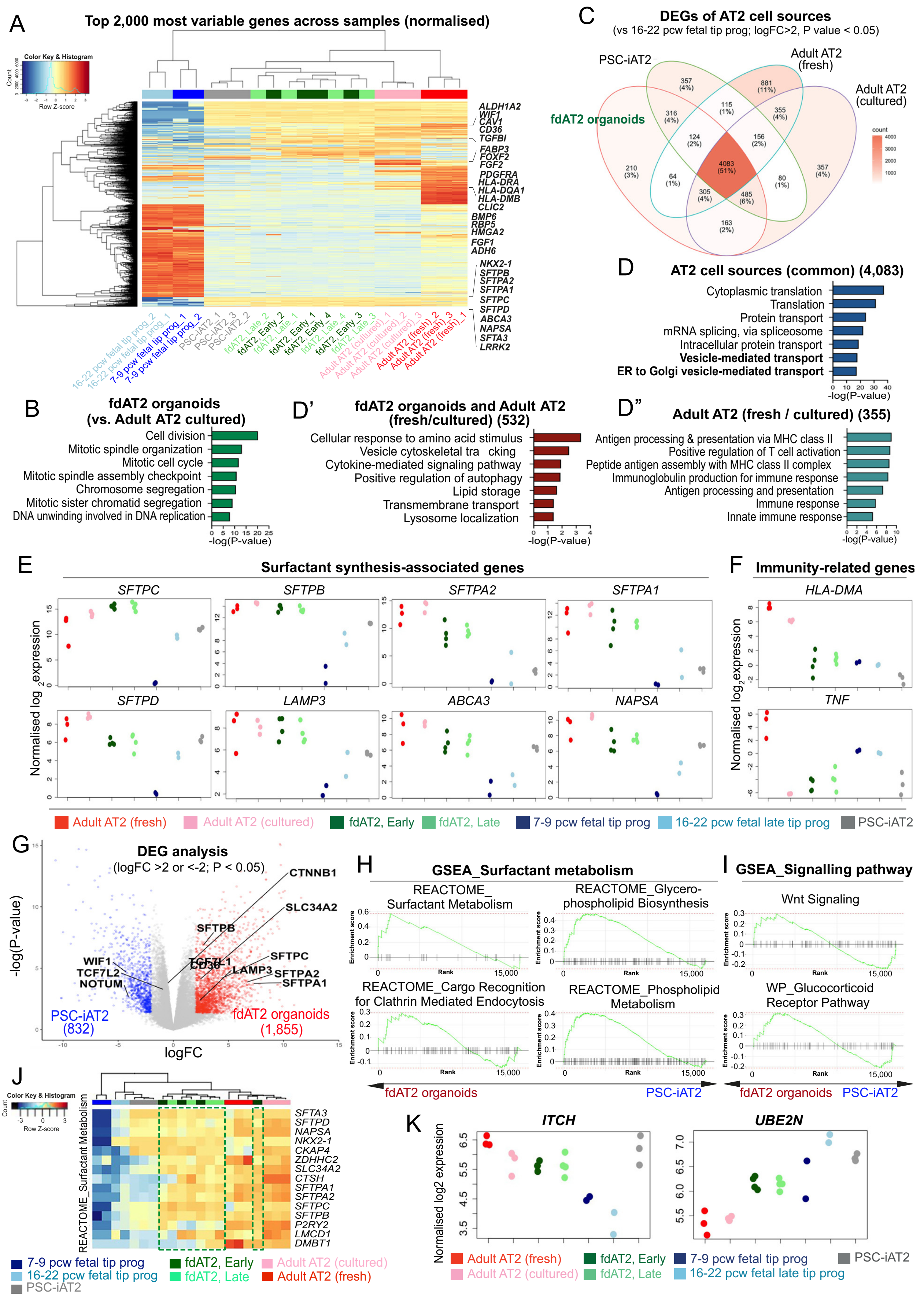
A
Top 2,000 most variable genes across samples (normalised)
B
fdAT2 organoids (vs. Adult AT2 cultured)
C
DEGs of AT2 cell sources
(vs 16-22 pcw fetal tip prog; logFC>2, P value < 0.05)
D
AT2 cell sources (common) (4,083)
D'
fdAT2 organoids and Adult AT2 (fresh/cultured) (532)
D"
Adult AT2 (fresh / cultured) (355)
E
Surfactant synthesis-associated genes
F
Immunity-related genes
G
DEG analysis
(logFC >2 or <-2; P < 0.05)
H
GSEA_Surfactant metabolism
I
GSEA_Signalling pathway
J
K

Figure EV3. Comparative transcriptomic analysis of fetal-derived AT2 organoids with other AT2 sources.

(**A**) Heatmap analysis of the top 2000 most variable genes across all samples. (**B**) GO analysis of genes highly enriched in the fdAT2 organoids compared to the cultured adult AT2 cells. (**C**) Venn diagram illustrating the number and the proportion of unique or shared genes from AT2 cells of different sources. The genes for each AT2 cell type that were differentially expressed compared to fetal 16–22 pcw tip progenitor organoids were included ($log_2FC > 2$, *P* value < 0.05; Dataset EV2). (**D**, blue) GO analysis of DEGs shared between AT2 cells of different origin, including fdAT2 organoids, PSC-iAT2, and cultured and freshly isolated adult AT2 cells; related to Fig. 1L. 4083 genes commonly shared by all AT2 fate cell types. (**D′**, red) genes shared by fdAT2 organoids and cultured and/or freshly isolated adult AT2 cells (**D″**, cyan) genes shared by cultured and freshly isolated adult AT2 cells. All GO analysis was performed using DEGs with $log_2FC > 2$, *P* value < 0.05; *P* values for each pairwise comparison are shown in Datasets EV1–3. (**E**, **F**) Expression levels of surfactant synthesis-associated genes (**E**) and immunity-related genes (**F**) across the different AT2 cell sources. (**G**) Volcano plot describing the direct comparison of fdAT2 organoids and PSC-iAT2. 1855 and 832 genes were differentially enriched in AT2 organoids and PSC-iAT2, respectively (logFC > 2, P value < 0.05; related to Dataset EV3). (**H**, **I**) Gene set enrichment analysis (GSEA) of surfactant metabolism and signaling pathway-associated gene sets between fdAT2 organoids and PSC-iAT2. (**J**) Heatmap of a gene set associated with surfactant metabolism from REACTOME. Green box, fdAT2 organoids. (**K**) Relative expression of E3 ligases *ITCH* and *UBE2N* in AT2 cells and tip progenitor organoids.

 © The Author(s)

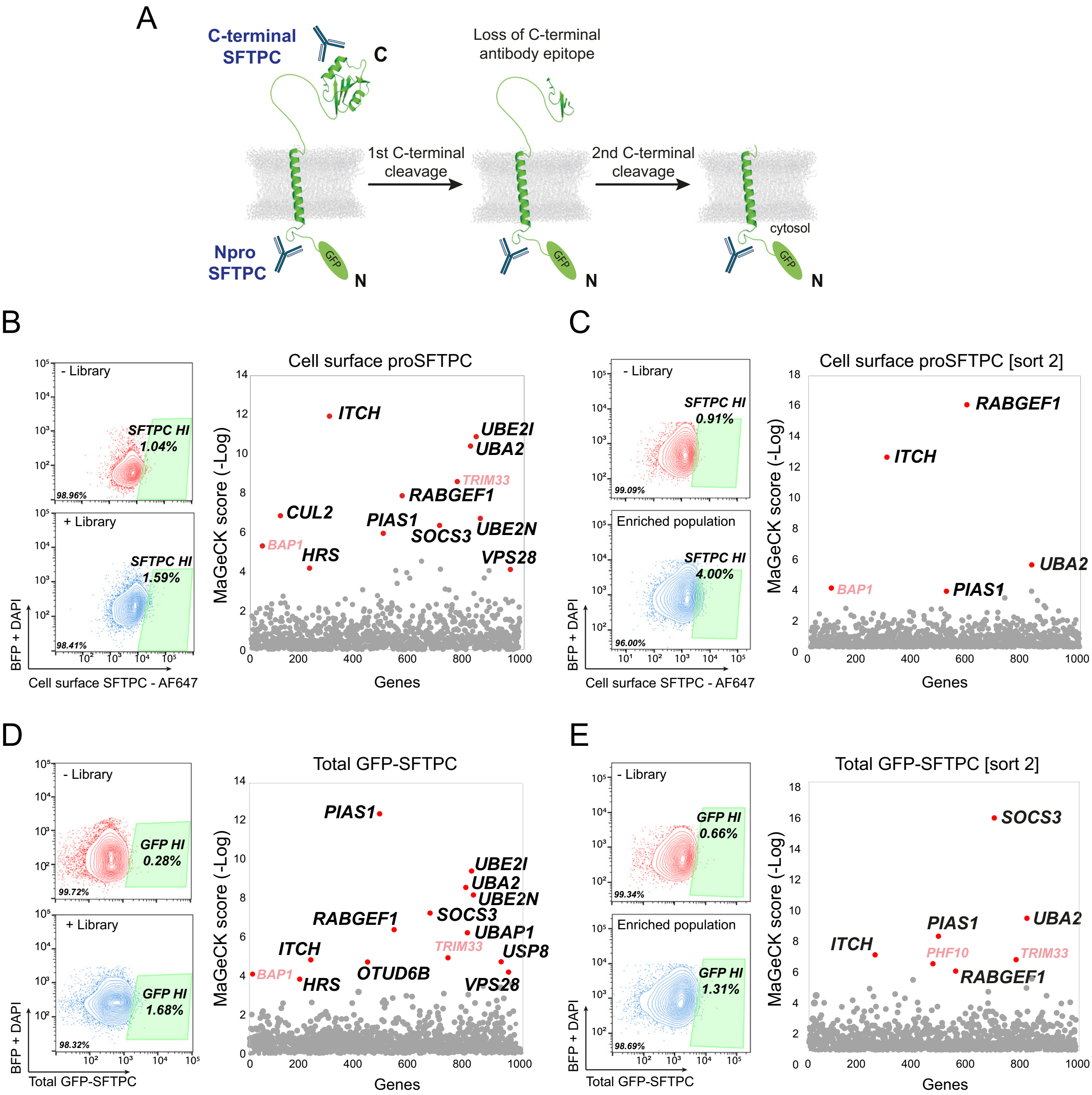

Figure EV4. A forward genetic screen identifies candidate proteins involved in SFTPC processing and trafficking.

(**A**) Schematic showing location of GFP tag and epitopes for N-terminal and C-terminal SFTPC antibodies. Only full-length proSFTPC is recognized by the C-terminal antibody used for flow cytometry assays. (**B–E**) Flow cytometry gating strategy and MAGeCK relative enrichment scores for genes whose depletion results in increased cell surface SFTPC (**B**, day 7 and **C**, day 14) or increased total eGFP-SFTPC (**D**, day 7 and **E**, day 14) post transduction with ubiquitome sgRNA library. Genes highlighted red (BAP1,TRIM33 and PHF10) are commonly enriched but non-specific transcription-related hits from forward genetic screens.

© The Author(s)

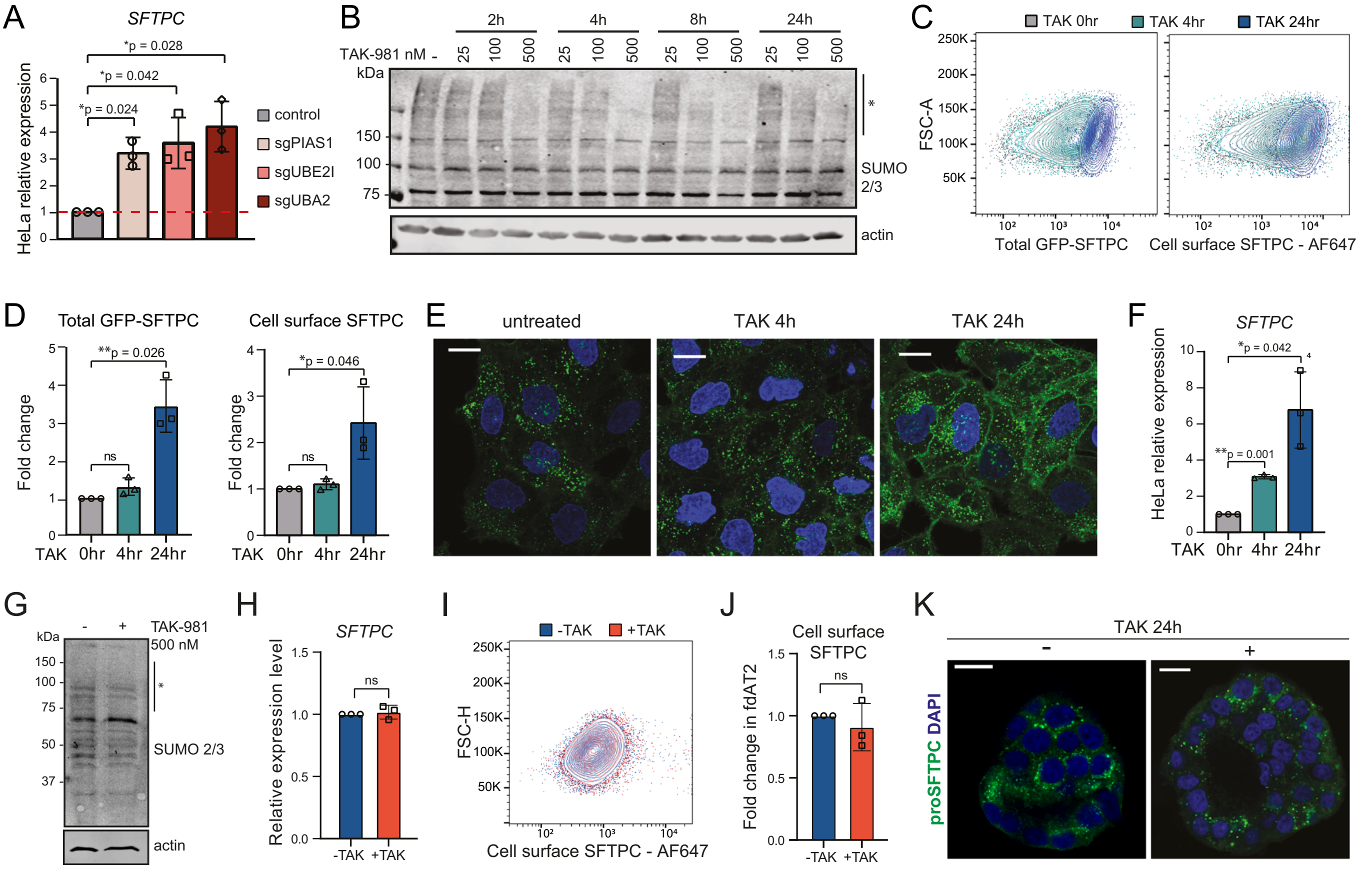

Figure EV5. SUMOylation is not involved in the expression or maturation of SFTPC when expressed from an endogenous promoter.

(**A**) Relative expression of SFTPC mRNA in GFP-SFTPC-Cas9 control cells and UBE2I, UBA2, and PIAS1 knockout pool; mean ± SD, $n = 3$ independent repeats (one-way ANOVA with Tukey multiple comparison post-test). (**B**) SUMO 2/3 immunoblot of TAK-981-treated HeLa cells (SUMOylated proteins are seen as a smear of higher molecular weight species (*) HeLa cells treated with 500 nM TAK-981 were assessed for total and cell surface full-length SFTPC as measured by flow cytometry (**C**, **D**) and live cell confocal microscopy (**E**), and for relative expression of SFTPC mRNA (**F**); mean ± SD, $n = 3$ independent repeats (one-way ANOVA with Tukey multiple comparison post-test). Scale bar, 20 µm. (**G**) SUMO 2/3 immunoblot of fdAT2 treated with 500 nM TAK-981 for 24 h. * = Smear of sumoylated proteins. fdAT2 were treated with 500 nM TAK-981 for 24 h, assessed for relative expression of SFTPC mRNA (**H**), total and cell surface SFTPC as measured by flow cytometry (**I**, **J**) and microscopy (**K**); mean ± SD $n = 3$ biologically independent organoid lines (one-way ANOVA with Tukey multiple comparison post-test). Scale bar, 10 µm. Source data are available online for this figure.

 © The Author(s)
